# Transcriptomic buffering of cryptic genetic variation contributes to meningococcal virulence

**DOI:** 10.1186/s12864-017-3616-7

**Published:** 2017-04-07

**Authors:** Biju Joseph Ampattu, Laura Hagmann, Chunguang Liang, Marcus Dittrich, Andreas Schlüter, Jochen Blom, Elizaveta Krol, Alexander Goesmann, Anke Becker, Thomas Dandekar, Tobias Müller, Christoph Schoen

**Affiliations:** 1 Institute for Hygiene and Microbiology, Joseph-Schneider-Straße 2, University of Würzburg, 97080 Würzburg, Germany; 2 Department of Bioinformatics, Biocenter, University of Würzburg, Am Hubland, 97074 Würzburg, Germany; 3 Department of Human Genetics, Biocenter, University of Würzburg, Am Hubland, 97074 Würzburg, Germany; 4 Center for Biotechnology (CeBiTec), Bielefeld University, Universitätsstr. 27, 33615 Bielefeld, Germany; 5 Institute for Bioinformatics and Systems Biology, Justus Liebig University Gießen, Heinrich-Buff-Ring 58, 35392 Gießen, Germany; 6 LOEWE-Center for Synthetic Microbiology, Hans-Meerwein-Straße, 35032 Marburg, Germany

**Keywords:** *Neisseria meningitidis*, Virulence, Regulatory evolution, Systems biology, Metabolism, Cryptic genetic variation, Stringent response, MITE, RelA

## Abstract

**Background:**

Commensal bacteria like *Neisseria meningitidis* sometimes cause serious disease. However, genomic comparison of hyperinvasive and apathogenic lineages did not reveal unambiguous hints towards indispensable virulence factors. Here, in a systems biological approach we compared gene expression of the invasive strain MC58 and the carriage strain α522 under different ex vivo conditions mimicking commensal and virulence compartments to assess the strain-specific impact of gene regulation on meningococcal virulence.

**Results:**

Despite indistinguishable ex vivo phenotypes, both strains differed in the expression of over 500 genes under infection mimicking conditions. These differences comprised in particular metabolic and information processing genes as well as genes known to be involved in host-damage such as the nitrite reductase and numerous LOS biosynthesis genes. A model based analysis of the transcriptomic differences in human blood suggested ensuing metabolic flux differences in energy, glutamine and cysteine metabolic pathways along with differences in the activation of the stringent response in both strains. In support of the computational findings, experimental analyses revealed differences in cysteine and glutamine auxotrophy in both strains as well as a strain and condition dependent essentiality of the (p)ppGpp synthetase gene *relA* and of a short non-coding AT-rich repeat element in its promoter region.

**Conclusions:**

Our data suggest that meningococcal virulence is linked to transcriptional buffering of cryptic genetic variation in metabolic genes including global stress responses. They further highlight the role of regulatory elements for bacterial virulence and the limitations of model strain approaches when studying such genetically diverse species as *N. meningitidis*.

**Electronic supplementary material:**

The online version of this article (doi:10.1186/s12864-017-3616-7) contains supplementary material, which is available to authorized users.

## Background

The human body is home to a vast number of different bacterial species, and the overwhelming complexity of the human microbiome has only very recently been fully uncovered [1]. Although the majority of these colonizing bacteria seem to be harmless or even beneficial commensals, some have long been known to be Janus-faced, and *Neisseria meningitidis* is a particularly prominent example in this respect. On the one hand, this β-proteobacterium is an exclusively human-adapted commensal that is carried in the nasopharynx of about 20% of the healthy population [2]. On the other hand, *N. meningitidis* is also a ferocious pathogen that can cause life-threatening invasive meningococcal disease (IMD), and “no other infection so quickly slays” [3]. After crossing the mucosal barrier and entering the bloodstream, meningococci can cause septicemia, and by crossing the blood–brain barrier and multiplying in the cerebrospinal fluid (CSF) also acute bacterial meningitis, both often within less than 24 h [4].

In many commensal pathogens like *Escherichia coli*, often the only difference between a pathogenic and a non-pathogenic strain is a small set of so called virulence genes [5]. By definition, a virulence gene is a gene whose loss specifically impairs virulence but not viability in rich media and which should be associated exclusively with pathogenic but not with non-pathogenic strains of a species [6]. However, with respect to gene content meningococcal strains isolated from healthy carriers and IMD patients are almost indistinguishable [7], and many of the so called meningococcal virulence genes have also been found in purely commensal neisserial species [8]. The analysis of meningococcal population genetic structure by multilocus sequence typing (MLST) demonstrated that disease-causing meningococci do belong to particular groups of related sequence types (STs), termed clonal complexes (CCs), which are overrepresented in disease isolates relative to their carriage prevalences and are responsible for most disease [2]. Accordingly, these data indicate that the propensity to cause invasive disease is somehow associated with the genetic make-up of hyperinvasive lineages. Experimental observations along with epidemiological models further indicate that genetic differences in metabolic genes might have a central role in the observed virulence differences among different lineages in a yet to define manner [9, 10].

Alongside the well-established significance of gene content variation in creating genetic diversity in the bacterial world, regulatory evolution is increasingly acknowledged to substantially contribute to this diversity [11]. Mutations affecting gene expression regulation encompass differences in the coding sequences of transcription factors (TF) acting *in trans* and thus affecting the expression of entire regulons [12, 13], as well as sequence differences in the regulatory regions acting *in cis* on the expression of downstream genes (e.g. [14]). By acquiring functionally divergent homologous promoter regions through horizontal transfer bacterial genes were shown to rapidly shift between multiple regulatory modes affecting, for example, up to 15% of the meningococcal core genome [15]. In addition, also mutations in metabolic genes can indirectly cause compensatory changes in gene expression regulation of other housekeeping genes to maintain cellular homeostasis. The ensuing differential regulation of conserved genes can mediate phenotypic traits that distinguish closely related bacterial species [16] or even strains of the same species [17]. In consequence, also the expression of a virulence-associated gene could be epistatic and thus depend on the genetic background of the respective strain (gene-gene interaction, G x G) [18] and/or the environment (gene-environment interaction, G x E) [19]. Given the high genetic diversity of *N. meningitidis* [2], a virulence gene candidate should consequently be differently expressed between an invasive and a commensal strain under disease mimicking conditions but not under conditions mimicking the commensal state. In addition, although the correlation between when genes are important for fitness and when those genes are upregulated was shown to be small [20], the fitness of a knock-out strain should differ between an invasive and a commensal strain (G × G) under conditions mimicking invasive infection (G × E).

The strict tropism of *N. meningitidis* for humans has so far impeded the development of a suitable animal model to analyze the course of meningococcal infection in vivo, and therefore alternative experimental approaches such as ex vivo models have been established to study meningococcal infection biology. For example, human whole blood served as an ex vivo model to analyze how meningococci regulate gene expression to permit survival in human bloodstream during septicemia [21, 22]. Likewise, meningococcal resistance to human complement was studied in an ex vivo model using human CSF [23], and human saliva has already been used to study ex vivo the transcriptional response which enables meningococci to adapt to this relevant host niche [24]. However, most of these ex vivo studies analyzed gene expression only under a single condition and/or used only a single strain from a hyperinvasive lineage. Consequently, our knowledge about how this commensal pathogen adapts during the transition from colonization to an invasive infection is still very limited, and nothing is known so far about gene expression or phenotypic variability between carriage and hyperinvasive strains in conditions mimicking invasive infection. In a systems biological approach we used different ex vivo conditions as environmental perturbation and natural genetic variation as genetic perturbation of the meningococcal gene expression network and considered gene expression as quantitative intermediate phenotype [25, 26]. Based on prior population genetic information we selected two genetically related meningococcal serogroup B strains from the same phylogenetic clade PC32/269 [27, 28] with yet markedly different epidemiology (Table 1 and Additional file 1: Figure S1) [2]. Strain MC58 belonging to the hyperinvasive ST-32 CC was chosen as a reference as this strain has already served as a model system to experimentally study meningococcal infection biology in vitro and a large body of transcriptomic data is thus available [21, 22, 29–34]. The carriage strain α522 belongs to the carriage ST-35 CC which is a four locus variant of the ST-32 CC sharing about 96% of its genes with strain MC58 [28]. We combined phenotypic, genomic and transcriptomic comparisons with mutagenesis studies to seek genetic variants that influence meningococcal gene expression in human saliva, whole blood and CSF mimicking commensal and virulence compartments, respectively. Gene expression was analyzed in a strain- and condition-dependent manner with particular emphasis on virulence-associated genes and genes involved in gene expression regulation. The combined data show that transcriptomic buffering of cryptic genetic variation, which is the genetic variation present in the meningococcal population that is not phenotypically expressed under commensal conditions but visible upon environmental or genetic perturbations such as growth in human blood [35, 36], contributes to the regulatory evolution of meningococcal virulence. We further demonstrate that it is likely affected by the differential presence of a short, non-coding inverted-repeat transposable-element in the promoter region of *relA* encoding the guanosine 3'-(tri)diphosphate 5-'diphosphate ((p)ppGpp) synthetase of the stringent response pathway.

**Table 1:** Strains used for ex vivo transcriptome comparisons

	α522	MC58
Genome characteristics		
GenBank accession number	FR845693 to FR845718	AE002098
No. of contigs in final assembly	21	1
Average single base coverage	79-fold	8.4-fold
Genome size (bp)	≥2,074,170	2,272,360
GC content (%)	51.78%	51.53%
Predicted number of coding sequences	≥1985	2063
Reference	This work	Tettelin et al. (2000) [123]
Molecular epidemiology		
Source	Carrier	Patient
Country and year of isolation	Germany 2000	United Kingdom 1983
Sequence type	ST-35	ST-74
Clonal complex (CC)	ST-35	ST-32
Phylogenetic clade (PC) ^(a)^	PC32/269	PC32/269
Frequency of CC in carriers^(b)^	5.47%	4.99%
Disease/carriage ratio^(c)^	0.5	3.5
Reference	Claus et al. (2005) [100]	McGuiness et al. (1991) [101]
Phenotypic characterization		
Serum resistance (%)^(d)^	117.7 ± 23.8	116.3 ± 15.7
Adhesion to epithelial cells^(e)^		
FaDu cells (%)	10.7 ± 7.4	14.5 ± 8.5
Detroit562 cells (%)	8.9 ± 1.8	17.1 ± 5.3
Invasion of epithelial cells^(e)^		
FaDu cells (%)	0.0008 ± 0.0001	0.0020 ± 0.0011
Detroit562 cells (%)	0.0011 ± 0.0004	0.0016 ± 0.0007
In vitro logarithmic growth rates^(f)^		
Rich medium (PPM+) (1/h)	0.46 ± 0.01	0.47 ± 0.01
Minimal medium (MMM) (1/h)	0.06 ± 0.01	0.52 ± 0.02
Ex vivo growth rates^(g)^		
Saliva (1/min)	−0.041 ± 0.003	−0.043 ± 0.003
Blood (1/min)	0.027 ± 0.004	0.023 ± 0.002
CSF (1/min)	0.010 ± 0.002	0.018 ± 0.003

^(a)^ According to ref. [27, 28]

^(b)^ According to ref. [100]

^(c)^ According to ref. [2]

^(d)^ Ratio in percent of viable bacteria after incubation for 30 min in the presence of 10% human serum and viable bacteria incubated without serum. Given are the average and standard deviation from four independent experiments with pooled human serum

^(e)^ Ratio in percent of adherent and invasive bacteria, respectively, to total bacteria. Given are the average and standard deviation from at least four independent experiments

^(f)^ Given are the mean and standard deviation of the logarithmic growth rate *k* according to log(*OD*_*600*_(*t*)/*OD*_*600*_(0)) = *kt* for *t* ∈ [1 h, 4 h] as depicted in Fig. 5 using linear regression (*R* ^2^_*in vitro*_ = 0.96 ± 0.07, *p*_*in vitro*_ = 0.013 ± 0.026)

^(g)^ Given are the mean and standard deviation of the growth rate *k* according to log(*N*(*t*)/*N*(0)) = *kt* for *t* ∈ [0 min, 120 min] as depicted in Fig. 5 using linear regression (*R* ^2^_ex vivo_ = 0.95 ± 0.05, *p*_ex vivo_ = 0.018 ± 0.016)

## Results

### MC58 and α522 have similar gene content including most genes invovled in host interactions but with function-dependent sequence variation among orthologs

In order to comprehensively analyze genetic differences between both strains we generated a draft sequence of the α522 genome for comparative genome expression analyses (Table 1, Additional file 1: Figure S2A). The common genomic backbone of both strains comprises 1.93 Mbp and encodes 1757 orthologous proteins with an average BLASTP bit score ratio (BSRP) of 0.958 (95%-confidence interval (CI) = [0.513, 1.000]), corresponding to an average amino acid identity of 99.4% (95%-CI = [81.6%, 100%]). Notably, there was a significant variation of the BSRPs of orthologous proteins with respect to the COG functional category [37] (Kruskal-Wallis rank sum test, *p* < 0.05) (Additional file 1: Figure S2B). The 10% most divergent orthologous genes (BSRP < 0.933, *n* = 177) were significantly enriched for genes involved in cell motility (COG N, odds ratio (OR) = 3.8, false discovery rate (FDR) = 0.031, Fisher’s exact test with Benjamini-Hochberg multiple testing correction) as well as secretion and transport (COG U, OR = 2.6, FDR < 0.05). The latter included numerous surface antigens involved in host interactions such as the major type IV pilus subunit protein PilE, the major outer membrane proteins PorA and PorB, the autotransporters App (NMB1985) and NalP (NMB1969), as well as proteins involved in iron acquisition like the transferrin-binding protein B and the lactoferrin-binding protein B. Compared to the 10% most divergent orthologous genes, identical genes (*n* = 825) were significantly enriched for genes involved in energy production and conversion (COG C, OR = 6.9, FDR < 0.01), carbohydrate transport and metabolism (COG G, OR > 1.6, FDR < 0.05), amino acid biosynthesis (COG E, OR = 1.7, FDR < 0.01) and translation, ribosomal structure and biogenesis (COG J, OR = 5.3, FDR < 0.001) (see Additional file 2: S1). Of the 135 genes coding for putative virulence factors or involved in meningococcal host interactions in strain MC58 (compiled from ref. [21, 38–40], see Additional file 2: S1) only 8 are missing in strain α522, and strain α522 lacks in particular large parts of the islands of horizontal transfer B and C that code for a two-partner secretion system involved in host cell adhesion [41] (Additional file 1: Figure S2A). It further lacks almost the entire repeat-in-toxin island 1 encoding FrpA/C-like proteins which induce high levels of serum antibodies during invasive disease in humans [42]. In addition, downstream of *glnB* encoding the signal-transducing nitrogen regulatory protein PII strain α522 also lacks *nadA* which codes for a minor adhesin that was found to promote bacterial adhesion to and penetration into human epithelial cells in vitro [43]. MC58 further harbors a 30 kb duplication spanning 37 coding sequences involving the *cysGHDNJI* genes for sulfur acquisition which is missing in α522 as in most other meningococcal genomes.

### Both strains display similar phenotypes in ex vivo fitness and in vitro cell culture assays indicative of phenotypic buffering

Despite the genetic differences described above, both strains were phenotypically similar with respect to resistance against human serum, adhesion to and invasion of nasopharyngeal cell lines, respectively, as well as in ex vivo growth (Table 1). Surprisingly, although meningococci are part of the human oral microbiome both strains were not able to grow in human saliva. Since strain MC58 was able to grow in meningococcal minimal medium (MMM), the observed growth inhibitory effect of human saliva was probably not due to nutrient limitation but more likely caused by the presence of growth inhibiting substances known to be present in human saliva like antimicrobial peptides [44]. Of note, in their ecological niche, the human nasopharynx, meningococci are attached to host cells in the form of microcolonies and are unlikely to grow in a planktonic state as in the ex vivo situation [45, 46]. The fact that bacteria in biofilms are less susceptible to antimicrobial agents and host immune responses thereby becoming persistent colonizers [47] might explain the unexpected ex vivo growth phenotype in saliva. Furthermore, both strains grew equally well in human blood and CSF and thus under disease mimicking conditions despite their about 7-fold difference in the disease/carriage ratio (Table 1). These data indicate that the ability to survive in human blood is obviously necessary but per se not sufficient to cause IMD.

We therefore focused on compensatory mechanisms which might buffer the genotypic differences described above under ex vivo conditions and considered the transcriptome as an intermediate phenotype [25, 26]. We hypothesized that both strains differ in the way they accomplish growth under disease mimicking conditions by differential transcriptional activation of metabolic and stress response pathways. For convenience, comparisons of the MC58 transcriptomes between saliva, whole blood and CSF, respectively, will be called cross-condition comparisons and discussed only briefly in the main text and in more detail in the Additional file 1. Likewise, comparisons of the transcriptomes of strain MC58 and α522 in saliva, whole blood and CSF, respectively, will be called cross-strain comparisons (Additional file 1: Figure S1). Genes differently expressed between two conditions or between both strains in a given condition will be called differently expressed genes, and genes differently regulated between both strains between two conditions will shortly be called differently regulated genes. Throughout the following analyses, we further discern directional and non-directional classes of gene sets. The non-directional class of significantly differently expressed genes contains genes where the information about direction of differential expression is omitted, so that significant gene sets can be interpreted as affected by differential expression in general. The directional class aims to identify gene sets that are significantly affected by regulation in a distinct direction, i. e. condition or strain. If a gene set contains significantly expressed genes in both directions, they will cancel out and the directional FDR-value will not be significant. In turn, if a gene set is not significantly enriched for differently expressed genes in general it might nonetheless comprise mainly genes with a significant expression bias in one direction. It consequently will have a non-significant non-directional FDR-value but a significant directional FDR-value.

### Condition-dependent expression changes affect 24% of the MC58 transcriptome

**Fig. 1:**
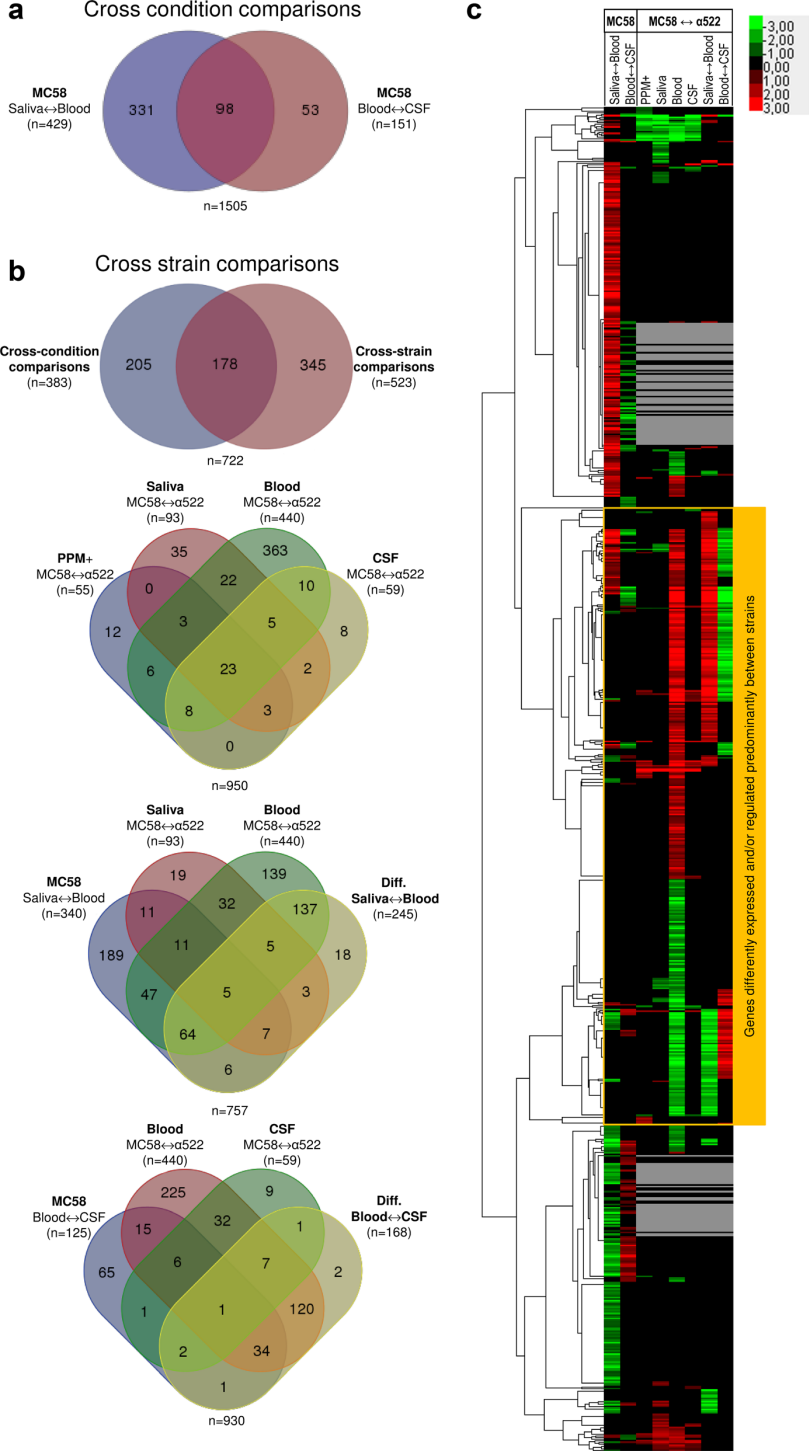
Genes significantly differently expressed and/or regulated in cross-condition and/or cross-strain comparisons. **a** Venn diagram comparing sets of genes in strain MC58 differently expressed between conditions as indicated. The total number of genes compared was 1987. **b** Venn diagrams comparing sets of genes differently expressed between strains as indicated with each diagram. The total number of genes compared in each panel was 1450. **c** Heatmap depicting cross-condition and cross-strain gene expression differences and hierarchical clustering of significantly differently expressed genes. Average linkage clustering based on the Spearman rank correlation of all 828 genes significantly differently expressed and/or regulated in at least one cross-condition and/or cross-strain comparison (FDR < 0.05). *Grey lines* correspond to genes that were absent in the α522 genome sequence and therefore excluded from the cross-strain comparisons

**Fig. 2:**
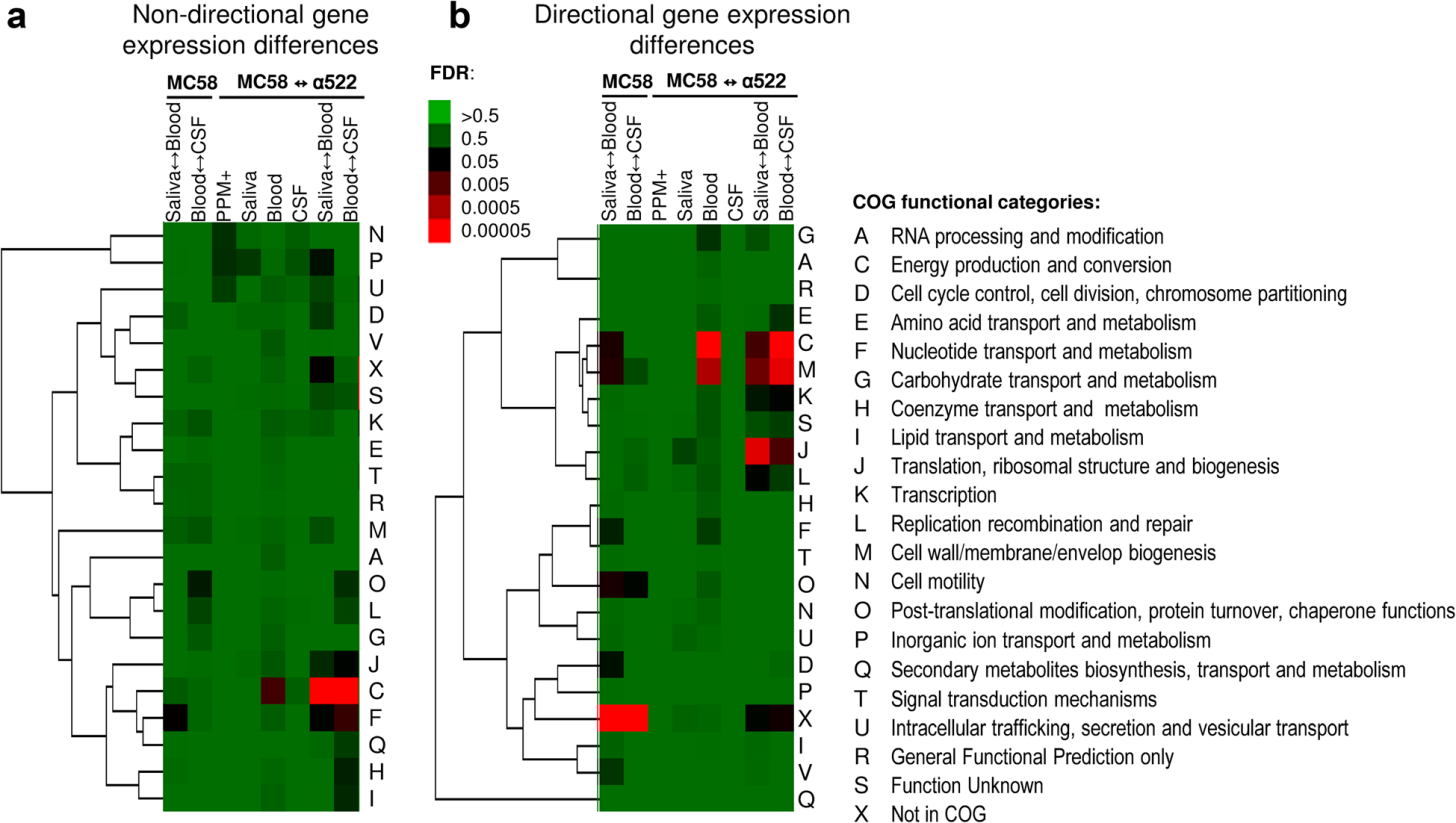
Gene set enrichment analysis of differently expressed genes. **a** Non-directional comparison of significantly differently expressed gene sets according to the COG functional classification scheme for cross-condition and cross-strain comparisons, respectively, indicating significantly overrepresented functional categories among the significantly differently expressed genes. **b** Directional comparison of significantly differently expressed gene sets according to the COG functional classification scheme for cross-condition and cross-strain comparisons, indicating significant gene expression asymmetries. In both panels, the heat map depicts significantly enriched COG functional categories in red coloring. The kind of comparison (cross-condition for strain MC58 and cross-strain for each condition) is indicated for each column of the heat maps, and the corresponding FDRs are color coded and given in the respective inserts. The associated tree is based on average linkage clustering of the functional categories using the Spearman rank correlation coefficient

Of the 1987 genes assayed in the cross-condition comparisons in strain MC58, 429 genes were differently expressed between saliva and blood and 151 between blood and CSF, respectively (Fig. 1a, c and Additional file 1: Figure S2A). Gene set enrichment analysis (GSA) showed that genes differently expressed between saliva and blood were significantly enriched for genes coding for nucleotide transport and metabolism (COG F) (Table 2), whereas genes differently expressed between blood and CSF were just slightly enriched for genes involved in posttranslational modification, protein turnover and chaperones (COG O, OR = 2.7, FDR > 0.10) (Fig. 2a). GSA further showed significant differences also in the directionality of gene expression changes (Fig. 2b). The 173 genes that showed higher expression levels in saliva compared to blood were enriched for genes involved in energy metabolism and conversion (COG C), posttranslational modification, protein turnover and chaperones (COG O) and cell envelope and outer membrane biogenesis (COG M). However, genes that were more highly expressed in blood than in saliva and CSF, respectively, were significantly enriched predominantly for genes coding for proteins without any COG functional annotation so far, suggesting that our knowledge about the mechanisms employed by meningococci to survive in human blood are still quite limited.

**Table 2:** Significantly enriched COG functional categories in cross-condition and cross-strain comparisons

	Non-directional^(a)^		Directional^(b)^	
COG functional category	OR^(c)^	FDR^(d)^	OR^(e)^	FDR^(d)^
MC58 cross-condition comparisons				
Saliva ↔ Blood				
Nucleotide transport and metabolism (COG F)	2.66	0.044	n.s.	n.s.
Energy metabolism and conversion (COG C)	n.s.	n.s.	0.18	0.024
Cell wall/membrane/envelope biogenesis (COG M)	n.s.	n.s.	0.31	0.020
Posttranslational modification, protein turnover, chaperones (COG O)	n.s.	n.s.	0.13	0.023
Not in COG	n.s.	n.s.	6.53	<0.001
Blood ↔ CSF				
Not in COG	n.s.	n.s.	0.06	<0.001
MC58 versus α522 cross-strain comparisons				
Blood				
Energy metabolism and conversion (COG C)	2.25	0.008	11.6	<0.001
Cell wall/membrane/envelope biogenesis (COG M)	n.s.	n.s.	0.19	<0.001
Saliva ↔ Blood				
Energy metabolism and conversion (COG C)	2.99	<0.001	>5.33	<0.001
Translation, ribosomal structure and biogenesis (COG J)	n.s.	n.s.	0.04	<0.001
Cell wall/membrane/envelope biogenesis (COG M)	n.s.	n.s.	0.09	0.003
Blood ↔ CSF				
Energy metabolism and conversion (COG C)	3.27	<0.001	n.s.	n.s.

^(a)^ Comparison of differently versus non-differently expressed genes to identify COG categories that are affected by differential expression in general

^(b)^ Comparison of differentially expressed genes to identify COG categories that are significantly affected by regulation in a distinct direction (gene expression asymmetry)

^(c)^ Odds ratios based on Fisher’s exact test. Values greater than 1 indicate that significantly differentially expressed genes are enriched for genes from the corresponding COG functional class

^(d)^ False discovery rate based on *p*-values from Fisher’s exact test and the Benjamini-Hochberg multiple testing correction with a significance cut-off of FDR < 0.05

^(e)^ For cross-condition comparisons, values greater than 1 indicate that genes highly expressed in blood were enriched for the respective COG category. In cross-strain comparisons, values greater than 1 indicate that the respective COG category is significantly highly expressed or upregulated in strain MC58

### Both strains differ in the expression of over 500 genes in a condition and strain dependent manner

Based on the genome comparisons we further selected a sub-set of 1450 single-copy orthologous genes for cross-strain gene expression comparisons. Of these, 523 were expressed and/or regulated at significantly different levels between both strains (Additional file 1: Figure S2A and Fig. 1b, c). Notably, proteins differently expressed in at least one ex vivo condition were not significantly more variable between both strains as indicated by their BSRPs than proteins that were not (BSRP_constant_ = 0.993 vs. BSRP_diff. expressed_ = 0.995, Wilcoxon test, *p* > 0.05), confirming that there was no significant sequence bias in the expression data.

Of all the 728 genes that showed cross-condition and/or cross-strain expression differences 345 were differently expressed only in cross-strain and 205 only in cross-condition comparisons, respectively, and the overlap between cross-condition and cross-strain expression comparisons was thus surprisingly small (Fig. 1b). The total number of significantly differently expressed genes between both strains was small in rich medium (PPM+) (*n* = 55) and CSF (*n* = 59) and not significantly greater than the number expected by chance (FDR < 0.05, binomial test, *p* > 0.10), and there were only 8 and 12 genes that showed significant expression level differences between both strains exclusively in CSF and PPM+, respectively. The number of differently expressed genes was significantly higher for saliva (*n* = 93) and blood (*n* = 440) (binomial test, *p* < 0.01) than expected by chance (FDR > 0.05), although both strains were not able to grow in saliva. This further suggests that gene expression differences were not due to growth rate differences but were specific responses of both strains to these two ex vivo conditions. In line with this hypothesis, the transcriptomes differed significantly among both strains in response to the ex vivo condition tested (Pearson’s *χ* ^2^ test, *p* < 10^-15^). Of note, the pattern of transcriptionally activated genes upon transition from saliva to blood was opposite to the pattern of transcriptionally activated genes upon transition from blood to CSF (Figs. 1b and 2b), indicating that transcriptional changes are likely in response to components present in human blood but neither in saliva nor CSF. Since these two ex vivo compounds were both free of any phagocytic cells, differences in transcriptional responses in both strains might be triggered by blood phagocytes.

### Cross-strain gene expression differences in saliva comprise numerous stress response genes

A total of 93 genes were differently expressed between both strains in saliva, with a slight yet not significant enrichment of genes involved in anorganic ion transport and metabolism (*n* = 10, COG P) (Fig. 2a). Functionally, around one third coded each for poorly characterized proteins (*n* = 27, COGs R, S and X), proteins involved in cellular processes (*n* = 34) or proteins involved in metabolism or information storage and processing. Among the 55 genes highly expressed in strain α522 were, amongst others, seven for anorganic ion transport and metabolism proteins (COG P) including a putative multidrug resistance protein (NMB0393), the lactoferrin-binding protein A (LbpA) and the putative ammonium transporter AmtB, as well as six genes involved in cell envelope biogenesis (COG M) including genes for the two sialic acid capsule biosynthesis proteins SynX/SiaA/CssA and SiaB/CssB. Genes involved in translation and ribosomal biogenesis (COG J) comprised the single largest group (*n* = 10) among the genes that were in turn highly expressed in strain MC58 in saliva, next to genes in amino acid transport and metabolism (*n* = 4, COG E) and cell envelope biogenesis (*n* = 4, COG M). Since both strains were not able to grow in human saliva these gene expression differences likely reflect differences in the stress response between both strains when exposed to this hostile environment.

### Both strains differed in the expression of virulence-associated genes involved in the pathogenesis of IMD

Numerous genes involved in the interaction of meningococci with its human host have so far been studied in order to understand the genetic and mechanistic basis of meningococcal virulence, i.e. host damage, and were consequently of special interest. Accordingly, of the 102 virulence-associated genes used for cross-strain comparisons (compiled from ref. [21, 38–40]) (Additional file 2: S1), 48 were differently expressed and/or regulated among both strains under at least one of the ex vivo conditions tested which is significantly more than in the cross-condition comparisons for strain MC58 (2-sample test for equality of proportions, *p* < 0.01) (Table 3). In addition, genes differently expressed and/or regulated between both strains in at least one cross-strain comparison were significantly enriched for virulence-associated genes (OR = 1.55, *p* < 0.05), and the expression profiles were significantly different between virulence-associated and not virulence-associated genes (Pearson’s *χ* ^2^ test, *p* < 10^−15^). Of the 38 virulence-associated genes that were differently expressed between both strains in blood, 16 were highly expressed in α522 and comprised genes involved in cell envelope biogenesis and in particular genes for capsule and LOS biosynthesis. LOS was shown to be the dominant molecule in meningococci inducing organ inflammation in human patients [48], and differences in LOS biosynthesis might therefore have an immediate impact on the extent of host damage caused by both strains. In turn, genes involved in type IV pilus biosynthesis iron homeostasis, the stress response genes as well as genes encoding adhesins such as Opc or the hemagglutinin/hemolysin-related protein TpsA3 or NspA [49] were all highly expressed in MC58. Besides Opc, we could at best detect only very small expression level differences for other recently introduced vaccine antigens that were conserved in both strains (NMB1030 and NMB2091).

Likewise, of the 37 genes that have been described so far as being involved in neutrophil interactions in *Neisseria* and which were part of the gene expression comparisons [38], 15 were differently expressed between both strains in blood (Additional file 2: S1). Genes differently expressed between both strains were therefore significantly enriched for neutrophil response genes (OR = 2.9, *p* < 0.01). These included a number of virulence-associated genes like capsule synthesis genes or genes coding for efflux pump components (Table 3), as well as genes not so far associated with meningococcal virulence like the DNA damage repair genes *uvrA* and *uvrB*. This finding indicates that neutrophils might have an important role in shaping the meningococcal transcriptional response to human blood.

Of note, *aniA* (also annotated as *panI*) encoding nitrite reductase and *norB* coding for NO reductase [50] showed the largest blood-specific expression differences between both strains (8- to 32-fold) and were among the highest expressed genes in MC58 as was also confirmed by qRT-PCR (Additional file 1: Figure S3). The gene products AniA and NorB constitute a pathway that enables the organism to grow under conditions of low oxygen in the presence of nitrite. Amongst others, meningococcal derived NO was recently shown to play an essential role in the pathophysiology of septicemic meningococcal infection in humans by inhibiting platelet aggregation [51] and modifying the release of cytokines and chemokines by human macrophages [52].

Therefore, a number of virulence-associated genes with an also experimentally established role in the pathogenesis of IMD like *aniA* or LOS biosynthesis genes were differently expressed in both strains particularly in human blood.

### Transcriptomic differences between both strains associated with the transition from saliva to blood are enriched for metabolic, information processing and cell envelope biogenesis genes

Overall, the number of differently expressed genes between both strains was highest in blood (*n* = 440), and also the number of genes differently regulated between both strains between two conditions was highest between saliva and blood (*n* = 245) (Fig. 1). Both gene sets were functionally enriched for genes required for energy production and conversion (COG C) (Fig. 2a, Table 2). These data thus clearly demonstrate differences in the environment-dependent gene-expression regulation between both strains. The 196 genes highly expressed in strain α522 in human blood were significantly enriched for genes involved in cell wall/membrane biogenesis (COG M) (Fig. 2b, Table 2), including LOS and peptidoglycan biosynthesis genes like *murB*, *murD*, *murE* and *ddl*. In contrast, the 244 genes highly expressed in strain MC58 were significantly enriched for metabolic genes (COG C) including genes for oxidative phosphorylation (*nqrBDF*) and nitrogen respiration such as *aniA* and *norB* described above.

With respect to differences in the direction of gene expression regulation in both strains, genes required for energy production and conversion (COG C) were also strongly upregulated in strain MC58 between saliva and blood, whereas genes for cell envelope biogenesis (COG M) and translation (COG J) were in turn strongly upregulated in strain α522.

Based on differences in the directionality of gene expression levels and regulation (Fig. 2b), the functional categories COG E, C and M form a cluster of co-regulated genes which is part of a larger cluster including also the functional categories COG J, COG K and COG L. This finding suggests a regulatory link between metabolism, the biosynthesis of the cell envelope, and genes for the gene expression machinery which is differently activated in both strains particularly in human blood.

### Integrative network analysis of differently expressed genes identifies subnetworks of co-regulated genes

**Fig. 3:**
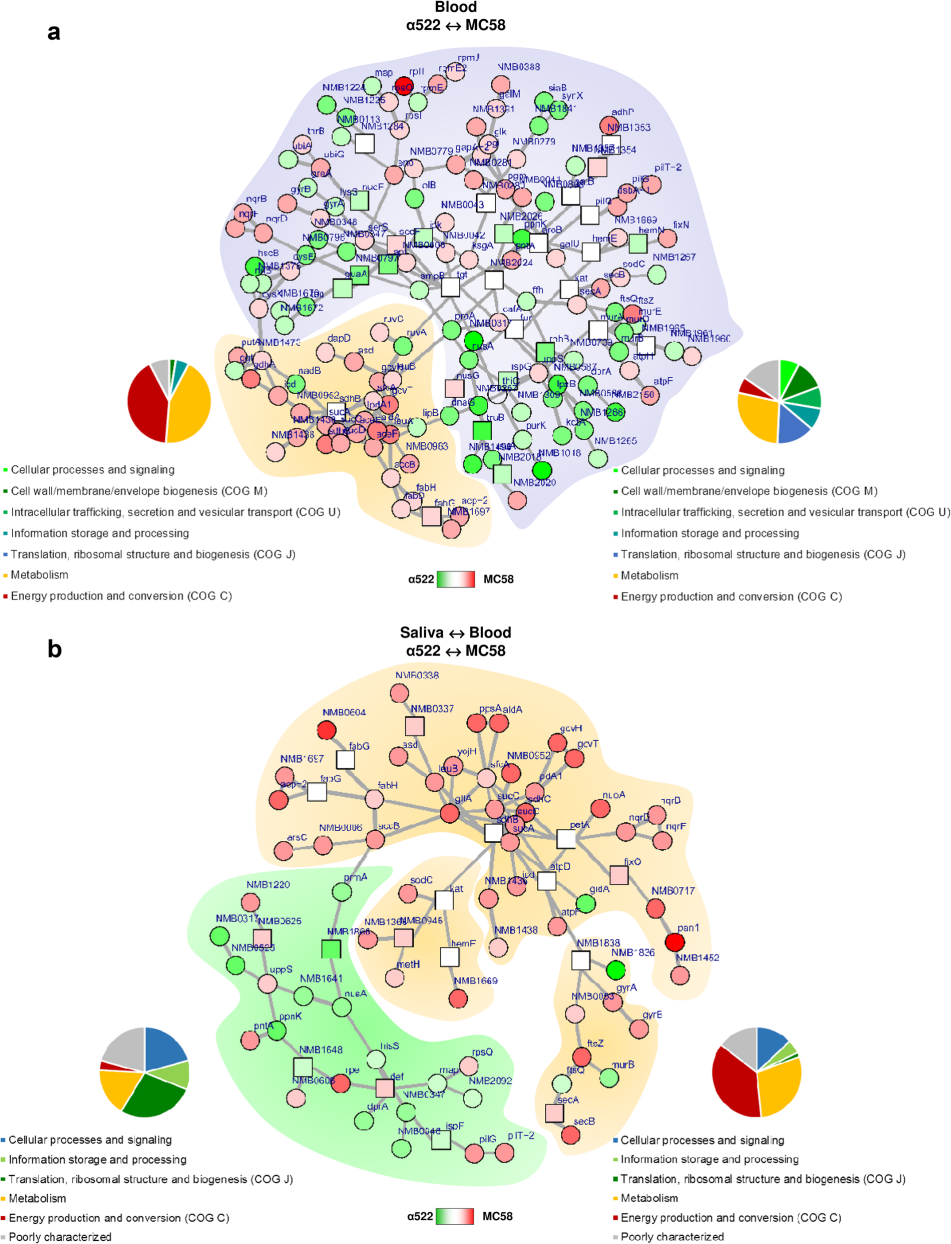
Analysis of gene expression data based on protein-protein interaction networks. **a** Integrative network analysis of differently expressed genes between strain MC58 and α522 in human whole blood based on the STRING protein-protein interaction network for strain MC58 (FDR < 10^−9^). A subnetwork that consists predominantly (30/35) of genes that were expressed at higher levels in MC58 than in α522 and that code for metabolic genes and in particular for genes involved in energy production and conversion (85%) is shaded in *orange*. The remaining part of the network comprising 128 protein-coding genes is shaded in *light blue*. **b** Integrative network analysis of gene regulation differences between both strains upon transition from saliva to blood (FDR < 10^−7^). The two modules consisting predominantly of genes either upregulated in α522 (15/25) or MC58 (47/59) upon transition from saliva to blood are shaded in *green* and *orange*, respectively. Only 21% of the genes in the *left* subnetwork consisting mainly of genes that were upregulated in α522 code for proteins involved in (energy) metabolism compared to over 66% of the genes that were upregulated in MC58 upon transition from saliva to blood. For each gene, the respective expression differences between conditions and strains, respectively, are color coded and indicated in each panel. White boxes indicate genes that were not differently expressed but are part of a subnetwork as identified by the integrative network analysis. Pie charts next to the sub-networks in each panel show the distribution of proteins in the respective subnetwork over the different COG functional classes

In order to identify differentially expressed functional subnetworks, we combined the transcriptomic with protein-protein interaction (PPI) network data of strain MC58 as deposited in the STRING database and used an algorithm which optimally identifies responsive subnetworks [53, 54]. This integrative network analysis revealed a densly connected subnetwork comprising mainly genes that are highly expressed in strain MC58 in blood (30/35 genes, 2-sample test for equality of proportions, *p* < 10^-5^) involved particularly in energy and carbohydrate metabolism (Fig. 3a). This subnetwork comprised genes of the tricarboxylic acid (TCA) cycle (*aceF*, *sdhC*, *sucA*, *sucC*, sucD*),* for the metabolism of pyruvate (*accB*), glycine (*gcvH*, *gcvT*), leucine (*leuA*, *leuB*) and fatty acids (*accB*, *acp*-2, *fabD*, *fabH*). The second subnetwork contained significantly more genes highly expressed in strain α522 (62/128 genes, *p* < 10^-4^) coding for a significantly different array of cellular functions (Pearson’s *χ* ^2^ test, *p* < 10^-6^). It comprised genes involved in the biosynthesis of the capsule (*siaA*/*synX*, *siaB*), peptidoglycan (*murB*, *murD*, *murE*), and LOS (*kdtA*, *lpxB*), respectively, as well as genes for trafficking, secretion and vesicular transport (*dprA*, *ffh*) or information storage and processing including in particular translation and ribosomal biogenesis genes (*rpmE*, *map*). A few genes in this second subnetwork were highly expressed in MC58 yet and included genes for glycolysis (*eno*, *galM*, *gapA*-2, *glk*, *pgm*), the genes for the Na^+^-translocating NADH-quinone reductase subunit B, D and F (*nqrB*, *nqrD*, *nqrF*) along with virulence-associated genes mentioned above like *dsbA-*1, *pilG*, *pilT*-2, *sodC* (Table 3). Transcriptomic differences in both strains in blood were thus organized in two subnetworks consisting mainly of genes that were either highly expressed in α522 or in MC58 and that coded for different biological functions.

**Table 3:** Differentially expressed genes coding for putative virulence genes and genes involved in meningococcal host interactions^(a)^

Locus	Gene	Product Name	Log2-fold expression level differences							
			in MC58 comparing		between MC58 and α522 in					
			Saliva vs. Blood^(b)^	Blood vs. CSF^(c)^	PPM+^(d)^	Saliva^(d)^	Blood^(d)^	CSF^(d)^	Blood vs. Saliva^(e)^	CSF vs. Blood^(f)^
Transcription										
NMB0594	*misS*	PhoQ-family sensor histidine kinase	n.s.^(g)^	n.s.	n.s.	n.s.	−2.50	n.s.	−2.00	2.51
NMB0595	*misR*	PhoP-family response regulator	−0.89	1.09	n.s.	n.s.	1.64	n.s.	−2.00	2.51
Capsule synthesis										
NMB0068	*siaC*	Capsule biosynthesis protein SiaC	2.07	−1.45	n.s.	n.s.	−1.67	−1.72	n.s.	n.s.
NMB0069	*siaB*	Capsule biosynthesis protein SiaB	2.33	n.s.	−1.66	−1.58	−2.73	−1.93	n.s.	n.s.
NMB0070	*siaA*	Capsule biosynthesis protein SiaA	2.85	n.s.	−1.63	−2.94	−2.85	−1.93	n.s.	n.s.
NMB0072	*ctrB*	Capsule export protein CtrB	2.28	n.s.	n.s.	n.s.	n.s.	−1.36	n.s.	n.s.
NMB0083	*lipB*	Capsule modification protein	n.s.	n.s.	n.s.	n.s.	−1.66	n.s.	n.s.	n.s.
LOS synthesis										
NMB0014	*kdtA*	3-Deoxy-D-manno-octulosonic-acid transferase	n.s.	n.s.	n.s.	n.s.	−2.83	n.s.	−2.84	2.58
NMB0017	*lpxC*	UDP-3-O-[3-hydroxymyristoyl] N-acetylglucosamine deacetylase	n.s.	n.s.	n.s.	n.s.	−1.24	n.s.	n.s.	n.s.
NMB0178	*lpxA*	UDP-N-acetylglucosamine acyltransferase	n.s.	n.s.	n.s.	n.s.	1.54	n.s.	n.s.	n.s.
NMB0180	*lpxD*	UDP-3-O-[3-hydroxymyristoyl] glucosamine N-acyltransferase	−1.40	n.s.	n.d.^(h)^	n.d.	n.d.	n.d.	n.d.	n.d.
NMB0199	*lpxB*	Lipid-A-disaccharide synthase	n.s.	n.s.	n.s.	n.s.	−2.92	n.s.	n.s.	n.s.
NMB1704	*lgtF*	Beta-1,4-glucosyltransferase	n.s.	n.s.	n.s.	n.s.	−2.82	n.s.	n.s.	n.s.
NMB1928	*lgtB*	Lacto-N-neotetraose biosynthesis glycosyl transferase LgtB	−1.27	n.s.	n.s.	n.s.	n.s.	n.s.	−1.56	n.s.
NMB2156	*rfaC*	Lipopolysaccharide heptosyltransferase I	2.20	n.s.	n.s.	n.s.	−1.03	n.s.	n.s.	n.s.
Pilus synthesis										
NMB0052	*pilT-1*	Twitching motility protein PilT	n.s.	n.s.	−0.93	n.s.	n.s.	n.s.	n.s.	n.s.
NMB0329	*pilF*	Type IV pilus assembly protein	−0.80	n.s.	n.s.	n.s.	n.s.	n.s.	n.s.	n.s.
NMB0333	*pilG*	Pilus assembly protein PilG	n.s.	0.98	n.s.	n.s.	2.86	n.s.	2.62	−2.32
NMB0768	*pilT-2*	Twitching motility protein PilT	1.36	n.s.	n.s.	n.s.	2.08	n.s.	2.18	−1.74
NMB1811	*pilP*	PilP protein	n.s.	−0.86	n.s.	n.s.	n.s.	n.s.	n.s.	n.s.
NMB1820	*pglB*	Pilin glycosylation protein PglB	−1.69	n.s.	n.s.	n.s.	−1.46	n.s.	n.s.	n.s.
NMB1821	*pglC*	Pilin glycosylation protein PglC	−1.73	n.s.	n.s.	n.s.	n.s.	n.s.	n.s.	n.s.
Efflux pumps										
NMB0318	*farA*	Fatty acid efflux system protein	−4.60	n.s.	n.s.	n.s.	−1.41	n.s.	−2.28	n.s.
NMB1714	*mtrE*	Multidrug efflux pump protein MtrE	−4.47	2.46	n.s.	n.s.	−2.85	n.s.	n.s.	n.s.
NMB1715	*mtrD*	Multiple transferable resistance system protein MtrD	−3.35	2.06	n.s.	n.s.	n.s.	n.s.	n.s.	n.s.
Adhesins and OMPs										
NMB0181	*-*	Putative outer membrane protein OmpH	−1.05	n.s.	n.s.	n.s.	n.s.	n.s.	n.s.	n.s.
NMB0182	*omp85*	Outer membrane protein OMP85	−0.98	n.s.	n.s.	n.s.	n.s.	n.s.	n.s.	n.s.
NMB0382	*rmpM*	Outer membrane protein class 4	n.s.	n.s.	n.s.	−0.87	n.s.	n.s.	n.s.	n.s.
NMB0497	*tpsA2*	Hemagglutinin/hemolysin-related protein	3.45	n.s.	n.d.	n.d.	n.d.	n.d.	n.d.	n.d.
NMB0663	*nspA*	Outer membrane protein NspA	1.46	−1.19	n.s.	n.s.	4.23	n.s.	3.92	−3.63
NMB1053	*opc*	Class 5 outer membrane protein OpcA	n.s.	n.s.	7.39	6.47	6.65	6.71	n.s.	n.s.
NMB1214	*tpsA3*	Hemagglutinin/hemolysin-related protein	n.s.	−2.00	n.s.	n.s.	3.62	n.s.	3.60	−3.74
NMB1946	*-*	Outer membrane lipoprotein	−0.92	n.s.	n.s.	0.78	1.53	0.78	n.s.	n.s.
NMB1969	*nalP*	Serine type autotransporter	n.s.	n.s.	0.88	1.64	1.05	1.08	n.s.	n.s.
Iron homeostatsis										
NMB0460	*tbp2*	Transferrin-binding protein B	1.84	n.s.	n.d.	n.d.	n.d.	n.d.	n.d.	n.d.
NMB0584	*-*	FrpC operon protein	n.s.	n.s.	n.s.	n.s.	1.20	n.s.	n.s.	n.s.
NMB0585	*-*	Putative iron-regulated protein FrpA	1.09	n.s.	n.d.	n.d.	n.d.	n.d.	n.d.	n.d.
NMB1206	*brfB*	Bacterioferritin B	n.s.	n.s.	n.s.	n.s.	2.18	n.s.	1.56	n.s.
NMB1207	*brfA*	Bacterioferritin A	n.s.	n.s.	n.s.	n.s.	2.16	n.s.	1.58	−1.39
NMB1540	*lbpA*	Lactoferrin-binding protein A	2.02	n.s.	n.s.	−1.56	n.s.	n.s.	2.16	n.s.
Stress response										
NMB0278	*dsbA-1*	Thiol:disulfide interchange protein DsbA	n.s.	n.s.	n.s.	n.s.	2.12	n.s.	1.94	−1.74
NMB0294	*dsbA-2*	Thiol:disulfide interchange protein DsbA	n.s.	1.38	n.s.	n.s.	n.s.	n.s.	n.s.	n.s.
NMB0587	*znuB*	ABC-type Mn2+/Zn2+ transporter, permease	n.s.	n.s.	n.s.	n.s.	−3.06	n.s.	−1.94	2.49
NMB0588	*znuC*	ABC-type Mn2+/Zn2+ transporer, ATPase	n.s.	n.s.	n.s.	−1.53	−2.26	n.s.	n.s.	n.s.
NMB1398	*sodC*	Superoxide dismutase	n.s.	n.s.	n.s.	n.s.	1.99	n.s.	2.28	−1.65
Others										
NMB0035	*-*	P47 lipoprotein	n.s.	n.s.	−1.15	n.s.	n.s.	n.s.	2.03	n.s.
NMB0065	*-*	Hypothetical protein NMB0065	4.24	n.s.	n.s.	n.s.	n.s.	n.s.	n.s.	n.s.
NMB0179	*fabZ*	(3R)-Hydroxymyristoyl-ACP dehydratase	−1.21	n.s.	n.d.	n.d.	n.d.	n.d.	n.d.	n.d.
NMB0543	*lctP*	L-Lactate permease	n.s.	n.s.	n.s.	n.s.	1.04	n.s.	1.39	n.s.
NMB0700	*iga*	IgA-specific serine endopeptidase	n.s.	−1.57	n.d.	n.d.	n.d.	n.d.	n.s.	n.s.
NMB0718	*hemH*	ferrochelatase	n.s.	n.s.	n.s.	n.s.	n.s.	n.s.	−1.37	n.s.
NMB0757	*purC*	phosphoribosylaminoimidazole-succinocarboxamide synthase	n.s.	n.s.	n.s.	n.s.	n.s.	n.s.	1.71	n.s.
NMB0790	*pgm*	Phosphoglucomutase	n.s.	n.s.	1.43	n.s.	2.52	1.76	2.52	n.s.
NMB0825	*-*	Putative ADP-heptose synthase	−1.11	n.s.	n.s.	n.s.	n.s.	n.s.	n.s.	n.s.
NMB0995	*-*	Macrophage infectivity potentiator-related protein	2.52	n.s.	n.s.	n.s.	n.s.	3.28	n.s.	3.06
NMB1332	*prc*	Carboxy-terminal peptidase	−0.90	n.s.	n.d.	n.d.	n.d.	n.d.	n.d.	n.d.
NMB1343	*-*	Hypothetical protein NMB1343	1.29	n.s.	n.s.	n.s.	1.50	n.s.	2.01	−1.85
NMB1436	*-*	Hypothetical protein	n.s.	n.s.	n.s.	n.s.	2.60	n.s.	2.81	−2.18
NMB1437	*-*	Hypothetical protein	−0.87	n.s.	n.s.	n.s.	n.s.	n.s.	n.s.	n.s.
NMB1438	*-*	Hypothetical protein	−1.26	n.s.	n.s.	n.s.	1.58	n.s.	1.87	n.s.
NMB1622	*norB*	Nitric oxide reductase	n.s.	n.s.	n.s.	n.s.	5.85	n.s.	5.40	n.s.
NMB1623	*aniA*	Copper-containing nitrite reductase	n.s.	n.s.	n.s.	n.s.	5.67	n.s.	5.55	−4.60
NMB1829	*-*	TonB-dependent receptor	−5.50	n.s.	n.s.	n.s.	n.s.	n.s.	n.s.	n.s.
NMB1840	*-*	Conserved membrane protein	n.s.	n.s.	n.s.	n.s.	1.78	n.s.	3.04	−2.20
NMB1898	*-*	Lipoprotein	−1.11	1.11	−1.17	n.s.	n.s.	n.s.	n.s.	n.s.
NMB1961	*-*	VacJ-related protein	n.s.	n.s.	n.s.	n.s.	−2.03	n.s.	−1.47	1.47

^(a)^ Virulence-associated genes were compiled from table 2 in ref. [21], table 1 in ref. [38], table 11.2 in ref. [39], and Additional file 1 from ref. [40] (see also Additional file 2 : S1). Only those genes are included in the transcriptome comparisons that showed significant expression differences in at least one type of comparison

^(b)^ Positive values indicate that the gene is expressed at higher levels in blood than in saliva

^(c)^ Positive values indicate that the gene is expressed at higher levels in CSF than in blood

^(d)^ Positive values indicate that the gene is expressed at higher levels in strain MC58 than in α522

^(e)^ Positive values indicate that the gene expression difference between saliva and blood is greater in strain MC58 than it is in α522

^(f)^ Positive values indicate that the gene expression difference between blood and CSF is greater in strain MC58 than it is in α522

^(g)^ n. s., not significant

^(h)^ n. d., no data due to missing α522 genome sequence data

Along with these cross-strain differences in the directionality of gene expression levels also cross-strain differences in the directionality of gene regulation between saliva and blood were organized in two functionally differing subnetworks (Pearson’s *χ* ^2^ test, *p* < 10^-6^) (Fig. 3b). The first subnetwork consisted almost exclusively of genes found to be upregulated in MC58 in blood compared to saliva (47/59 genes, *p* < 0.001), and over 70% of the genes in this subnetwork code for metabolic functions compared to only 24% in the other subnetwork (OR = 7.25, *p* < 0.001). It comprised genes for TCA cycle enzymes (*icd*, *lpdA1*, *sdhB*, *sucA*, *sucC*, *sucD*), for 2-oxocarboxylic acid metabolism (*asd*, *leuB*), oxidative phosphorylation (*petA*, *fixO*, *nuoA*), fatty acid metabolism (*accB*, *acp-2*, *fabG*, *fabH*), for thioredoxin-fold proteins (*kat*, NMB0946, NMB1366, *sodC*) required for oxidative stress response as well as the oxidoreductase genes *nqrB*, *nqrD*, *nqrF* and *aniA*, the latter playing a major role in the pathogenesis of IMD as described above. The second subnetwork consisted of genes that were strongly upregulated in strain α522 in blood compared to saliva (15/25 genes, *p* < 10^−7^) and included predominantly genes involved in information storage and processing and in particular for the translation machinery (*hisS*, *prmA*, *map*, NMB0347, NMB0348).

In line with the GSA results, integrative network analysis thus indicated that in contrast to strain α522 strain MC58 allocates transcriptional resources predominantly in the expression of metabolic genes in human blood.

### Elementary mode analysis of blood transcriptomic data indicate compensatory flux differences between both strains particularly in energy, glutamine and cysteine metabolism

In order to assess the possible impact of the transcriptomic differences on metabolism in blood in more detail, we reconstructed a condensed metabolic network based on the Nmb_iTM560 model for strain MC58 [55] comprising 123 enzymes (complexes) and 129 metabolites. The 54 elementary metabolic modes and pathways as identified by elementary mode analysis [56] are given in the Additional file 3: S2.

**Fig. 4:**
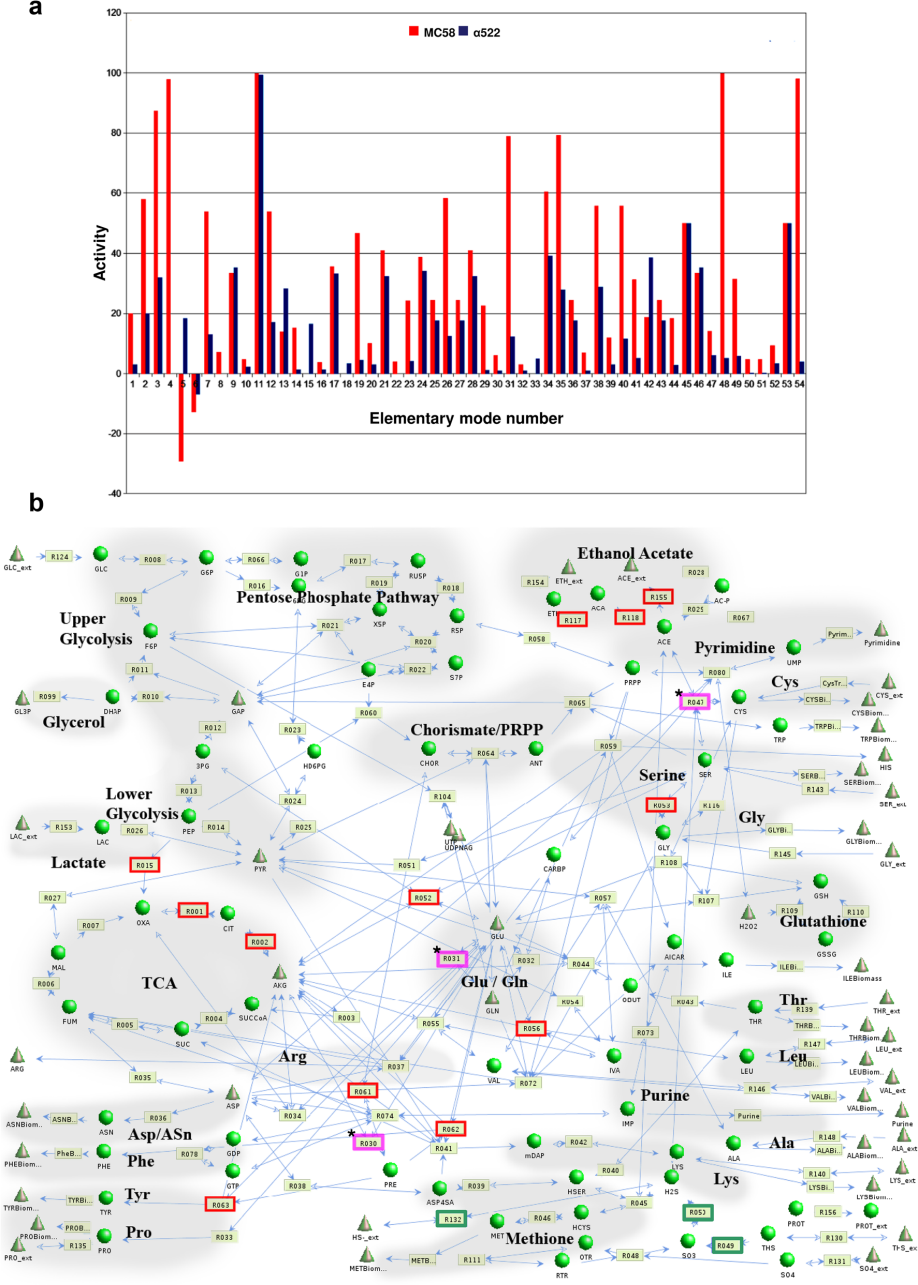
Analysis of gene expression data based on a metabolic model for strain MC58. **a** Comparison of elementary mode activities in MC58 and α522. The histogram depicts differences in the elementary mode activities (ordinate) for each of the elementary metabolic modes (abscissa) as defined in the Additional file 3: S2 for strain MC58 (*red*) and α522 (*blue*) based on gene expression data in human blood. **b** Inferred differences in metabolic fluxes between strain MC58 and strain α522 in blood based on a metabolic model for strain MC58. Internal metabolites which are considered to have balanced concentrations are given by *dark green* spheres, external metabolites which are allowed to accumulate or to be consumed by *green cones*, and reactions together with their corresponding numbers as *light green boxes*. The reactions for all reaction numbers are given in the Additional file 3: S2. *Arrows* connect reaction with metabolites. *Red* coloring indicates higher fluxes in MC58 compared to strain α522, whereas *blue* colouring indicates that the flux is slightly enhanced in α522. Asterisks along with pink colouring indicate that the reaction has an opposite direction in both strains

Computation of metabolic fluxes [57] based on the observed growth rates of both strains in human blood (Table 1), the corresponding gene expression data (Additional file 2: S1) and the average composition of human blood as taken from [58] showed that the flux activities in MC58 were 25-50% higher than in α522 with major differences in the metabolism of some amino acids (flux ratios higher than 6 or lower than 0.72) (Fig. 4a). Of note, one flux mode (EM 05) displayed an opposite direction between MC58 and α522, indicating that α522 uses a large amount of external glutamine (Gln) from human blood to produce gutamate (Glu).

For more detailed information about differences in important biochemical pathways, we converted the flux activities into enzyme and enzyme complex activities, respectively. The major differences are given in Table 4 and Fig. 4b. Only very few fluxes were slightly stronger in α522 such as R049, R050 and R132 which are all involved in sulfur metabolism. On the other hand, there were numerous reactions that were even more than tenfold stronger in MC58 than in α522 such as the acetate synthesis reactions R117, R118 and R155 suggesting that MC58 compared to α522 may accumulate acetate as intermediate metabolite in this environment. In meningococci, such metabolic stimulation has been shown to result from the consumption of lactate which in the presence of glucose is used as a source of additional energy [59]. Of note, lactate is a by-product of neutrophil glycolysis and enhances bacterial consumption of molecular oxygen, which depletes the substrate for neutrophil NADPH oxidase and thus blunts its oxidative burst [38]. Since both strains have almost identical growth rates in human blood (Table 1) MC58 probably requires this additional metabolic energy for other, not growth related processes such as, e.g., defense against the neutrophil oxidative burst. Other reactions that were particularly stronger in MC58 included R001, R002 and R015 leading from phosphoenolpyruvate (PEP) to α-ketoglutarate (AKG) as well as some reactions involved in amino acid metabolism like R052 and R053 resulting in the synthesis of serine (Ser) from Glu and glycine (Gly), respectively, or R056, R062 and R063 resulting in the synthesis of leucine (Leu), phenylalanine (Phe) and tyrosine (Tyr) from Glu, respectively. Since the reactions R052, R056, R062 and R063 are all transamination reactions resulting in the consumption of Glu and concomitant synthesis of AKG, these findings further suggest that MC58 and α522 might differ in their intracellular levels of Glu and AKG in human blood. Finally, reactions R030 and R031 have opposite directions in both strains in human blood, which indicates that α522 relies mostly on Gln to produce Glu with some Glu being further converted into AKG, whereas MC58 is capable to produce enough Glu from the TCA cycle and convert it further to Gln. Likewise, also reaction R047 which is the production of cysteine (Cys) from serine (Ser) has an opposite direction in both strains in human blood, and α522 consequently seems to rely on external Cys to produce Ser whereas MC58 is capable of producing Cys from Ser.

**Table 4:** Inferred reaction activity differences between MC58 and α522 in human blood based on gene cross-strain expression differences

Reaction	Chemical Equation	Enzyme(s)	Ratio^(a)^
R030	AKG + NADPH + NH_3_ = GLU + NADP	Glutamate dehydrogenase	−14.85
R031	ATP + GLU + NH_3_ = ADP + GLN	Glutamine synthetase	−3.03
R047	AcCoA + H2S + SER = ACE + CYS + CoA	Serine acetyltransferase + cysteine synthase	−2.32
R001	AcCoA + OXA = CIT + CoA	Citrate-synthase	13.86
R002	NADP + CIT = CO_2_ + NADPH + AKG	Aconitase	13.86
R015	CO_2_ + PEP = OXA	Phosphoenolpyruvate carboxylase	11.90
R052	3PG + GLU + NAD = AKG + NADH + SER	Serine synthesis: 3PG dehydrogenase + pserine aminotransferase + pserine phosphatase (SerA+ SerB + SerC)	7.63
R053	SER = GLY	Serine hydroxymethyltransferase	7.45
R056	AcCoA + GLU + IVA + NAD = AKG + CO_2_ + CoA + LEU + NADH	Leucine synthesis: isopropylmatate synthase + isopropylmalate dehydratase + isopropylmalate dehydrogenase + aminotransferase (LeuA + LeuB + LeuC + LeuD)	6.78
R061	CHOR = PRE	Chorismate mutase	12.58
R062	GLU + PRE = AKG + CO_2_ + PHE	Aminotransferase + phenyalanine synthesis	12.58
R063	GLU + NAD + PRE = AKG + CO_2_ + NADH + TYR	Aminotransferase + tyrosine synthesis	12.58
R117	ACA + NADH = ETH + NAD	Alcohol dehydrogenase	14.74
R118	ACE + NADH = ACA + NAD	Aldehyde dehydrogenase	14.74
R155	ACE = ACE_ext + H_ext	Acetate transporter	11.50
R049	O_2_ + THS = 2 SO_3_	Thiosulfate reductase	0.61
R050	3 NADPH + SO_3_ = H_2_S + 3 NADP	Sulfite reductase	0.71
R132	H_2_S = HS^−^_ext + H_ext	Sulfur transporter	0.45

^(a)^ Ratio of the reaction activity in MC58 divided by the reaction activity in α522. A ratio larger than one indicates that the reaction has a higher activity in MC58, and a negative ratio that the reaction occurs in opposite directions both strains

### Strain α522 differs from strain MC58 in Gln and Cys auxotrophy in vitro

**Fig. 5:**
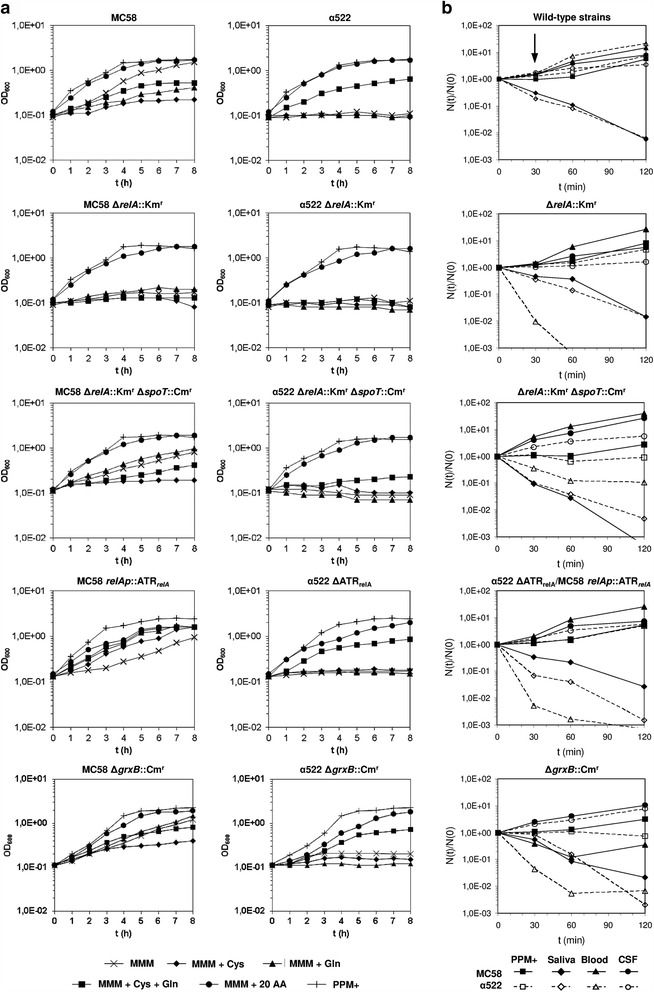
Growth phenotypes of *N. meningitidis* MC58 and α522 wild-type and mutant strains. **a** In vitro growth phenotypes. Growth as quantified by the optical density (OD_600nm_) is given on the ordinate and the time in hours on the abscissa. **b** Ex vivo growth phenotypes. The number of colony forming units for each time point (*N(t)*) relative to the initial number (*N(0)*) is given on the ordinate and the time in minutes on the abscissa. For each strain and condition the respective growth curves are coded as indicated in the insert in each panel, and the genotypes of the respective strains compared are shown along with the corresponding growth curves. In each experiment rich medium (PPM+) was used as growth control. The *arrow* at the top of panel **b** indicates the time when total RNA was extracted for microarray analysis

In order to experimentally validate the transcriptomic results with respect to possible differences in Gln and Cys metabolism, we assessed the growth of both strains in MMM supplemented with different amino acids as well as PPM+ (Fig. 5a). Whereas both strains were equally able to grow in PPM+ and MMM supplemented with all 20 proteinogenic amino acids at milimolar concentrations, strain α522 was not able to grow in MMM without amino acids. In addition to glucose or lactose as carbon source (data not shown) it requires Cys and Gln for growth (Additional file 1: Figure S4). Contrary to α522, the growth of MC58 was slightly suppressed by these two amino acids. The ability of MC58 but not α522 to grow in the absence of Cys and Gln indicates strain specific differences in the respective metabolic pathways, and the requirement of some meningococcal strains for Cys and its growth inhibiting effect on others has already been reported [60]. In parallel with these phenotypic differences, genome comparisons revealed also striking differences in Cys and Gln biosynthesis genes that might contribute to the phenotypic finding, although the repertoire of enzymes required for the biosynthesis of amino acids is otherwise highly conserved in both genomes. In addition to the duplication of *cysGHDNJI* genes in MC58 (Additional file 1: Figure S2A), these include large sequence differences in the phosphoadenosine phosphosulfate reductase CysH and the glutamate-ammonia-ligase adenylyltransferase GlnE which are among the least conserved genes involved in the biosynthesis of amino acids (Additional file 1: Figure S2C). CysH is required for the reduction of sulfate into hydrogen sulfide and thus for sulfur acquisition in *N. meningitidis* [61], and GlnE is a key regulatory enzyme in nitrogen assimilation in *E. coli* [62]. Furthermore, the intergenic region between *purL* and *glnB* differs in both strains due to the insertion of coding sequence in strain α522 upstream of *glnB* resulting in entirely different *glnB* promoter regions (Additional file 1: Figure S2D). Along with GlnE GlnB is involved in the regulation of nitrogen assimilation in *E. coli* via affecting the activity of GlnA, an enzyme that lies at the heart of the nitrogen assimilation network. GlnA is involved in the complex regulation of the interconversion of Glu to Gln in response to the intracellular concentration of ammonium, Glu, the Gln/AKG ratio, the redox (NADPH) and the free energy state of the cell [62]. Sequence variation at these loci is therefore likely to have pleiotropic effects, and a detailed experimental analysis of the biochemical consequences of these genetic differences is subject to ongoing work.

### Expression changes in numerous regulatory genes are likely compensatory

The large transcriptome differences observed particularly in blood could be caused by differences in regulatory elements acting either *in cis* or *in trans*, or be compensatory to ensure cellular homeostasis. Among the 1757 orthologous proteins those involved in gene expression regulation (COG K and T) were no more different between both strains than proteins involved in other functions (BSRP_COGK/T_ = 0.9944 vs. BSRP_other_ = 0.9936, Wilcoxon test, p > 0.05). Also the 200 bp upstream regions were not less conserved than the downstream orthologous genes (BSRN_5’-UTR_ = 0.9823 vs. BSRN_CDS_ = 0.9671, Wilcoxon test, p > 0.05). Of the 1450 single-copy orthologous genes used for transcriptomic comparisons 30 differed in the presence of putative mobile genetic elements within their 200 bp upstream regions (Additional file 2: S1). These comprised various so called minimal mobile elements [63, 64] as well Correia repeats and Correia repeat enclosed elements which have previously been shown to affect gene expression in a polar manner [65–67]. However, this class of genes was not enriched for genes differently expressed in at least one ex vivo condition. Although genetic differences in gene regulatory elements might contribute to the transcriptomic differences these data do not provide evidence yet that they are the sole reason of the large cross-strain expression differences observed particularly in human blood. They might rather orchestrate different compensatory gene expression adaptations in both strains in response to differences in the interaction of both strains with human blood components.

### Both strains activate different sets of regulatory genes in response to human blood

Of the 41 genes with significant expression differences in cross-condition or cross-strain comparisons involved in signal transduction or transcription (COG K or T), 18 showed significant expression level differences between both strains in blood (Table 5). The eight regulatory genes that were highly expressed in MC58 specifically in blood included in particular *cstA* annotated as carbon starvation protein A, NMB0398 coding for an ArsR family transcriptional regulator, *relA* encoding the guanosine 3'-(tri)diphosphate 5-'diphosphate ((p)ppGpp) synthetase of the stringent response pathway, as well as *misR* (NMB0595) coding for a PhoP-family response regulator of a two component signal transduction system. Of note, 64 genes of the 440 genes differently expressed in blood belong to the MisR regulon [33], and MisR was shown to be involved in the oxidative stress response in meningococci [34], required for colonization of host cells [68] and meningococcal survival in mice [69]. In addition, 22 differently expressed genes which are part of the Fur regulon [70] were almost all highly expressed in MC58. Since Fur senses cellular iron concentrations and since iron in general acts as a co-repressor, these data indicate that strain MC58 might experience more pronounced iron starvation in blood compared to strain α522. Likewise, another 45 genes are known to be regulated by FNR [32], the master regulator involved in the adaptation to oxygen-limited conditions, of which the majority (29) were also highly expressed in MC58.

**Table 5:** Differentially expressed genes involved in transcription and signal transduction (COG categories K and T)

Locus	Gene	Product	Log2-fold expression level differences							
			in MC58 comparing		between MC58 and α522 in					
			Saliva vs. Blood^(a)^	Blood vs. CSF^(b)^	PPM+^(c)^	Saliva^(c)^	Blood^(c)^	CSF^(c)^	Saliva vs. Blood^(a)^	Blood vs. CSF ^(b)^
Phospho transfer and signal transduction systems										
NMB0594	*misS*	Sensor kinase	n. s.^(d)^	n. s.	n. s.	n. s.	−2.50	n. s.	−2.00	2.51
NMB0595	*misR*	Response regulator	−0.89	1.09	n. s	n. s.	1.64	n. s.	1.30	n. s.
NMB0736	*ptsN*	Nitrogen regulator IIA	1.41	n. s.	n. a.^(e)^	n. a.	n. a.	n. a.	n. a.	n. a.
NMB1267	*-*	Tyrosine-phosphatase	−1.50	n. s.	n. s.	n. s.	−1.85	n. s.	n. s.	n. s.
NMB1250	*narP*	Response regulator	−1.57	n. s.	n. s.	n. s.	n. s.	n. s.	n. s.	n. s.
NMB1792	*basS*	Sensor histidine kinase	4.46	n. s.	n. s.	n. s.	n. s.	n. s.	n. s.	n. s.
HTH-type transcriptional regulators										
NMB0380	*fnr*	Crp/FNR family regulator	1.38	n. s.	n. s.	n. s.	n. s.	n. s.	n. s.	n. s.
NMB0398	*-*	ArsR-family regulator	2.34	n. s.	1.86	n. s.	2.09	n. s.	n. s.	n. s.
NMB0573	*-*	AsnC-family regulator	1.69	n. s.	n. s.	n. s.	n. s.	n. s.	n. s.	n. s.
NMB0810	*-*	TetR family regulator	1.42	n. s.	n. s.	n. s.	n. s.	n. s.	n. s.	n. s.
NMB0910	*-*	Putative phage regulator	1.73	n. s.	n. s.	n. s.	n. s.	n. s.	n. s.	n. s.
NMB1007	*-*	Putative phage regulator	2.29	n. s.	n. s.	n. s.	n. s.	n. s.	n. s.	n. s.
NMB1009	*-*	Putative phage regulator	3.19	−1.46	n. s.	n. s.	n. s.	n. s.	n. s.	n. s.
NMB1378	*-*	Iron-sulphur cluster-assembly repressor IscR	−1.67	n. s.	n. s.	n. s.	−1.82	n. s.	−2.07	1.77
NMB1563	*-*	GntR-family regulator	n. s.	1.09	n. d.^(f)^	n. d.	n. d.	n. d.	n. a.	n. a.
NMB1711	*-*	FadR-family regulator	−3.35	1.52	n. s.	n. s.	n. s.	n. s.	n. a.	n. a.
NMB1891	*-*	Putative phage regulator	−1.07	n. s.	n. s.	n. s.	−1.37	n. s.	−2.29	1.46
NMB2075	*-*	Bifunctional biotin-[acetyl-CoA-carboxylase] ligase/pantothenate kinase	n. s.	n. s.	n. s.	n. s.	−1.34	n. s.	−1.87	n. s.
Alternative sigma factors										
NMB0712	*rpoH*	Alternative sigma factor σ^H^	−2.56	1.22	n. s.	n. s.	n. s.	n. s.	n. s.	n. s.
NMB2144	*rpoE*	Alternative sigma factor σ^E^	n. s.	n. s.	n. s.	−1.48	−3.25	n. s.	n. s.	2.65
Others factors involved in gene regulation and stress response										
NMB0009	*-*	BolA family protein	1.66	−0.99	n. s.	n. s.	n. s.	n. s.	n. s.	n. s.
NMB0056	*dksA*	DnaK suppressor protein	n. s.	n. s.	n. s.	n. s.	−1.44	n. s.	n. s.	n. s.
NMB0126	*nusG*	Antitermination factor NusG	n. s.	−0.99	n. s.	n. s.	1.01	n. s.	n. s.	−1.55
NMB0282	*-*	Exoribonuclease II/R	n. s.	n. s.	n. s.	n. s.	−3.57	n. s.	n. s.	n. s.
NMB0577	*-*	Truncated NosR-like protein	−2.24	1.68	n. s.	n. s.	n. s.	n. s.	n. a.	n. a.
NMB0617	*rho*	Termination factor Rho	−1.57	n. s.	n. s.	n. s.	n. s.	n. s.	n. a.	n. a.
NMB0686	*rnc*	Endoribonuclease III	−1.68	n. s.	n. s.	n. s.	n. s.	n. s.	n. a.	n. a.
NMB0787	*-*	Periplasmic amino acid-binding protein	1.93	−1.47	n. s.	n. s.	n. s.	n. s.	n. s.	n. s.
NMB1336	*-*	Hypothetical protein	n. s.	1.33	n. s.	n. s.	n. s.	n. s.	n. s.	n. s.
NMB1368	*-*	Putative RNA helicase	n. s.	n. s.	n. s.	1.27	n. s.	n. s.	−2.27	n. s.
NMB1430	*greA*	Elongation factor GreA	1.18	n. s.	n. s.	n. s.	−1.65	n. s.	n. s.	n. s.
NMB1493	*cstA*	Carbon starvation protein A	n. s.	n. s.	n. s.	n. s.	3.42	n. s.	4.10	−3.44
NMB1500	*-*	Hypothetical protein	n. s.	−0.81	n. s.	n. s.	1.83	n. s.	2.18	−1.71
NMB1642	*nusA*	Termination factor NusA	n. s.	n. s.	n. s.	n. s.	−2.21	n. s.	−2.17	1.66
NMB1653	*-*	Hypothetical protein	1.29	−1.22	n. s.	n. s.	2.17	n. s.	n. s.	−2.25
NMB1660	*rpoZ*	RNAP omega chain	1.59	n. s.	n. s.	n. s.	n. s.	n. s.	n. s.	n. s.
NMB1735	*relA*	GTP pyrophosphokinase	n. s.	n. s.	n. s.	n. s.	1.82	n. s.	n. s.	n. s.
NMB1886	*-*	Hypothetical protein	−1.23	n. s.	n. s.	n. s.	n. s.	n. s.	n. s.	n. s.
NMB1944	*-*	ParB family protein	1.07	n. s.	n. s.	n. s.	n. s.	n. s.	n. a.	n. a.
NMB1981	*-*	S-ribosylhomocysteinase	−1.14	n. s.	n. a.	n. a.	n. a.	n. a.	n. a.	n. a.
NMB2037		Hypothetical protein	1.22	n. s.	n. s.	n. s.	1.48	n. s.	2.40	n. s.

^(a)^ Positive values indicate that the gene is highly expressed in blood

^(b)^ Positive values indicate that the gene is highly expressed in CSF

^(c)^ Positive values indicate that the gene is highly expressed in strain MC58

^(d)^ n. s. not significant

^(e)^ n. a. not applicable due to high sequence divergence between bot hhomologs

^(f)^ n. d. no data due to missing α522 genome sequence data

In turn, the ten regulatory genes highly expressed in α522 included NMB0282 encoding a exoribonuclease and *rpoE* (NMB2144) which codes for the alternative sigma factor E (σ^E^) and in *N. gonorrhoeae* is activated in response to oxidative stress [71]. Since phagocytic cells are a major source of reactive oxygen species and were present only in the blood assay, these data further suggests that σ^E^ might be involved in meningococcal interaction with human phagocytes. In line with previous findings that the expression of *aniA* and *norB* are under the negative control of σ^E^ [72], both strains also showed significantly different expression levels of *aniA* and *norB* especially in blood as mentioned above (Additional file 1: Figure S3). Along with the finding that nitric oxide (NO) generated by AniA inhibits platelet aggregation [51] this observation provides a direct link between the oxidative stress response and the pathophysiology of IMD. These data indicate that the complex gene expression differences between MC58 and α522 specifically in blood are caused by the activation of different sets of regulatory genes including MisR, Fur, FNR, RelA and σ^E^. In contrast to the expression of *rpoE* [72] and *misR* [33, 34] which in both cases was found to be auto regulated, Fur and FNR did not differ in their expression between both strains, suggesting strain-dependent differences in the post-transcriptional activation of these regulators especially in blood.

### Differences in promoter regions demonstrate an important role for the stringent response and Hfq mediated differences in gene expression regulation among both strains in blood

**Fig. 6:**
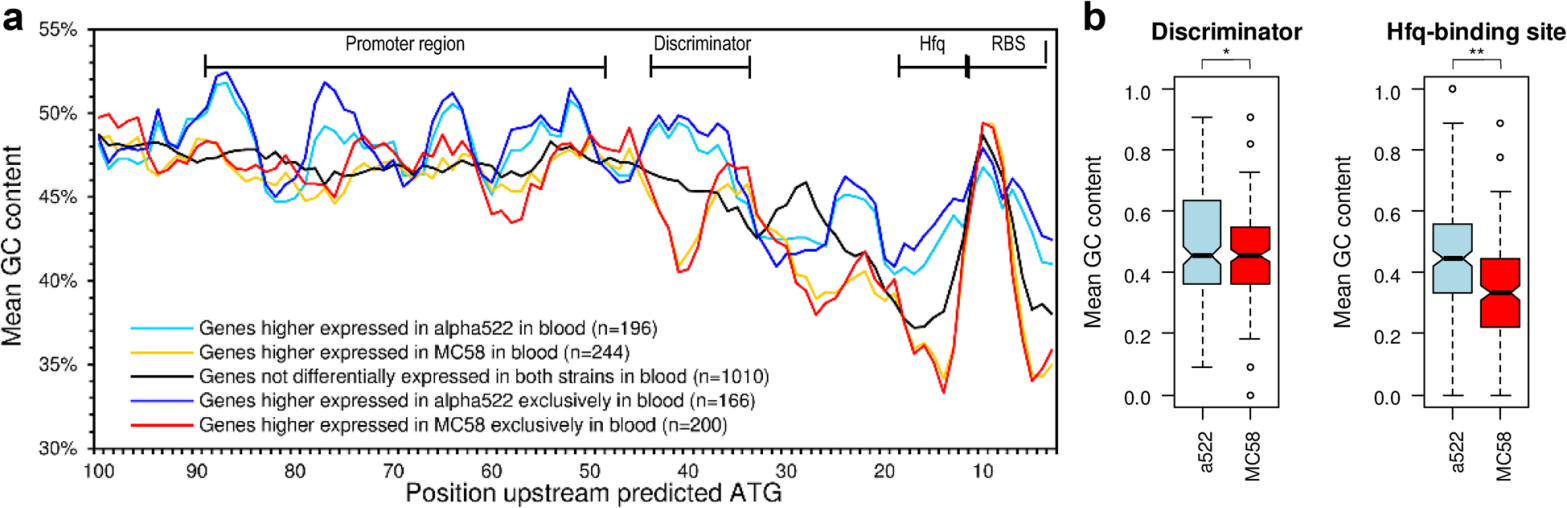
GC content variation in potential promoter regions based on the MC58 genome sequence. **a** Scatter plot of the GC content variation averaged over a 5-bp sliding window within 100 bp upstream regions for genes highly expressed in MC58 (*red* and *yellow lines*) or α522 (*light* and *dark blue lines*) in human blood. The *black line* gives the GC content of the respective upstream regions for genes not differently expressed. Regulatory regions are indicated at the *top* of the panel based on the average length of 5’-untranslated regions in *N. gonorrhoeae* [75]. The insert gives the number of genes in each gene set. **b** Box-and-whiskers plot depicting differences in the mean GC content of the putative discriminator (*left*) and Hfq-binding regions (*right*) between genes highly expressed in MC58 (*red*) or α522 (*blue*) in human blood as depicted in panel (**a**). The line within each box gives the median and the upper and lower margins the upper and the lower quartile, respectively. The whiskers denote the highest and the lowest values, respectively, and the open circles outliers. *: *p* < 0.05, **: *p* < 0.01 (Wilcoxon test)

Although sequence analyses of the 200 bp upstream regions of the 524 genes that were differently expressed between both strains in cross-strain comparisons failed to identify any consistent sequence differences or overrepresented *bona fide* TF binding sites for any of the cross-strain comparisons, the analysis of GC content variation yet revealed a 6 bp region immediately upstream of the predicted ribosome binding site (RBS) having a significantly lower GC content in genes that were expressed at higher levels in MC58 than in α522 in blood (GC_MC58_ = 37% vs. GC_α522_ = 42%, Wilcoxon test, *p* < 0.01) (Fig. 6). At the mRNA level, such AU-rich elements next to the RBS are often targets for the Hfq-mediated binding of small non-coding RNAs which thus post-transcriptionally regulate the degradation and/or translation efficiency of the corresponding mRNA [73]. In strain MC58, the RNA chaperone Hfq was already shown to be involved in the regulation of amino acid and energy metabolism, the oxidative stress response and required for survival in human blood [74]. The comparison of the differently expressed genes showed that of the 18 genes that are part of the Hfq regulon and that were included in this study, 9 were differently expressed in both strains, all higher in MC58. Therefore, these data suggest that Hfq contributes to gene regulation differences between both strains in blood.

Furthermore, genes differently expressed between both strains in blood had also significant GC content differences in a 10 bp region 40 bp upstream of the predicted RBS (GC_MC58_ = 44% vs. GC_α522_ = 48%, Wilcoxon test, *p* < 0.05) (Fig. 6), and there was a negative correlation between expression differences in blood and the deviation from the average genomic GC content (Spearman’s rank correlation ρ = - 0.12, p = 0.01). Since the average length of 5’-UTRs in *Neisseria* was shown to be between 40 and 50 bp [75] this region corresponds to the transcriptional start site. The length of this region, its pattern of GC content variation and its location close to the presumed transcriptional start site are hallmark features of so called discriminator regions [76] which in γ-proteobacteria determine whether the adjacent gene is activated or repressed during the stringent response. Accordingly, of the 440 genes differently expressed between both strains in human blood, 117 have a discriminator GC content higher than the genome-wide average of 50% and were highly expressed in α522, and 131 genes with a discriminator GC content lower than the average were highly expressed in MC58 (OR = 1.69, *p* < 0.01). Together, these genes account for 56% of all genes differently expressed in blood. Since activated targets typically have an AT-rich discriminator whereas repressed targets have a GC-rich discriminator, these data along with the higher expression of *relA* in MC58 indicate that in blood the stringent response pathway is comparatively more activated in MC58 than in α522. Genes highly expressed in MC58 and with a low GC discriminator region were predominantly involved in energy production and conversion (COG C, OR = 4.79, *p* < 0.05) and comprised genes involved in carbohydrate (*pykA*, *mapA*, *pgm*, *rpe*, *suhB*) and energy metabolism (*hprA*, *nuoA*, *nuoG*, *nuoL*, *nqrF*, *sdhC*, *lpdA1*, *pntA*, *leuB*, *fumC*, *fixO*, *aldA*, *etfA*), in aerobic energy generation (*sdhC, ccoN* (NMB1725), *ccoO* (NMB1724)), the genes for cytochrome c4 (NMB1805) and c5 (NMB1677)) as well genes required for the oxidative/nitrosative stress responses (*bfrAB*, *gltS*, *gshA*, *grx*, *sodC*) along with surface proteins like NspA, Lip and Laz. Genes highly expressed in α522 and having a high GC discriminator region were predominantly involved in cell envelope and outer membrane biosynthesis (COG M, OR = 11.7, *p* < 0.001) and included genes for LOS (*kdtA*, *lpxB*) and peptidoglycan biosynthesis (*ftsW*, *murD*, *murE*, *ddl*) as well as regulatory genes including *rpoE*.

### RelA and the stringent response pathway contribute to meningococcal ex vivo fitness in a condition and strain dependent manner

Although the stringent response pathway was already shown to be crucial for virulence in a number of bacterial pathogens [76], nothing is known about its contribution to meningococcal ex vivo or in vitro fitness so far. Therefore, the observation that *relA* was differently expressed between both strains exclusively in blood along with the finding that genes differently expressed between both strains in blood had significant GC content differences in their putative discriminator regions prompted us to further assess the contribution of the stringent response and in particulate of *relA* to meningococcal fitness ex vivo.

**Fig. 7:**
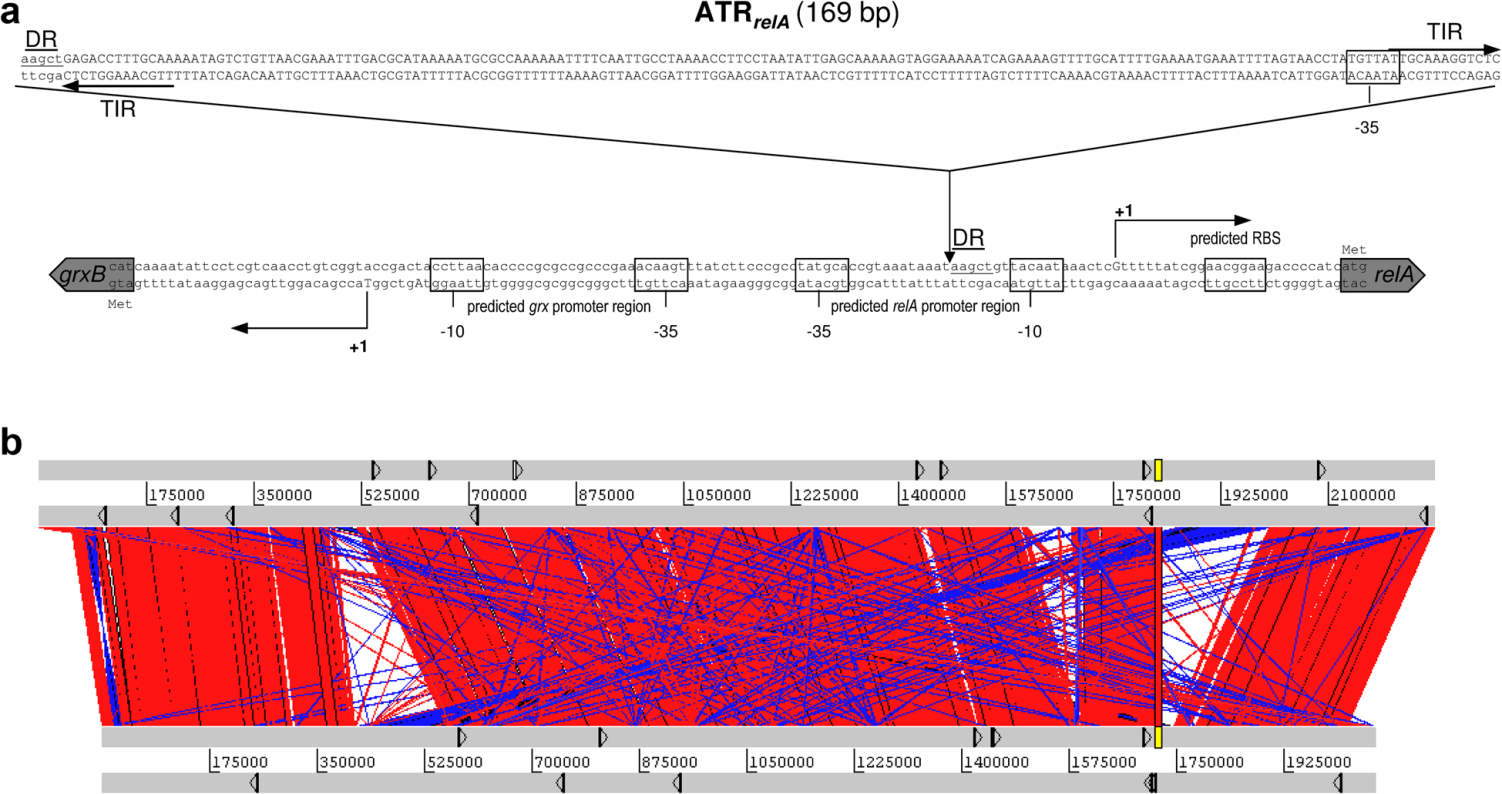
Genomic distribution of ATRs and the *relA* locus in *N. meningitidis*. **a** The intergenic region between *grxB* and *relA*. The integration site of a copy of an ATR repeat element upstream of *relA* (ATR_*relA*_) in strain α522 is indicated with respect to the MC58 locus. The transcriptional start sites as determined by 5’-RACE in both strains are indicated along with the deduced −35 and −10 boxes and the computationally predicted promoter regions using PPP [117]. DR: direct repeat. **b** Alignment of both the MC58 (*upper lane*) and α522 (*lower lane*) genomes as visualized with the Artemis comparison tool based on a BLASTN comparison. The linearized MC58 and α522 genomes are shown in the *upper* and *lower* panel as *gray bars*, and regions syntenic in both genomes are connected via *red* and inverted regions via *blue lines*, respectively. The location of ATRs is indicated by small *arrows* in each genome, and the *relA* region is highlighted in *yellow*

The machinery of the stringent response pathway comprises several enzymes involved in the turnover of (p)ppGpp which is a signaling nucleotide that coordinates a variety of cellular activities in response to changes in nutritional abundance [76]. In *E. coli*, RelA is activated upon amino acid starvation and together with SpoT is able to catalyze pyrophosphoryl transfer from ATP to GTP or GDP to synthesize (p)ppGpp. Together with DnaK suppressor (DksA), (p)ppGpp directs transcription initiation at particular gene promoters through binding to the interface between the two RNA polymerase subunits β’ and ω [77, 78]. In part, (p)ppGpp and DksA act by promoting the interaction of RNA polymerase with alternative σ-factors such as σ^E^ or σ^H^. When metabolic precursors are plentiful, SpoT instead degrades (p)ppGpp, and the vegetative σ-factor, σ^70^, directs RNA polymerase to genes that are crucial for bacterial replication. Whereas β’, ω, SpoT and DksA were identical in both strains they differed in the coding sequences and promoter region of RelA (Fig. 7a), and gene expression analyses via qRT-PCR further confirmed particular large and blood-specific cross-strain expression differences for *relA* but not for *spoT* or *dksA* (Additional file 1: Figure S3).

To test whether these differences in the coding sequences of *relA* affected its catalytic activity we assessed ppGpp levels in wild-type, ∆*relA*::Km^r^ as well as ∆*relA*::Km^r^ ∆*spoT*::Cm^r^ mutants in both strains during growth in PPM+ medium. As can be seen in Additional file 1: Figure S5A, both alleles were catalytically active and the catalytic activity was affected by the bacterial growth phase in a similar manner. Similar to the α522 wild-type strain, the MC58 ∆*relA*::Km^r^ mutant could further not grow in minimal medium, and in both genetic backgrounds the addition of all 20 proteinogenic amino acids could compensate for the loss of functional RelA but not the addition of only Cys and/or Gln to minimal medium (Fig. 5a). In addition, similar to *E. coli* (p)ppGpp^0^ strains the growth defect in minimal medium was less severe in meningococcal ∆*relA*::Km^r^ ∆*spoT*::Cm^r^ double mutants. In support of the notion that (too) high levels of (p)ppGpp might be toxic for the meningococcal cell we could not obtain viable isogenic *spoT* single deletion mutants, and a presumed *spoT* knock-out mutant had a compensatory frame-shift mutation prior the catalytic domain of *relA* resulting in reduced ppGpp levels (Additional file 1: Figure S5B and C).

With respect to ex vivo fitness the deletion of *relA* had no effect in MC58 on growth in saliva, blood or CSF whereas the α522 ∆*relA*::Km^r^ mutant was severely impaired exclusively in blood (Fig. 5b). The ex vivo fitness defect was less severe in a α522 ∆*relA*::Km^r^ ∆*spoT*::Cm^r^ double mutant. Unfortunately, since all attempts to clone full-length *relA* and *spoT*, respectively, in *N. meningitidis* for cis/trans complementation assays failed, the possibility that the observed phenotypes are, at least in part, caused by polar effects cannot be ruled out entirely. However, as depicted in Additional file 1: Figure S6, there are rho-independent transcriptional terminators at the 3′ ends of the *relA* as well as the *spoT* gene which have not been altered in the respective mutants. Furthermore, since the phenotype of the ATR_*relA*_ mutants particularly in blood resembles the phenotype of the *relA* mutants in both strains, and since both are also different from the phenotype of the *grxB* mutants, it seems rather unlikely that the *relA* phenotype is due to a polar effect on *grxB* expression (and vice versa) and not due to the decreased (p)ppGpp levels (Additional file 1: Figure S5).

Together, these data show that that the stringent response pathway is functional in both meningococcal strains despite their different relA alleles. The deletion of *relA* is conditional lethal and it is differently expressed between both strains under virulence-mimicking conditions in human blood. The effect of *relA* on ex vivo fitness is thus dependent on the environment and the genetic background.

### A non-coding mobile element affects meningococcal fitness in a strain- and condition-dependent manner

In addition to differences in the *relA* coding sequences, the intergenic region between *relA* and the upstream *grxB* encoding the redox enzyme glutaredoxin differed substantially in both strains due to the integration of an AT-rich (ATR) repeat element in strain α522 (Fig. 7a). ATR repeat elements occur 12 and 13 times in the genomes of α522 and MC58, respectively, of which only 10 have the same position in both genomes (Fig. 7b). They have a conserved length of 181 bp with ends forming a perfect 13–bp inverted repeat and belong to a class of non-autonomous DNA transposons also known as miniature inverted-repeat transposable-elements (MITEs) [79].

ATRs are almost exclusively located in intergenic regions in both genomes and are almost ten times more frequently found in intergenic regions flanked by convergently transcribed genes than expected by chance (Fisher’s exact test, OR = 9.64, *p* < 0.001). This indicates that the location in potential promoter regions might be under negative selection possibly due to adverse effects on the expression of neighboring genes. As experimentally determined by 5’-RACE, the −35 boxes of the *relA* promoter differ between both strains due to the insertion of ATR_*relA*_ in strain α522 between the −10 and −35 boxes (Fig. 7a). Furthermore, growth experiments demonstrated that the fitness of an ATR_*relA*_ knock-out strain was impaired only in α522 and only in human blood thus resembling the phenotype of the *relA* deletion mutant (Fig. 5b). In contrast to the deletion of ATR_*relA*_ or *relA*, the effect of deleting the neighboring *grxB* gene was condition- but not strain-dependent. These findings thus suggest that via affecting the expression of *relA*, differences in the binding of transcriptional regulators might contribute to the observed epistatic effects of ATR_*relA*_ on ex vivo fitness. The conditional essentiality of *grxB* for ex vivo fitness further supports the hypothesis that the oxidative stress response is required for blood survival especially in human blood. In support of a background- and condition-dependent effect of ATR_*relA*_ on meningococcal fitness, we could not observe any ex vivo fitness differences between the ATR_*relA*_ knock-in mutant strain MC58 *relA*p::ATR_*relA*_ and the corresponding MC58 wild-type strain.

Surprisingly, the integration of ATR_*relA*_ into the *relA* promoter region abolished however the inhibitory effect of Cys and Gln on growth in MMM in strain MC58, whereas the deletion of ATR_*relA*_ had no effect on the in vitro growth of α522 (Fig. 5a). This suggests a link between Cys and Gln metabolism and the stringent response regulation in a yet to define epistatic manner. Although the role of MITEs in meningococcal infection biology has already been established [66] this is the first time that a biological function has been shown for the ATR class of MITEs in an infection process.

## Discussion

It has recently been shown that transcriptional regulation in prokaryotes is more flexible than the genetic component of the organisms and that its complexity and structure plays an important role in phenotypic adaptation [11]. However, little is known so far about the significance of regulatory evolution that might underlie bacterial virulence. Accordingly, we used a hypothesis-generating systems biological approach [25, 26] to analyse gene-expression differences between two meningococcal strains from a hyperinvasive and a carriage clonal complex, respectively, under infection-mimicking conditions (Additional file 1: Figure S1).

**Fig. 8:**
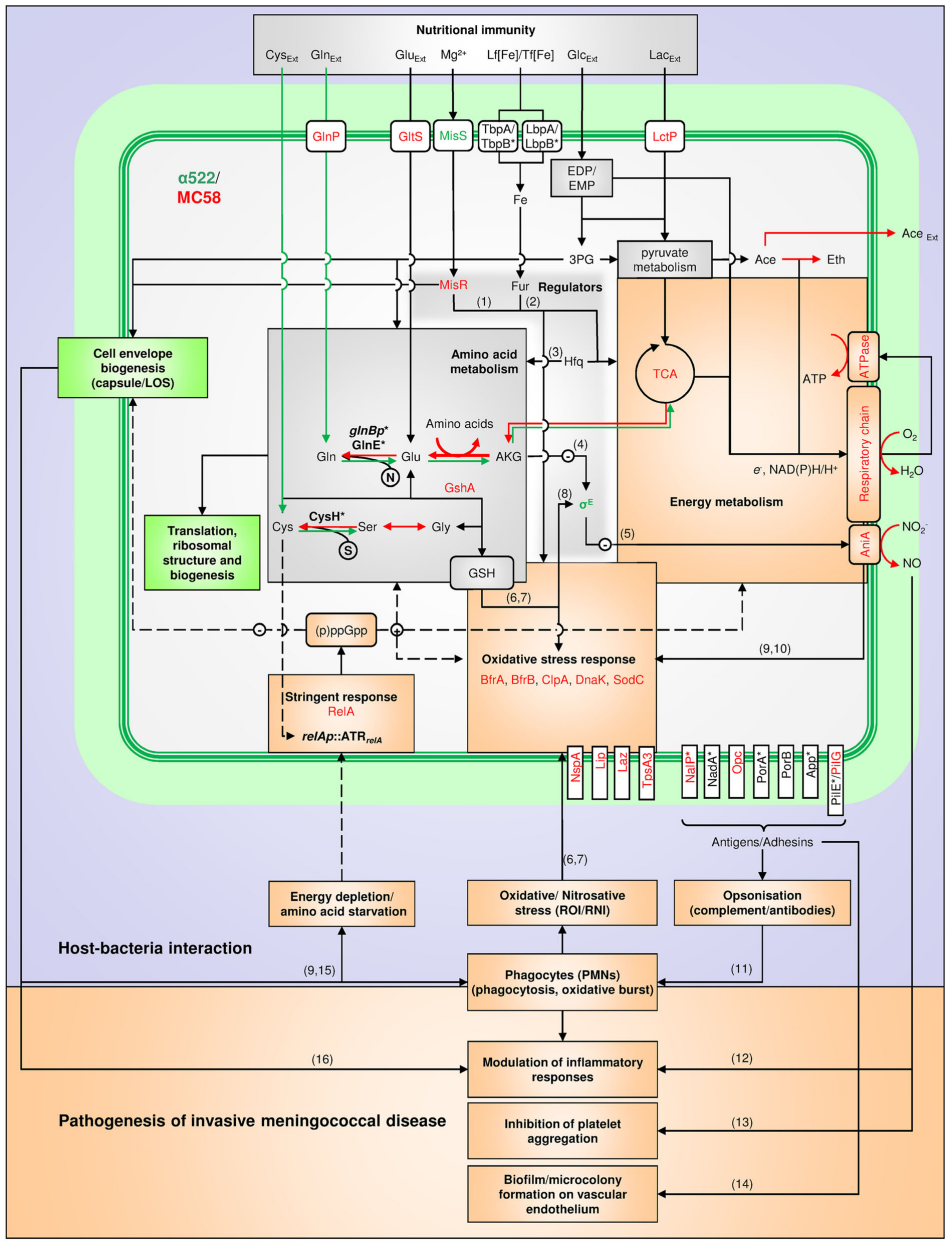
Graphical summary and hypothesis relating major findings of this work and published data. The figure is not intended to give a comprehensive overview of the entire metabolism and stress responses in *N. meningitidis* but to illustrate pathways that link metabolism, protein sequence and gene expression differences of selected (virulence-associated) genes and the pathogenesis of IMD as described in the main text. Accordingly, genes and pathways that were highly expressed in MC58 in blood and/or that are strongly upregulated between saliva and blood in MC58 are depicted in *red*, and genes and pathways that are highly expressed in α522 or that are strongly upregulated between saliva and blood in α522 are depicted in *green*. Asterisks next to enzyme or protein names indicate that the corresponding proteins have a less than average sequence similarity (BSRP < 0.958) or are entirely missing in strain α522. *Arrows* with plus signs indicate (predominantly) activating regulatory interactions, and *arrows* with minus signs (predominantly) inhibitory regulatory interactions. For further details and abbreviations see main text. The literature cited in the figure is indicated by bracketed numerals next to the respective arrows: (1) Newcombe et al. (2005) [33], (2) Delany et al. (2006) [70], (3) Fantappie et al. (2009) [74], (4) Monaco et al. (2006) [87], (5) Huis in’t Veld et al. (2011) [72], (6) Tala et al. (2011) [86], (7) Takahashi et al. (2015) [124], (8) Gunesekere et al. [71], (9) Criss and Seifert (2012) [38], (10) Seib et al. (2006) [84], (11) Schmitt et al. (2009) [83], (12) Stevanin et al. (2007) [52], (13) Kobsar et al. (2011) [51], (14) Coureuil et al. (2014) [125], (15) Virji (2009) [49], (16) Hellerud et al. (2015) [48]

Despite the substantial genetic differences between both strains affecting surface antigens as well as metabolic genes likely affecting Cys and Gln biosynthesis (summarized in Fig. 8), both were surprisingly similar in a variety of in vitro virulence assays and in their growth behavior under infection mimicking conditions (Table 1 and Fig. 5). In particular, the finding that both strains have the same fitness in human blood and CSF despite the large differences in the disease/carriage ratios between CC ST-32 and CC ST-35 strains further indicates that the ability to grow under infection mimicking conditions might be necessary but not sufficient for explaining the invasive property of certain meningococcal lineages. Virulence, i.e. host damage, might rather be related to the way how meningococci accomplish growth in this environment. In line with this hypothesis, the large transcriptome differences observed particularly in human blood (Fig. 1) indicate that different transcriptional programs probably compensate for the differences in the genetic backgrounds of both strains in response to host components. This so called phenotypic buffering is a general property of complex gene-regulatory networks [25, 36].

Upon transition from commensal to invasive behavior meningococci have to adapt to the accompanying large environmental changes caused largely by differences in nutritional and innate immunity in these different compartments. These comprise, e.g., differences in the presence of professional phagocytes or the availability and concentration and of key nutrients such as amino acids or iron [80–82]. Accordingly, it has already been shown that the oxidative burst, which is one of the most prominent effector mechanisms in human neutrophils, is modulated by neisserial porins, and – although to a lesser extent – also Opc has been described to be involved in *N. meningitidis* – neutrophil interaction (reviewed in [83]). The observed differences in the repertoire and/or expression of, e.g., Opc, PorA and PorB, could lead to differences in the phagocytic uptake by neutrophils and/or the activation of the oxidative burst (Fig. 8). Phagocytosis and the activation of the neutrophil oxidative burst in turn exert nutritional and oxidative/nitrosative stresses on the bacterial cell [38, 82]. The resulting damage to the bacterial cell triggers bacterial stress responses which in pathogenic bacteria have already been considered as virulence factors. In particular, lactate, a by-product of neutrophil glycolysis, was shown to enhance bacterial consumption of molecular oxygen in the presence of glucose, which depletes the substrate for neutrophil NADPH oxidase and thus blunts its oxidative burst [38, 59, 84]. Accordingly, our transcriptomic data analyses outlined above suggest a particularly strong metabolic activation in strain MC58 in blood (Figs. 1, 2, 3 and 4). Another important component in the oxidative/nitrosative stress responses in *Neisseria* is glutathione (GSH) [84]. It is synthesized from Cys, Gln/Glu and Gly which are either taken up from the environment or synthesized from precursors generated in the Entner–Doudoroff (ED) pathway and TCA cycle, respectively [85] (Fig. 8). Via the GSH cycle, the biosynthesis of Cys and Gln/Glu are thus linked to the oxidative/nitrosative stress response [86]. We therefore hypothesize that the observed sequence variations in metabolic genes such as CysH, GlnE or GlnB involved in Cys and Gln/Glu biosynthesis, respectively, lead to the activation of compensatory transcriptional programs to allow survival upon exposure to human blood phagocytes. Our data further indicate that *relA* contributes to the homeostatic transcriptional response to nutritional and oxidative/nitrosative stresses as it is required for amino acid biosynthesis in *N. meningitidis* (Fig. 5). Consequently, via variation in the GC content of the discriminator regions, the accumulation of (p)ppGpp during the stringent response might directly or indirectly contribute to the adapative regulation of genes required for energy metabolism, cell envelope biogenesis, translation and ribosome biogenesis and thus for large parts of the meningococcal transcriptome (Figs. 6 and 8). Furthermore, as the educt for Glu biosynthesis AKG provides a potential metabolic link between energy metabolism and the GSH cycle (Fig. 8) and was shown in *N. meningitidis* to contribute to, amongst others, the regulation of σ^E^ expression [87]. Since the expression of *aniA* was in turn shown to be under the negative control of σ^E^ [72], the finding that NO generated by AniA inhibits platelet aggregation [51] provides an exemplary link between oxidative stress, Glu/Gln metabolism and the pathophysiology of meningococcal disease. In this picture, virulence, i.e. host damage, occurs coincidentally due to the transcriptional compensation of metabolic deficiencies by including genes with an established role in the pathogenesis of IMD. In consequence, meningococcal virulence is a byproduct of global stress and metabolic responses potentially triggered by human neutrophils and is dependent on the genetic background of the invading strain. In line with the recently proposed damage-response framework of microbial pathogenesis [88], we further hypothesize that strain-dependent differences in the interaction of meningococci with human neutrophils have a central role in explaining meningococcal virulence differences. However, a better systems biological understanding of the interaction of human phagocytes with meningococcal cells under infection relevant conditions has yet to be reached, and in addition to transcription [89] many other layers such as post-transcriptional modifications, allosteric regulation [90] and non-coding small RNAs [91] are likely to play an important role in metabolic regulation in meningococci.

The phenotypic similarity of both strains under the ex vivo conditions tested further indicate that most of genotypic variation observed in the genomic comparisons (Additional file 1: Figure S2) is likely cryptic for selection under conditions encountered by the species during its commensal life-cycle, and only upon an environmental perturbation these genotypic differences do result in different phenotypes. In evolutionary genetics, this so called cryptic genetic variation (CGV) describes the part of the genetic diversity that has the potential to affect the phenotype but that is not expressed under the current genotypic or environmental conditions which limits the opportunities for selection to act on the variation [35, 36]. However, under atypical conditions, rare in the history of a population, CGV can generate phenotypic variation. Human blood is a condition that is normally not encountered by meningococci during its commensal life cycle, and since bacteria replicating in the bloodstream are not transmitted to new hosts it has not been encountered by any ancestral meningococcal strain before. CGV might thus contribute to the high standing genetic variation observed in meningococcal population since the conditions that induce such allelic effects are rare in the history of the population, and IMD would therefore result from the accidental unmasking of meningococcal CGV in human blood. Furthermore, CGV is a subclass of variation with conditional effects, either in form of G × G or in G × E interaction. Accordingly, not only by environmental perturbation discussed above but also by genetic perturbation such as deletion of *relA* we could experimentally uncover CGV likely in genes required for amino acid biosynthesis and ex vivo fitness (Fig. 5). Loci with such pleiotropic effects known as genetic hubs or buffers are a common feature of the genetics of gene expression [26] and their existence emerges from the concept of CGV [36]. These genes are important for buffering both environmental change and stochastic variation thus ensuring environmental and stochastic resilience. Rather surprisingly, there have been only very few verified genetic hubs in published genetical genomics studies to date. Based on our observations and the published data on the physiology of the stringent response in, e.g., *E. coli* we hypothesize that it contributes to the phenotypic buffering of CGV in metabolic genes and that RelA in particular might constitute such a regulatory hub coincidentally affecting also meningococcal virulence. Therefore, the elucidation of the genetic variability and molecular mechanisms of stress responses in *N. meningitidis* will be important for the understanding of virulence evolution in this commensal pathogen. Of note, whereas the variability in the complement and/or sequence of genes coding for surface antigens and metabolic functions, respectively, among different meningococcal lineages and their potential role in meningococcal virulence have already been well established (e.g. ref. [92–95]), the extent of sequence variability in stress response genes and their possible contribution to virulence differences in meningococci have not been addressed so far. In addition, differences in intergenic regions affecting gene expression regulation [15] are often overlooked genetic determinants in the search for bacterial virulence factors. The impaired fitness of the α522 ATR_*relA*_ deletion mutant in human blood (Fig. 5) provides an example of how regulatory evolution via the integration of non-coding MITEs [79] into promoter regions might contribute to fitness and consequently virulence differences among bacterial strains in a condition-dependent manner.

As all experimental approaches for studying meningococcal infection biology this study has also its limitations. First, the ex vivo conditions chosen might not be truly representative for the in vivo situation. In particular, although meningococci have been found to be part of the normal flora of the oral cavity [96] they actually colonize the human nasopharynx where they immerse in the liquid produced by nasopharyngeal epithelium and constantly interact with epithelial cells. Therefore, saliva produced by saliva glands may not be truly representative of this commensal environment. Second, given their high genetic diversity [2] more meningococcal strains need to be compared in order to see how generalizable the transcriptomic results are with respect to the entire species. Furthermore, any microarray-based approach restricts the number of genes being compared to those represented on the microarray. Comparative transcriptome sequencing (RNA-Seq) approaches circumvent this limitation and will allow not only for the detection of expression differences in non-coding small RNAs [97] but, due to the higher dynamic range compared to microarrays [98], also for the detection of differences in genes expressed at very low and very high levels, respectively. Finally, as it was experimentally not possible to generate *relA* and *spoT* complemented strains for *cis/trans* complementation assays, the phenotypes of the *relA* and *relA spoT* deletion strains could in principle be also affected by polar effects of the gene disruption on adjacent genes. This needs to be addressed in further experimental studies.

In summary, the data presented in this work allow novel hypotheses to be generated regarding the genetic basis of meningococcal virulence differences. They in particular warrant detailed analyses of the interaction between meningococci and human neutrophils, the physiological consequences of sequence differences in *cysH*, *glnE* or *glnBp*, the strain-dependent regulation of the stringent response in meningococci and how ATR might affect the expression of adjacent genes. The large transcriptomic data set provides per se an ex vivo gene expression compendium and as such a valuable resource for the meningococcal research community. Finally, our experimental approach further allows to challenge these findings in a larger panel of strains from carriage as well as hyperinvasive lineages and to seek for other genetic determinants affecting ex vivo fitness.

## Conclusion

In the present work, we showed that despite identical ex vivo phenotypes two genetically similar strains of *N. meningitidis* displayed large differences in their transcriptomes including numerous virulence genes and subject to environmental conditions. Consequently, the often employed “model strain” approach might give misleading results in genetically diverse species like *N. meningitidis* which might not be representative for the entire species. Furthermore, the finding that *relA* is conditionally essential and likely contributes to the transcriptional buffering of cryptic genetic variation in metabolic genes potentially limits the universality of RelA as a novel drug target [99]. Finally, beyond the variation in the set of virulence genes observed in “model” bacterial pathogens such as *E. coli*, the finding that a short, non-coding repeat element affected meningococcal fitness in a strain- and condition-dependent manner highlights the importance also of regulatory evolution in the emergence of virulence in commensal pathogens. Together, these findings demonstrate that “to generalize results across genetic backgrounds, experiments must be carried out across genetic backgrounds” [25], and because the pathogenic nature of a microbe is a quantitative trait resulting from multiple interacting loci, with allelic effects that are sensitive to the environmental conditions, best within a systems biological framework. In this respect our experimental approach is generic for the identification of loci that are associated with the invasive phenotype also in other genetically diverse commensal pathogens.

## Methods

### Strains and growth conditions

*N. meningitidis* serogroup B strain α522 (ST-35 CC) was isolated from a healthy carrier in the course of the Bavarian Carriage Study [100], whereas strain MC58 (ST-32 CC) (Research Resource Identifier (RRID): SCR_002200) has been isolated from a case of invasive disease [101] (Table 1). Meningococcal strains were routinely grown on Columbia blood agar (bioMérieux, Nürtingen, Germany). Proteose peptone medium supplemented with Polyvitex (bioMérieux, Nürtingen, Germany) (PPM+) and RPMI 1640 20 mM HEPES (Biochrom AG, Berlin, Germany) were used to prepare liquid cultures of the meningococcal strains. *E. coli* strain TOP10 cells (Invitrogen, Darmstadt, Germany) were routinely cultivated on Luria-Bertani (LB) agar or LB broth at 37 °C. Kanamycin and chloramphenicol were added when required to select for the deletion mutants and complementing meningococcal strains at a final concentration of 100 μg/ml and 7 μg/ml. Kanamycin and chloramphenicol were added at a final concentration of 30 μg/ml to select *E. coli* TOP10 strains during the cloning steps.

### In vitro growth experiments

For the in vitro growth assays, strains were grown overnight at 37 °C and in 5% CO_2_ on Columbia blood agar (bioMérieux). The next day, bacterial cells were inoculated in PPM+, meningococcal minimal medium (MMM, adapted from refs. [85, 102]) or MMM supplemented with 2.10 mM L-cysteine and further amino acids (Additional file 1: Figure S4) and grown at 37 °C with shaking at 200 rpm on a laborytory shaker Certomat ® H (Braun Melsungen AG, Melsungen, Germany) for 1 h. Bacterial cells were then adjusted to an optical density at 600 nm (OD_600_) of 0.1 using the WPA Biowave CO8000 Cell Density Meter (Biochrom Ltd., Cambridge, UK) and further cultivated at 37 °C and 200 rpm, and for each time point growth measurements were repeated three times.

### Adhesion and invasion assays

Cell adhesion and invasion assays were performed with human FaDu (ATCC® number HTB-43™, RRID: CVCL_1218) and Detroit 562 (CLS number 300399/p754_Detroit-562, RRID: CVCL_1171) nasopharyngeal epithelial cell lines. The multiplicity of infection (MOI) was adjusted to 10 in RPMI 1640 and the cells were infected for 6 h at 37 °C with 5% CO_2_. The numbers of adherent and intracellular bacteria were then assessed as described in [103]. All infection experiments were performed in duplicate and the experiments were repeated at least three times.

### Lipooligosaccharide typing and serum bactericidal assays

Lipooligosaccharide (LOS) immunotyping was performed using ELISA as described in [103]. Each serum bactericidal assay was repeated at least four times as described in [104].

### Sequencing and annotation of the *N. meningitidis* α522 genome

For *de novo* sequencing of the *N. meningitidis* α522 genome at 79-fold coverage, Roche/454 sequencing of 3-kb paired-end libraries using the GS FLX Titanium chemistry (Roche Diagnostics, Penzberg, Germany) was combined with Sanger sequencing of fosmid libraries generated with vector pCC1FOS (EPICENTRE Biotechnologies, Madison, WI) and of PCR products for gap closure on an ABI 3730XL sequencer (Applied Biosystems, Foster City, CA). *N. meningitidis* α522 scaffolds and unscaffolded contigs were arranged according to the genome of the reference strain *N. meningitidis* MC58 (GenBank accession AE002098) using the Mauve Contig Mover [105] and concatenated by 12-mer linkers (CTAGCTAGCTAG) containing stop codons in all six reading frames. The genome annotation system GenDB 2.0 [106] was used for automatic gene prediction and annotation of the α522 genome employing standard procedures. The final assembly of the α522 draft genome consisted of 21 contigs and comprised over 2.07 Mb with about 2000 predicted coding sequences (Table 1).

### Computational genome analyses

Genomes sequences were compared and visually inspected for manual data curation using Mauve [107] along with the Artemis Comparison Tool Release 5 [108] based on pairwise BLASTN alignments [109].

Orthologous genes were operationally defined as MC58 genes with a single significant best BLASTN hit in the α522 draft genome (E-value < 1/2000), corresponding to at least 80% sequence identity over at least 90% of the sequence length. For pairwise coding sequence comparisons BLASTP bit score ratios (BSRP) were computed from the bit scores (*S’*) using MC58 coding sequences as query and either the significant best hit coding sequences from α522 (best hit) or MC58 (self-hit) as subject sequences according to BSR = S’_best hit_/S’_self-hit_ as described in [110]. The encoded proteins were classified according to the COG functional classification scheme [37], and functional domains were assigned using NCBI’s Conserved Domain Database (CDD) [111]. We further used PSORTb 3.0 for the prediction of the subcellular localization of the encoded proteins [112].

Discriminative motif discovery was performed using MEME version 4.8.1 [113] to identify overrepresented *bona fide* TF binding sites within 200 bp regions upstream of predicted ATG translational start codons among differently expressed gene sets and with a minimum motif width of 2 bp and a maximum of 50 bp, respectively. Shuffleseq from the EMBOSS software package [114] was used for the randomization of 200 bp upstream sequences. ATR sequences together with the adjacent up- and downstream 10 bp were extracted from the original Z2491 genome annotation published in ref. [115]. Consecutively, the ATR consensus sequence obtained from a multiple sequence alignment with MUSCLE [116] was used for similarity searches in the genomes of MC58 and α522 with BLASTN [109], and promoter prediction in the *grxB* – *relA* intergenic region was carried using a neural network approach as implemented in PPP [117].

### Collection and processing of human specimens

Donors for saliva and blood were selected based on (a) not having a history of vaccination against *N. meningitidis* serogroups A, B, C, W and Y, respectively, (b) not having taken any antibiotics within 5 days prior sampling, and (c) not currently being carriers of meningococci. Human saliva was collected from the donors after stimulation with CRT paraffin (Ivoclar Vivadent GmbH, Ellwangen, Germany). The collected saliva was processed via centrifugation at 4000 rpm for 10 min followed by filter sterilization using 0.2 μm filters (Sarstedt, Nümbrecht, Germany) to eliminate all bacterial and eukaryotic host cells from the sample. Human CSF samples were obtained from the routine diagnostic laboratory at the Institute for Hygiene and Microbiology of the University of Würzburg and tested for sterility and the absence of antibiotics as well as leucocytes according to established standard operating procedures. The CSF and saliva samples were pooled and stored at −20 °C. Prior to the experiment, the pooled CSF samples were gassed with CO_2_ and pH was controlled to be in the physiologic range between pH 7.0 – 7.5. Heparinized human venous blood from four healthy donors (two males and two females) was drawn fresh on the day of the experiment and was used within an hour of collection.

### Ex vivo survival assays

Before exposure to human saliva, blood and cerebrospinal fluid bacterial strains were grown in PPM+ medium to mid log phase (OD_600nm_ ~ 0.5 – 0.6). One milliliter of the culture was harvested by centrifugation and after washing with 1x PBS, the bacterial pellet was resuspended in 1 ml of 1x PBS. Ten microliter of this suspension corresponding to ~ 10^6^ colony forming units (cfu)/ml were inoculated into 1 ml of human saliva, blood and cerebrospinal fluid, respectively, and incubated at 37 °C with shaking. Aliquots were taken out after 30 min, 60 min and 120 min and serial dilutions were plated out on Columbia blood agar (bioMérieux) to estimate the number of viable bacteria. Ex vivo infection experiments with human saliva and human CSF were performed using pooled saliva and pooled CSF samples, respectively, whereas ex vivo infections using whole venous blood were performed individually with each of the four blood samples and the isolated bacterial RNAs were pooled prior further analysis.

### Isolation of bacterial RNA

The meningococcal strains were grown in PPM+ medium to mid log phase (OD_600nm_ ~ 0.5 – 0.6), harvested by centrifugation and the bacterial pellets were resuspended in equal volumes of pooled saliva, pooled CSF and whole venous blood, respectively. To detect transcriptional differences in both strains independent of the exposure to human material PPM+ medium was used as control, and all samples were incubated at 37 °C with 5% CO_2_ for 30 min. Bacterial cells exposed to PPM+, pooled saliva and pooled CSF, respectively, were harvested by centrifugation at 4000 rpm for 10 min and shock frozen in liquid nitrogen for RNA isolation. Blood suspensions containing bacteria were centrifuged at 1000 rpm for 10 min to allow the blood cells to settle down, and the supernatant containing the bacterial cells were transferred to a fresh tube and harvested by centrifugation at 4000 rpm for 10 min. The pellets were then washed with erythrocyte lysis buffer (Qiagen, Hilden, Germany) and the bacterial pellet was shock frozen in liquid nitrogen for further RNA isolation. From the bacterial pellets, total RNA was isolated using TRIZOL® (Invitrogen GmbH, Darmstadt, Germany) according to the manufacturer’s protocol with slight modifications with respect to bacterial cell lysis. Contaminating DNA was removed by treating the samples with DNAse (Applied Biosystems) according to the manufacturer’s protocol. Absence of meningococcal chromosomal DNA was verified by PCR for the MLST housekeeping genes *fumC* and *adh*. Absence of contaminating host DNA was confirmed by PCR using primers Act-1 and Act-2 for the eukaryotic ß actin gene. Quality analysis of the RNA was performed using the Agilent 2100 Bioanalyzer (Agilent Technologies, Böblingen, Germany) and the RNA integrity factor was controlled to be > 7 in all cases as described by the manufacturer. All experiments were performed in triplicate to yield three RNA samples (replicates) for each strain under the four ex vivo conditions, and intact total RNA could be obtained for both strains and all conditions at 30 min of incubation (Additional file 1: Figure S7A and B).

### Microarray hybridization and data analysis

We used a spotted 70-mer oligonucleotide microarray comprising, amongst others, all 2063 open reading frames of *N. meningitidis* strain MC58 (GenBank AE002098). The layout of the microarray has been deposited in NCBI’s Gene Expression Omnibus database (http://www.ncbi.nlm.nih.gov/geo/) and is accessible through the GEO series accession number GPL9200. To allow for all possible pairwise comparisons of gene expression profiles we chose a common reference experimental design, and aliquots from each of the 24 RNA samples (isolated from the two strains under the four ex vivo conditions with three replicates each as described above) were pooled to form the common reference. Probe labeling, slide hybridization and analyses of raw data were performed as previously described [118] using a Tecan HS 4800 Pro hybridization station (Tecan Deutschland GmbH, Crailsheim, Germany) and a Genepix professional 4200A scanner (MDS Analytical Technologies, Ismaning, Germany) with the Genpix Pro 6.0 gridding software. Briefly, spots that were automatically reported as “Bad” or “Not found” by the Genepix Pro software were flagged and ignored during further processing. Likewise, spots that had a signal to noise ratio of < 3 were manually flagged as “Bad” and also ignored during subsequent analysis. The resulting raw data files were processed using the loess method for within slide normalization and the rquantile method for between slide normalization implemented in the R language [119] package Limma of the Bioconductor software project [120].

For further analyses, two data sets were generated both comprising 24 microarrays, one for the analysis of transcriptional changes only within strain MC58, and a second one for the comparison of the transcriptomes between strain MC58 and strain α522. The first dataset comprised almost the entire gene complement of strain MC58. However, due particularly to the presence of multiple, almost identical copies of the filamentous prophage Nf and a large duplication in the MC58 genome which both result in extensive within-genome cross homologies, it was not possible to design specific oligo probes for 76 genes. Consequently, to assess the transcriptional changes in strain MC58 only 1987 open reading frames were used and the resulting coverage is therefore 96.3% (1987/2063) with respect to the Genbank annotation AE002098. The second dataset was designed to specifically analyze transcriptional differences between both strains taking into account possible confounding effects due to sequence differences in orthologous genes and/or differences in the gene dosage between both strains. Both factors might affect the hybridization signal intensities of the respective oligo probes in microarray comparisons. Therefore, for transcriptome comparisons between both strains we selected only those genes and corresponding oligo probes, respectively, which met the following criteria: (i) all genes in MC58 used for transcriptome comparisons must have a single unique best BLASTN self-hit in the MC58 genome and a single unique best BLASTN hit in the α522 genome to exclude paralogous genes; (ii) the ratio between the BLASTN bit scores of their best hit in the α522 genome and of the respective MC58 gene self-hits must be greater than 0.6 to also exclude truncated CDSs; (iii) all oligo probes must have been found to hybridize with both genomes in a recently performed comparative genome hybridization study including both strains [28]; and (iv) all oligo probes must hit the orthologous genes in both genomes with fewer than eight mismatches over their entire length (>90% sequence identity). These criteria allowed us to examine the expression profile of 1450 genes between the two strains and in the four different ex vivo conditions using the limma package. We confirmed that there was no correlation between (absolute) gene expression level differences among strains and the percent sequence identity of the MC58 based oligonucleotides with their respective targets in the α522 draft genome (p_Pearson_ > 0.1, Additional file 1: Figure S7C), thus avoiding any strain bias in the expression data. Comparison of the expression levels of sixteen genes in PPM+, saliva, blood and CSF further revealed a good correlation between the microarray and the corresponding qRT-PCR data (*n* = 43 measurements, Person’s adjusted R^2^ = 0.74, p_Pearson_ < 10^−13^). All expression data are given in Additional file 2: S1.

### Assessment and analysis of significantly differently expressed genes

Using limma, only genes having a false discovery rate (FDR) < 0.05 after applying the Benjamini-Hochberg (BH) multiple testing correction and a log-odds (B-statistic) > 3, corresponding to a greater than 95% probability of being differentially expressed, were included in further data analyses. Overrepresentation analyses were performed using a contingency table and Fisher’s exact test to assess whether a COG functional category [37] was overrepresented among differently expressed genes. Unless stated otherwise, for all comparisons of multiple genes sets the Benjamini-Hochberg multiple testing correction was used with a FDR cut-off of 0.05.

### Metabolic reconstructions of strain MC58 and α522

The metabolic network of strain MC58 was reconstructed based on genes and reactions from the Nmb_iTM560 model [55], missing reactions were added according to references and KEGG database (*Neisseria meningitidis* MC58, serogroup B model). Futile cycles were eliminated and redundant reactions were removed from the collection, to derive a condensed network applicable for direct flux balance analysis (full simulation, not just sampling of modes). The generated model comprised 123 gene-associated enzymes and 129 metabolites involved in glycolysis, the pentose phosphate pathway and the TCA cycle. Furthermore, intermediary metabolism included lactate, acetate and acetoacetate metabolism, as well as amino acid metabolism, glutathione metabolism, purine and pyrimidine metabolism. Uptake transporters were taken into account if the metabolite appeared either in the composition list of human saliva or human blood as taken from ref. [58]. Network reconstruction was accomplished using the YANAsquare software [56]. A metabolic model for strain α522 was constructed in the same way, noting minor differences in metabolism (see results for details).

### Flux balance analysis of transcriptomic data

Flux computation was carried out by the YANAvergence package [57] and the Nmb_iTM560 metabolic model [55] modified as described above to get all the extreme pathways which describe the steady-state solution space of this genome-scale metabolic network. We used the normalized gene expression data described above to determine the corresponding enzyme activities encoded by genes that showed significant expression differences between both strains in saliva and blood, respectively. The activity of an enzyme complex comprised by different subunits was defined by the average intensity of the corresponding subunits, whereas the activity of a reaction catalyzed by isoenzymes was determined by the total activities of these encoding genes. Once convergence was reached, we used the estimated flux distribution to compute all the unmeasured enzyme activities. Finally, the enzyme activities computed according to our model were either compared between two conditions for strain MC58 or between both strains in human blood to investigate obvious differences.

### Protein-protein interaction network analysis of transcriptomic data

Protein-protein interaction network data for the core genome of *N. meningitidis* strain MC58 have been extracted from the STRING database (version 9.0, http://string-db.org) [54] yielding a total of 149,957 interactions between 2.052 genes. Based on the edge probabilities of the STRING database two microarray-specific networks of medium (probability > 0.4) and high confidence (probability > 0.7) have been derived. For the integrated network analysis the largest connected component of both networks have been used, comprising 21,071 interactions between 1,200 genes (medium confidence) and 4,360 interactions between 783 genes (high confidence). For all contrasts of interest *p*-values have been derived from the limma analyses. For the integrated network analysis node (gene) scores have been computed based on these *p*-values as detailed in [53] using the routines implemented in the R-package BioNet [121].

### Quantitative real-time RT-PCR

Validation of the microarray data was performed using the StepOnePlus^TM^ Real-Time PCR system with SYBR-Green (Applied Biosystems) as described in [122]. Briefly, 2 μg of DNA free RNA were reverse transcribed and suitable dilutions of the cDNA were used as template for quantification using the StepOnePlus™ Real-Time PCR system. The relative amounts of the cDNAs in the various samples were determined using the comparative CT method as described by the manufacturer. NMB1592 and *rpoC* which were not found to be differentially regulated under any of the conditions tested in this study by the microarray experiment were used as housekeeping genes for relative quantification of the investigated genes. All oligonucleotides used in this study are listed in Additional file 1: Table S2.

### Transcriptional start site mapping of *relA* with 5´ RACE

The transcriptional start site (TSS) of *relA* and *grx* in the strains MC58 and α522 was determined using the 5’/3’ RACE Kit, 2^nd^ Generation (Roche Applied Science, Manheim, Germany) according to manufacturer’s protocol. Briefly, cDNA was prepared using DNA free RNA from both the strains with a gene specific primer (relAGSP1 for *relA* and grxGSP1 for *grx*) and the reagents of the 5’/3’ RACE Kit. After incorporation of the poly(A) tail at the 5´end of the cDNA using the Terminal Transferase in the 5’/3’ RACE Kit, the tailed cDNA was amplified by PCR using the Oligo(dT) Anchor Primer along with primers relAGSP2 and grxGSP2 for *relA* and *grxB*, respectively (Additional file 1: Table S2). Both are nested primers which bind internal on the cDNA generated by relAGSP1 and grxGSP1 primer. After confirmation of the presence of a pure fragment of the expected size by agarose gel electrophoresis, the DNA fragment was purified using Qiagen PCR purification kit (Qiagen, Hilden, Germany) and sequenced on a ABI PRISM®3130 Genetic Analyzer (Applied Biosystems) using standard BigDye® Terminator v1.1 cycle sequencing chemistry (Applied Biosystems) to identify the transcriptional start site of the *relA* and *grxB* in both strains independently.

### Construction of isogenic deletion and insertion mutants

Deletion mutants were generated in the genetic background of *N. meningitidis* strain MC58 and α522 by replacing the entire encoding sequence with a kanamycin or a chloramphenicol resistance cassette (Additional file 1: Figure S6). Approximately 600 bp fragments of the flanking regions of the target genes were amplified by PCR from *N. meningitidis* MC58 and α522 genomic DNA, respectively. Primers used for generation of flanking regions (up- and downstream) of target genes are listed in Additional file 1: Table S2. Up- and downstream regions were created with different restriction sites. Flanking regions were amplified with Q5 high fidelity polymerase (NEB, Frankfurt, Germany), purified, digested and ligated into the pBluescript (pBS-KS, Stratagene, Heidelberg, Germany). Constructs were moved into *E. coli* TOP10 cells (Invitrogen) using chemical transformation technique. Deletion of the AT rich repeat region in the 5´region of *relA* in strain α522 was achieved by restriction free cloning using megaprime PCR. Around 600 bp regions upstream and downstream of the ATR in α522 were amplified using oligonucleotides where the 3´oligonucleotide of the upstream fragment had a complementarity of about 25 bp to the 5´region of the downstream fragment and similarly the 5´oligonucleotide of the downstream fragment had a complementarity of about 25 bp to the 3´region of the upstream fragment. These two purified fragments were further used as template for a fusion PCR to yield a fused DNA fragment containing the upstream and downstream regions of the ATR in strain α522. This fusion fragment was cloned into the pBluescript cloning vector, then transformed into strain α522 and deletion of the ATR in the mutant strain was confirmed by PCR and sequencing.

The insertion of the AT rich repeat region in the 5’ region of *relA* in strain MC58 was also achieved by restriction free cloning using megaprime PCR, as described above. Corresponding oligonucleotides were listed in Additional file 1: Table S2. The resulting PCR fusion fragment was then ligated into pBS-SK, cloned into strain MC58 and insertion of the ATR in the mutant strain was confirmed by PCR and sequencing.

The same method was used to create *grxB* deletion mutants in strain α522 and MC58 (Additional file 1: Figure S7A). Around 600 bp upstream and downstream of *grxB* were amplified, fused by megaprime PCR to a chloramphenicol resistance cassette and ligated into cloning vector pBS-SK. The resulting plasmid, listed in Additional file 1: Table S3, was then transformed into strain α522 and MC58.

All plasmids used to generate the deletion and insertion mutants are listed in Additional file 1: Table S3. Naturally competent MC58 and α522 cells were transformed and selected on GC agar plates containing kanamycin and/or chloramphenicol. The resulting mutants and meningococcal isolated were listed in Additional file 1: Table S4. The correct insertion of the resistance cassettes was confirmed by PCR and sequencing as well as by southern blot analysis using Hybond N+ nylon membranes (GE Healthcare, Munich, Germany) and the DIG DNA labeling and detection kits (Roche Applied Science, Mannheim, Germany) according to the manufacturer’s instructions.

### ppGpp extraction and quantification

Strains were grown over night at 37 °C and in 5% CO_2_ on blood agar plates. The next day, bacterial cells were preincubated in PPM+ medium at 37 °C and 200 rpm for 45 min, the optical density adjusted to OD_600nm_ = 0.1 and the bacteria cultured at 37 °C and 200 rpm in 50 ml of PPM+ medium. At OD_600nm_ values of 0.5 - 0.6 (mid log, ~ 1.5 h) and ≤ 1.6 (late log, ~ 4–5 h) the cells were harvested via centrifugation at 4 °C and 4000 rpm for 10 min in a Heraeus Megafuge 1.0 R (Thermo Scientific) centrifuge. The pellets were subsequently shock-frozen in liquid nitrogen and stored at −80 °C. For subsequent ppGpp extraction the pellets were resuspended in 1.25 ml of ice-cold 2 M formic acid and incubated on ice for 30 min. These samples were subsequently centrifuged at 4 °C at 4000 rpm for 10 min and the supernatant filtered through a 0.2 μm filter and stored at −20 °C until use.

For HPLC analyses, a Smartline HPLC system with a flow rate of 1.3 ml/min. The samples were loaded under initial conditions of 95% of solution A (Tris–HCl 20 mM, pH 8) and 5% of solution B (Tris–HCl 20 mM, sodium formiat, 1.5 M, pH 8) for 20 min. Then the solution B was ramped up to 60% during 45 min. The column was washed with 100% of solution B for 10 min and finally equilibrated with 95% of A and 5% B for 15 min. Quantification was performed using the ChromGate V3.3.2 software (Knauer, Berlin, Germany). The ppGpp standard was purchased from Trilink Biotechnologies. Standard curves were established using a total of ten different ppGpp concentrations ranging from 20 to 10000 pmol.

## Additional files

Additional file 1: Contains supplemental results and discussion describing the results of ex vivo cross-condition gene expression comparisons in strain MC58 along with the corresponding supplemental references and the figure legends to the supplemental **Figures S1 to S8** as well as the supplemental **Tables S1 to S4. Figure S1.** Experimental setup of the study. **Figure S2.** Comparison of the *N. meningitidis* α522 and MC58 genomes. **Figure S3.** qRT-PCR validation of ex vivo cross-strain expression differences in selected putative virulence-associated and regulatory genes. **Figure S4.** Growth of strain α522 in minimal medium supplemented with different combinations of amino acids. **Figure S5.** Comparison of the stringent response in *N. meningitidis* strain MC58 and α522. **Figure S6.** Genetic map of the *relA* and *spoT* loci in the mutant strains. **Figure S7.** Quality assessment of total RNA and microarray data. **Figure S8.** Discriminator regions in genes differently expressed in different ex vivo conditions in MC58. **Table S1.** Strain α522 specific genes. **Table S2.** Oligonucleotides used in this study. **Table S3.** Plasmids used in this study. **Table S4.** Strains used in this study. (ZIP 19627 kb)

Additional file 2: S1 is an Excel spread sheet containing the MC58 genome annotation data together with the results of the pairwise genome comparison with α522 and the microarray data. (XLSX 418 kb)

Additional file 3: S2 is an Excel spread sheet containing the results of the elementary mode analyses in human blood based on MC58 metabolic network model. (XLSX 18 kb)

## Abbreviations

(p)ppGpp: Guanosine pentaphosphate or tetraphosphate
3PG: 3-Phosphoglyceric acid
ACA: Acetaldehyde
AcCoA: Acetyl coenzyme A
ACE: Acetate
ADP: Adenosine diphosphate
AKG: α-ketoglutarate
ATP: Adenosine triphosphate
ATR: AT-rich
BH: Benjamini-Hochberg
bp: Base pair
BSRN: BLASTN bit score ratio
BSRP: BLASTP bit score ratio
CCs: Clonal complexes
CDD: Conserved domain database
CDS: Coding DNA sequence
CFU: Colony-forming units
CGV: Cryptic genetic variation
CHOR: Chorismate
CI: Confidence interval
CIT: Citrate
CO_2_: Carbon dioxide
CoA: Coenzyme A
COG: Cluster of orthologous groups
CSF: Cerebrospinal fluid
Cys/CYS: Cysteine
ED: Entner–Doudoroff
EM: Elementary mode
ETH: Ethanol
ext: External
FDR: False discovery rate
G x E: Gene-environment interaction
G x G: Gene-gene interaction
GC content: Guanine-cytosine content
Gln/GLN: L-glutamine
Glu/GLU: L-glutamate
Gly/GLY: Glycine
GSA: Gene set enrichment analysis
GSH: Glutathione
H: Hydrogen
H_2_S: Hydrogen sulfide
HS^−^: Bisulfide
IMD: Invasive meningococcal disease
IVA: Oxoisovalerate
LB: Luria-Bertani
Leu/LEU: Leucine
LOS: Lipooligosaccharide
LOS: Lipooligosaccharide
MITEs: Miniature inverted-repeat transposable-elements
MLST: Multilocus sequence typing
MMM: Meningococcal minimal medium
MOI: Multiplicity of infection
N(0): Is the initial quantity, at time t = 0
N(t): Quantity at time t
N: Cell number
n: Number
NAD: Nicotinamide adenine dinucleotide
NADH: Reduced NAD
NADP: Nicotinamide adenine dinucleotide phosphate
NADPH: Reduced NADP
NH_3_: Ammonia
NO: Nitric oxide
O_2_: Oxygen
OD_600_: Optical density at 600 nm
OR: Odds ratio
OXA: Oxaloacetate
*p*: *p*-value
PC: Phylogenetic clade
PEP: Phosphoenolpyruvate
Phe/PHE: Phenylalanine
PPI: Protein-protein interaction
PPM+: Proteose peptone medium supplemented with Polyvitex
PRE: Prephenate
*R*^*2*^: Linear regression
RBS: Ribosome binding site
S’: Bit score
Ser/SER: Serine
SO_3_: Sulfur trioxide
STs: Sequence types
t: Time point
TCA: Tricarboxylic acid
TF: Transcription factors
THS: Thiosulfate
TSS: Transcriptional start site
Tyr/TYR: Tyrosine
