# Supplementary material for: Transcriptomic buffering of cryptic genetic variation contributes to meningococcal virulence
Source: BMC Genomics. 2017 Apr 7;18:282. doi: 10.1186/s12864-017-3616-7 (PMC5383966; doi:10.1186/s12864-017-3616-7)
Supplement: Supplementary file 1 — Contains supplemental results and discussion describing the results of ex vivo cross-condition gene expression comparisons in strain MC58 along with the corresponding supplemental references and the figure legends to the supplemental Figures S1 to S8 as well as the supplemental Tables S1 to S4. Figure S1. Experimental setup of the study. Figure S2. Comparison of the N. meningitidis α522 and MC58 genomes. Figure S3. qRT-PCR validation of ex vivo cross-strain expression differences in selected putative virulence-associated and regulatory genes. Figure S4. Growth of strain α522 in minimal medium supplemented with different combinations of amino acids. Figure S5. Comparison of the stringent response in N. meningitidis strain MC58 and α522. Figure S6. Genetic map of the relA and spoT loci in the mutant strains. Figure S7. Quality assessment of total RNA and microarray data. Figure S8. Discriminator regions in genes differently expressed in different ex vivo conditions in MC58. Table S1. Strain α522 specific genes. Table S2. Oligonucleotides used in this study. Table S3. Plasmids used in this study. Table S4. Strains used in this study. (ZIP 19627 kb) [file 12864_2017_3616_MOESM1_ESM.zip › Ampattu Kischkies Revised Supplemental Text.docx]

**Supplemental information**

Transcriptomic buffering of cryptic genetic variation contributes to meningococcal virulence

**Authors:**

Biju Joseph Ampattu^1*^ ([abijujoseph@gmail.com](mailto:abijujoseph@gmail.com)), Laura Hagmann^1*^ ([lkischkies@hygiene.uni-wuerzburg.de](mailto:lkischkies@hygiene.uni-wuerzburg.de)), Chunguang Liang^2^ ([liang@biozentrum.uni-wuerzburg.de](mailto:liang@biozentrum.uni-wuerzburg.de)), Marcus Dittrich^2,3^ ([marcus.dittrich@biozentrum.uni-wuerzburg.de](mailto:marcus.dittrich@biozentrum.uni-wuerzburg.de)), Andreas Schlüter^4^ ([aschluet@cebitec.uni-bielefeld.de](mailto:aschluet@cebitec.uni-bielefeld.de)), Jochen Blom^5^ ([jochen.blom@computational.bio.uni-giessen.de](mailto:jochen.blom@computational.bio.uni-giessen.de)), Elizaveta Krol^6^ ([elizaveta.krol@synmikro.uni-marburg.de](mailto:elizaveta.krol@synmikro.uni-marburg.de)), Alexander Goesmann^5^ ([alexander.goesmann@computational.bio.uni-giessen.de](mailto:alexander.goesmann@computational.bio.uni-giessen.de)), Anke Becker^6^ ([anke.becker@synmikro.uni-marburg.de](mailto:anke.becker@synmikro.uni-marburg.de)), Thomas Dandekar^2^ ([dandekar@biozentrum.uni-wuerzburg.de](mailto:dandekar@biozentrum.uni-wuerzburg.de)), Tobias Müller^2^ ([Tobias.Mueller@biozentrum.uni-wuerzburg.de](mailto:Tobias.Mueller@biozentrum.uni-wuerzburg.de)), Christoph Schoen^1#^ (cschoen@hygiene.uni-wuerzburg.de)

* Both authors contributed equally to this work.

^#^ Corresponding author

**Affiliations:**

^1^ Institute for Hygiene and Microbiology, Joseph-Schneider-Straße 2, University of Würzburg, 97080 Würzburg, Germany; ^2^ Department of Bioinformatics, Biocenter, University of Würzburg, Am Hubland, 97074 Würzburg, Germany; ^3^ Department of Human Genetics, Biocenter, University of Würzburg, Am Hubland, 97074 Würzburg, Germany; ^4^ Center for Biotechnology (CeBiTec), Bielefeld University, Universitätsstr. 27, 33615 Bielefeld, Germany; ^5^ Institute for Bioinformatics and Systems Biology, Justus Liebig University Gießen, Heinrich-Buff-Ring 58, 35392 Gießen, Germany; ^6^ LOEWE-Center for Synthetic Microbiology, Hans-Meerwein-Straße, 35032 Marburg, Germany

**This supplement contains:**

Supplemental Results and Discussion

Supplemental References

Supplemental Figure Legends S1 to S8

Supplemental Tables S1 to S4

**Supplemental Results and Discussion**

**Condition-dependent expression changes affect 24% of the MC58 transcriptome and are enriched for metabolic but not for putative virulence functions**

Of the 1987 genes assayed in the two *ex vivo* cross-condition comparisons in strain MC58, over 330 genes were differently expressed exclusively between saliva and blood, indicating that the transition from saliva to blood is accompanied by a larger reorganization of the meningococcal transcriptome than the transition from blood to CSF (n = 53) (Figure 1 and S2A).

Of the 135 virulence-associated genes included in this analysis (supplemental data file S1, compiled from ref. [1-3]), 34 and 12 were differently expressed between saliva and blood and between blood and CSF, respectively. These included the *mtrCDE* operon encoding a multidrug efflux pump, the gene for the IgA protease (*iga*), the capsule biosynthesis genes *siaABC* and the gene for the outer membrane vaccine antigen NspA which contribute to enhanced survival against complement attack [4-6], and a number of other adhesin genes like *tpsA2*/*tpsA3* coding for the hemagglutinin-related proteins [7] and type IV pilus biosynthesis genes (*pilF*, *pilT-2*, *pglB*, *pglC*). Probably as a result of the changing iron concentrations also a number of iron-activated or –repressed genes were differently expressed between blood and saliva such as *tbp2* and *lbpA* which encode the transferring-binding protein 2 and the lactoferrin binding protein A, respectively. Surprisingly, genes differently expressed *ex vivo* were not significantly enriched for virulence-associated functions (Fisher’s exact test, p = 0.212).

Although there was no spatial clustering of differentially expressed genes along the MC58 chromosome (Figure S2A) [8] functional analysis of the transcription data showed significantly affected functional classes of known pathways (Figure 2A). In particular, gene set enrichment analysis (GSA) revealed that genes differently expressed between saliva and blood were significantly enriched for genes coding for nucleotide transport and metabolism (COG F, OR = 2.7, FDR = 0.044) (*adk*, *carA*, *carB*, *dut*, *gmk*, *guaA*, *guaB*, *ndk*, *ppx*, *pyrB*, *pyrC*, *pyrE*, *pyrH* *pyrI*, *purL*, *purM*, *purN*, *thyA*) thereby affecting in particular the metabolism of arginine and proline as well as of alanine, aspartate and glutamate. Likewise, genes differently expressed between blood and CSF were slightly enriched for genes involved in posttranslational modification, protein turnover and chaperones (COG O, OR = 2.7, FDR = 0.103) (*aat*, *dsbA-2*, *dsbC*, *dnaK*, *grpE*, *htpX*, *lon*, *clpX*, *hslO*).

GSA further showed significant differences in the directionality of gene expression changes (Figure 2B). The 173 genes higher expressed in saliva than in blood were enriched for genes involved in stress resistance and protein turnover (COG O, OR = 7.7, FDR = 0.025) (*htrA*, *htpX*, *dnaK*, *grpE*, *hsp33*/*hslO*, *groES*, *clpA*, *clpB* and *clpX*) as well as cell envelope and outer membrane biogenesis (COG M, OR = 3.2, FDR = 0.020) (*lgtB*, *lpxD*, *murD*, *murE*, *murF* and *murG*). Together, these groups of differentially expressed genes are a signature of an envelope stress response [9] likely due to the accumulation of damaged and/or misfolded proteins [10] in response to the action of antibacterial agents present in saliva [11]. In addition, genes higher expressed in saliva were also significantly enriched for genes involved in energy production and conversion (COG C, OR = 5.6, FDR = 0.024) (*nuoM*, *nuoN*, *pntB*, *fumB*, *ldhA*, *aldA*, *gpsA*). Of the 256 genes that in turn were higher expressed in blood than in saliva almost half (46%) code for proteins with so far not defined function (COG X). Among the genes with annotated functions were the capsule biosynthesis genes *siaABC* as well as *nspA*, *tbp2* and *lbpA* mentioned above. A detailed analysis of the blood – saliva gene expression differences based on protein-protein interaction (PPI) networks taken from the STRING database for strain MC58 [12] further supported an intimate link between the regulation of expression modules involved in cell wall (e.g., *murADEF*) as well as capsule (e.g., *siaABC)* biosynthesis with the regulation of genes for metabolic (e.g., *ispH*, *uppS*, *lspA*) and ribosomal (e.g., *frr*, *rpll*, *rpmH*, *rpsJ*) proteins as well as a chaperone subnetwork (*htrA*, *dnaK*, *grpE*, *hsp33*/*hslO*, *clpX* (data not shown).

Based on the observed growth phenotypes (Figure 5 and Table 1) and the expression differences predominantly in metabolic genes (Figure 2), elementary mode analysis of the saliva-blood transcriptomic data using a metabolic network model of strain MC58 [13] showed that with the exception of the metabolism of glycine and phenylalanine which seems to be more critical for MC58 survival in saliva nearly all other metabolic fluxes were stronger in blood. These included in particular the metabolism of amino acids including glutamate/glutamine, cysteine, lysine, arginine and aspartate, as well as glycolysis and lactate metabolism, the pentose phosphate pathway, purine and pyrimidine metabolism, and the metabolism of glycerol.

In contrast to these pronounced gene expression and ensuing metabolic changes reflecting the transition from colonization to host invasion, the transcriptomic differences between growth in human blood and CSF which both mimic the invasive condition were comparatively minor. Among the 71 genes higher expressed in blood than in CSF were *tpsA3* and a gene for a L-lactate permease protein (NMB1712). Likewise, the 80 genes higher expressed in CSF than in blood included genes involved in stress resistance and protein turnover (COG O, OR = 11.2, FDR = 0.058) (*dnaK*, *grpE*, *hsp33*/*hslO*, *clpX, lon*, *dsbA-2*, *dsbC*), *mtrCDE* mentioned above as well as genes involved in cell wall biosynthesis (*murD*, *murE*, *murF*). This transcription pattern might be a response to the presence of antimicrobial compounds present in human CSF which is partially similar to the one in saliva as described above [14, 15].

Taken together, these data show that the transition from commensalism to virulence is accompanied by large regulatory changes in the transcription predominantly of metabolic and stress response genes and only to a lesser extent of previously described virulence-associated genes. However, in both cross-condition comparisons genes higher expressed in blood were strongly enriched for genes not included in any COG functional category (COG X, OR = 6.5, FDR < 0.001) which suggests that our knowledge about the genetic factors that are involved in meningococcal blood stream as well as CSF survival and therefore virulence is still very limited.

**The expression pattern of transcription factors reflects meningococcal stress responses which are regulated by the alternative sigma factor σ^H^**

These large and complex condition-dependent changes in gene expression patterns were paralleled by expression differences affecting over 30 genes involved in gene expression regulation (COG K or T) particularly between saliva and blood (supplemental data file S1). These included in particular *rpoH* which encodes the alternative sigma factor σ^H^ and which showed large expression differences in both cross-condition comparisons. Lacking a general stress response sigma factor (σ^S^), σ^H^ is along with σ^E^ the only functional alternative sigma factor in *N. meningitidis* and was shown to be essential in *N. gonorrhoeae* [16]. Among the few differentially expressed transcription factors with described function were FNR (NMB0380) and NarP (NMB1250) which are both required to adapt to oxygen-limited growth in *Neisseria* [17, 18], as well as MisR (NMB0595) which is involved in the oxidative stress response in meningococci [19] and like NarP required for colonization of host cells and meningococcal survival in infant rats and mice [20]. In line with the comparatively small transcriptional changes accompanying the transition from blood to CSF, only nine regulators were differently expressed between both conditions. These included in particular *misR* and *rpoH* as well as NMB0009 coding for a BolA family protein which in *E. coli* was shown to have a general role in bacterial stress response and biofilm formation [21, 22]. The TFs with the largest expression differences between saliva and blood like NMB1711 or NMB1792 as well as a number of phage-encoded TFs such as NMB1009 or NMB2012. However, the majority of genes differently expressed between saliva and blood (288/429) or between blood and CSF (92/151) were not part of any meningococcal regulon described so far.

In line with this complex pattern of differently expressed TFs, discriminative motif discovery [23] failed to identify any overrepresented *bona fide* TF binding sites within 200 bp upstream regions in sets of co-regulated genes. However, in both cross-condition comparisons a region between 60 bp and 85 bp up-stream of the predicted ATG translation start codon had a significantly lower GC content in genes higher expressed in blood than in saliva (Wilcoxon test, p < 10^‑8^, ) or in CSF (p < 10^‑5^) (Figure S8). Since the average length of 5’-UTRs in *Neisseria* was shown to be between 40 and 50 bp [24] this region corresponds to the predicted RNA polymerase binding region.

As described above, *rpoH* is differently expressed in both cross-condition comparisons and a substantial fraction of the condition-dependently expressed genes might thus be transcribed with the help of σ^H^. In line with this hypothesis, σ^H^ and chaperon-associated proteins like DnaK, GrpE, ClpX and Hsp33/HslO are part a of transcriptionally co-regulated module, suggesting that similar to *E. coli* misfolded proteins might titrate chaperones from σ^H^ and allowing active σ^H^ to increase in turn the synthesis of chaperones and proteases [25]. As all *ex vivo* comparisons were performed at the same temperature of 37°C the lack of a general stress response sigma factor and the essentiality of *rpoH* in *Neisseria* [16] together strongly suggest that σ^H^ is a general stress response sigma factor and not only a heat shock sigma factor and required for survival of *N. meningitidis* within the human host.

**Supplemental** **References**

1. Snyder LA, Saunders NJ: **The majority of genes in the pathogenic Neisseria species are present in non-pathogenic Neisseria lactamica, including those designated as virulence genes.** *BMC Genomics* 2006, **7:**128.

2. Schoen C, Claus H: **Neisseria meningitidis genome sequencing projects.** In *Handbook of Meningococcal Disease.* Edited by Frosch M, Maiden MC. Weinheim, Germany: Wiley-VCH; 2006: 77-97

3. Echenique-Rivera H, Muzzi A, Del Tordello E, Seib KL, Francois P, Rappuoli R, Pizza M, Serruto D: **Transcriptome analysis of Neisseria meningitidis in human whole blood and mutagenesis studies identify virulence factors involved in blood survival.** *PLoS Pathog* 2011, **7:**e1002027.

4. Lewis LA, Ngampasutadol J, Wallace R, Reid JEA, Vogel U, Ram S: **The Meningococcal Vaccine Candidate Neisserial Surface Protein A (NspA) Binds to Factor H and Enhances Meningococcal Resistance to Complement.** *PLoS Pathog* 2010, **6:**e1001027.

5. Dixon GL, Heyderman RS, Kotovicz K, Jack DL, Andersen SR, Vogel U, Frosch M, Klein N: **Endothelial adhesion molecule expression and its inhibition by recombinant bactericidal/permeability-increasing protein are influenced by the capsulation and lipooligosaccharide structure of Neisseria meningitidis.** *Infect Immun* 1999, **67:**5626-5633.

6. Vogel U, Frosch M: **Mechanisms of neisserial serum resistance.** *Mol Microbiol* 1999, **32:**1133-1139.

7. Tala A, Progida C, De Stefano M, Cogli L, Spinosa MR, Bucci C, Alifano P: **The HrpB-HrpA two-partner secretion system is essential for intracellular survival of Neisseria meningitidis.** *Cell Microbiol* 2008, **10:**2461-2482.

8. Slager J, Kjos M, Attaiech L, Veening JW: **Antibiotic-induced replication stress triggers bacterial competence by increasing gene dosage near the origin.** *Cell* 2014, **157:**395-406.

9. Ades SE, Hayden JD, Laubacher ME: **Envelope Stress.** In *Bacterial Stess Responses.* Edited by Storz G, Hengge R. Washington, D.C.: ASM Press; 2011: 115-131

10. Turgay K: **Role of Proteolysis and Chaperones in Stress Response and Regulation.** In *Bacterial Stress Responses.* Edited by Storz G, Hengge R. Washington, D.C.: ASM Press; 2011: 75-90

11. Schenkels LC, Veerman EC, Nieuw Amerongen AV: **Biochemical composition of human saliva in relation to other mucosal fluids.** *Crit Rev Oral Biol Med* 1995, **6:**161-175.

12. Franceschini A, Szklarczyk D, Frankild S, Kuhn M, Simonovic M, Roth A, Lin J, Minguez P, Bork P, von Mering C, Jensen LJ: **STRING v9.1: protein-protein interaction networks, with increased coverage and integration.** *Nucleic Acids Res* 2013, **41:**D808-815.

13. Mendum TA, Newcombe J, Mannan AA, Kierzek AM, McFadden J: **Interrogation of global mutagenesis data with a genome scale model of Neisseria meningitidis to assess gene fitness in vitro and in sera.** *Genome Biol* 2011, **12:**R127.

14. Brandenburg LO, Varoga D, Nicolaeva N, Leib SL, Wilms H, Podschun R, Wruck CJ, Schroder JM, Pufe T, Lucius R: **Role of glial cells in the functional expression of LL-37/rat cathelin-related antimicrobial peptide in meningitis.** *J Neuropathol Exp Neurol* 2008, **67:**1041-1054.

15. Metz-Boutigue MH, Kieffer AE, Goumon Y, Aunis D: **Innate immunity: involvement of new neuropeptides.** *Trends Microbiol* 2003, **11:**585-592.

16. Laskos L, Ryan CS, Fyfe JA, Davies JK: **The RpoH-mediated stress response in Neisseria gonorrhoeae is regulated at the level of activity.** *J Bacteriol* 2004, **186:**8443-8452.

17. Bartolini E, Frigimelica E, Giovinazzi S, Galli G, Shaik Y, Genco C, Welsch JA, Granoff DM, Grandi G, Grifantini R: **Role of FNR and FNR-regulated, sugar fermentation genes in Neisseria meningitidis infection.** *Mol Microbiol* 2006, **60:**963-972.

18. Lissenden S, Mohan S, Overton T, Regan T, Crooke H, Cardinale JA, Householder TC, Adams P, O'Conner CD, Clark VL, et al: **Identification of transcription activators that regulate gonococcal adaptation from aerobic to anaerobic or oxygen-limited growth.** *Mol Microbiol* 2000, **37:**839-855.

19. Tzeng YL, Kahler CM, Zhang X, Stephens DS: **MisR/MisS two-component regulon in Neisseria meningitidis.** *Infect Immun* 2008, **76:**704-716.

20. Jamet A, Rousseau C, Monfort JB, Frapy E, Nassif X, Martin P: **A two-component system is required for colonization of host cells by meningococcus.** *Microbiology* 2009, **155:**2288-2295.

21. Adnan M, Morton G, Singh J, Hadi S: **Contribution of rpoS and bolA genes in biofilm formation in Escherichia coli K-12 MG1655.** *Mol Cell Biochem* 2010, **342:**207-213.

22. Santos JM, Freire P, Vicente M, Arraiano CM: **The stationary-phase morphogene bolA from Escherichia coli is induced by stress during early stages of growth.** *Mol Microbiol* 1999, **32:**789-798.

23. Bailey TL, Elkan C: **Fitting a mixture model by expectation maximization to discover motifs in biopolymers.** In *Second International Conference on Intelligent Systems for Molecular Biology; Stanford University, Stanford, California, USA*. Edited by Altman RB, Brutlag DL, Karp PD, Lathrop RH, Searls DB. AAAI Press; 1994: 28-36.

24. Remmele CW, Xian Y, Albrecht M, Faulstich M, Fraunholz M, Heinrichs E, Dittrich MT, Muller T, Reinhardt R, Rudel T: **Transcriptional landscape and essential genes of Neisseria gonorrhoeae.** *Nucleic Acids Res* 2014, **42:**10579-10595.

25. Lim B, Gross CA: **Cellular Response to Heat Shock and Cold SShock.** In *Bacterial Stress Responses.* Edited by Storz G, Hengge R. Washington, D.C.: ASM Press; 2011: 93-114

26. Rutherford K, Parkhill J, Crook J, Horsnell T, Rice P, Rajandream MA, Barrell B: **Artemis: sequence visualization and annotation.** *Bioinformatics* 2000, **16:**944-945.

27. Baart G, Zomer B, de Haan A, van der Pol L, Beuvery EC, Tramper J, Martens D: **Modeling Neisseria meningitidis metabolism: from genome to metabolic fluxes.** *Genome Biology* 2007, **8:**R136.

28. Snyder LA, McGowan S, Rogers M, Duro E, O'Farrell E, Saunders NJ: **The repertoire of minimal mobile elements in the Neisseria species and evidence that these are involved in horizontal gene transfer in other bacteria.** *Mol Biol Evol* 2007, **24:**2802-2815.

29. Bentley SD, Vernikos GS, Snyder LA, Churcher C, Arrowsmith C, Chillingworth T, Cronin A, Davis PH, Holroyd NE, Jagels K, et al: **Meningococcal genetic variation mechanisms viewed through comparative analysis of serogroup C strain FAM18.** *PLoS Genet* 2007, **3:**e23.

30. Tettelin H, Saunders NJ, Heidelberg J, Jeffries AC, Nelson KE, Eisen JA, Ketchum KA, Hood DW, Peden JF, Dodson RJ, et al: **Complete genome sequence of Neisseria meningitidis serogroup B strain MC58.** *Science* 2000, **287:**1809-1815.

31. Kawai M, Uchiyama I, Kobayashi I: **Genome comparison in silico in Neisseria suggests integration of filamentous bacteriophages by their own transposase.** *DNA Res* 2005, **12:**389-401.

32. Morgan GJ, Hatfull GF, Casjens S, Hendrix RW: **Bacteriophage Mu genome sequence: analysis and comparison with Mu-like prophages in Haemophilus, Neisseria and Deinococcus.** *Journal of Molecular Biology* 2002, **317:**337-359.

33. van Ulsen P, Tommassen J: **Protein secretion and secreted proteins in pathogenic Neisseriaceae.** *FEMS Microbiol Rev* 2006, **30:**292-319.

34. Harrison OB, Claus H, Jiang Y, Bennett JS, Bratcher HB, Jolley KA, Corton C, Care R, Poolman JT, Zollinger WD, et al: **Description and nomenclature of Neisseria meningitidis capsule locus.** *Emerg Infect Dis* 2013, **19:**566-573.

**Supplemental Figures**

**Figure S1:** Experimental setup of the study.

For details see text.

**Figure S2:** Comparison of the *N. meningitidis* α522 and MC58 genome.

(A) Circular plot of the MC58 genome used as reference. The outermost circle shows larger contiguous regions missing in α522. IHT-B and –C denote the islands of horizontal transfer B and C, respectively. D indicates a genome duplication in MC58, RTX-1 the repeat-in-toxin region 1 encoding FrpA/C-like proteins, and “gap” an assembly gap in strain α522. The GC content plot is shown in the innermost circle. The other circles denote genes with expression differences either between conditions or strains as indicated in the insert. The picture was generated using DNA plotter from the Artemis software [26].

(B) Box-and-whiskers plot depicting differences in the BSRPs among the different COG functional categories. The line within each box gives the median and the upper and lower margins the upper and the lower quartile, respectively. The whiskers denote the highest and the lowest values, respectively, and the open circles outliers. The boxes are colored according to the different COG functional classes: blue, information storage and processing; green, cellular processes and signaling; red, metabolism; and grey, poorly characterized or not in COG.

Abbreviations: C, Energy production and conversion; D, Cell cycle control, mitosis and meiosis; E, Amino acid transport and metabolism; F, Nucleotide transport and metabolism; G, Carbohydrate transport and metabolism; H, Coenzyme transport and metabolism; I, Lipid transport and metabolism; J, Translation; K, Transcription; L, Replication, recombination and repair; M, Cell wall/membrane biogenesis; N, Cell motility; O, Posttranslational modification, protein turnover, chaperones; P, Inorganic ion transport and metabolism; Q, Secondary metabolites biosynthesis, transport and catabolism; R, General function prediction only; S, Function unknown; T, Signal transduction mechanisms; U, Intracellular trafficking and secretion; V, Defense mechanisms; X, Not in COGs.

(C) Box-and-whiskers plot depicting differences in the BSRPs among the different amino acid biosynthesis pathways as given in the supplementary data file S1.

(D) Comparison of the *glnB* locus containing, amongst others, *glnB* encoding the signal-transducing nitrogen regulatory protein PII and *nadA* which codes for a minor adhesin in strain MC58.

**Figure S3:** qRT-PCR validation of *ex vivo* expression differences in selected putative virulence and regulatory genes.

The box-and-whiskers plot shows differences in the expression of the regulatory genes *relA*, *spoT* and *dksA* involved in the stringent response pathway, the alternative sigma factor gene *rpoE* as well as the genes of the denitrification pathway *norB* and *aniA*. For each gene tested in quadruplicate via qRT-PCR, the log2-fold changes are given with values smaller than 0 indicating a higher expression in MC58 than in α522. The experimental condition is given at the top of the panel. The dashed vertical lines correspond to at least 2-fold expression level differences.

**Figure S4:** Growth of strain α522 in minimal medium supplemented with different combinations of amino acids.

The abscissa of the bar plot depicts the absorbance (OD_600nm_) at 0 h, 4 h and 8 h of growth in meningococcal minimal medium (MMM) supplemented with different combinations of amino acids as indicated on the ordinate. The insert gives the amino acid concentrations used in the growth experiments, and the error bars the standard error from three independent measurements. Strain MC58 was used as positive control as it is known to grow in MMM [27].

**Figure S5:** Comparison of the stringent response in *N. meningitidis* strain MC58 and α522.

(A) Bar plot of ppGpp levels measured via HPLC in rich medium (PPM+) in the mid (ML) and late logarithmic (LL) growth phase for wild-type and mutant strains as indicated at the bottom of the panel.

(B) The position of a premature translational stop codon prior to the RelA catalytic domain in a Δ*spoT*::Cm^r^ mutant is indicated by a vertical line. The truncated RelA1 protein and the corresponding deletion leading to the frameshift in the Δ*spoT*::Cm^r^ mutant strain are depicted above, each aligned with the corresponding α522 wild-type sequences.

(C) Bar plot of ppGpp levels for the wild-type and the *relA1* Δ*spoT*::Cm^r^ strains, respectively, measured via HPLC in PPM+ in the LL growth phase.

All ppGpp measurements shown in panels A and C were done in duplicate.

**Figure S6:** Genetic map of the *relA* (A) and *spoT* (B) loci in the mutant strains.

**Figure S7:** Quality assessment of total RNA and microarray data.

(A) Bioanalyzer image of the RNA samples used for comparative transcriptomics extracted 30 min after infection.

(B) Bioanalyzer density plots of RNA samples isolated from whole blood.

(C) 2D-scatterplot testing for a possible correlation between sequence similarity and expression level differences.

**Figure S8:** Discriminator regions in genes differently expressed in different *ex vivo* conditions in MC58.

Scatter plot of the GC content variation averaged over a 5-bp sliding window for genes differently expressed in MC58 between saliva and blood (A) and blood and CSF (B), respectively. The black line gives the GC content of the respective upstream regions for genes not differently expressed. Regulatory regions are indicated at the top of the panel based on the average length of 5’-untranslated regions in *N. gonorrhoeae* [24]. The insert gives the number of genes in each gene set.

**Supplemental Tables**

**Table S1:** Strain α522 specific genes.

| **Locus** | **Gene** | **Product Name** | **COG** | **Genomic context** |
| --- | --- | --- | --- | --- |
| NMalpha522_0085 | *csb* | Polysialic acid capsule biosynthesis protein | M | artificial insertion of *ermC* in MC58 ^(1)^ |
| NMalpha522_0091 | *lcbA* | Capsule phosphotransferase | X | *cps* locus ^(7)^ |
| NMalpha522_0239 | *-* | Hypothetical protein | X | MME*pyrD* ^(1)^ |
| NMalpha522_0240 | *-* | Hypothetical protein | X | MME*pyrD* ^(1)^ |
| NMalpha522_0346 | *-* | Hypothetical protein | X | insertion/repeat |
| NMalpha522_0390 | *-* | Hypothetical protein | X | cMME*aroEglnA* ^(1)^ |
| NMalpha522_0395 | *-* | Hypothetical protein | X | region of degeneration ^(1)^ |
| NMalpha522_0399 | *-* | Hypothetical protein | X | *maf1* locus ^(2)^ |
| NMalpha522_0403 | *-* | Hypothetical protein | X | *maf1* locus ^(2)^ |
| NMalpha522_0404 | *-* | Hypothetical protein | X | *maf1* locus ^(2)^ |
| NMalpha522_0405 | *-* | Hypothetical protein | X | *maf1* locus ^(2)^ |
| NMalpha522_0406 | *-* | Hypothetical protein | X | *maf1* locus ^(2)^ |
| NMalpha522_0407 | *-* | Hypothetical protein | X | *maf1* locus ^(2)^ |
| NMalpha522_0408 | *-* | Hypothetical protein | X | *maf1* locus ^(2)^ |
| NMalpha522_0495 | *-* | Fic family protein | S | insertion |
| NMalpha522_0565 | *-* | Hypothetical protein | X | insertion/repeat |
| NMalpha522_1176 | *-* | Putative DNA-binding protein | S | insertion, prophage NeisMu1 |
| NMalpha522_1418 | *-* | Hypothetical protein | X | insertion |
| NMalpha522_1419 | *-* | Hypothetical protein | X | insertion |
| NMalpha522_1479 | *-* | Hypothetical protein | X | insertion |
| NMalpha522_1527 | *-* | Hypothetical protein | X | insertion |
| NMalpha522_1561 | *-* | Hypothetical protein | X | MME*bioDubiA* ^(1)^ |
| NMalpha522_1569 | *-* | Phosphate import ATP-binding protein | R | MME*bioDubiA* ^(1)^ |
| NMalpha522_1625 | *-* | Putative lipoprotein | X | MME*pyrC* ^(1)^ |
| NMalpha522_1659 | *-* | Hypothetical protein | X | *maf2* locus ^(2)^ |
| NMalpha522_1688 | *czcD* | Cadmium, cobalt and zinc/H(+)-K(+) antiporter | P | cMME*pgp* |
| NMalpha522_1833 | *-* | Hypothetical protein | X | cMME*argCrecG* ^(1)^ |
| NMalpha522_1849 | *-* | Hypothetical protein | X | *IS*5 composite transposon |
| NMalpha522_1850 | *-* | HlyD family secretion protein | V | *IS*5 composite transposon |
| NMalpha522_1851 | *-* | HlyB family ABC transporter | V | *IS*5 composite transposon |
| NMalpha522_1852 | *-* | Cupin family protein | S | *IS*5 composite transposon |
| NMalpha522_1853 | *ψrepA* | Plasmid partitioning protein pseudogene | D | *IS*5 composite transposon |
| NMalpha522_1879 | ψ*rfaG* | Glycosyltransferase pseudogene, N-terminus | M | MME*pglC* ^(1)^ |
| NMalpha522_2108 | *-* | Hypothetical protein | X | cMME*purLhagH* ^(1)^ |

^(1)^ Nomenclature according to Snyder LAS *et al.* (2007) [28]

^(2)^ Naming according to Bentley SD *et al.* (2007) [29]

^(3)^ Naming according to Tettelin H *et al.* (2000) [30]

^(4)^ Naming according to Kawai M *et al.* (2005) [31]

^(5)^ Naming according to Morgan GJ *et al.* (2002) [32]

^(6)^ Nomenclature according to Van Ulsen P and J Tommassen (2006) [33]

^(7)^ Nomenclature according to Harrison OB *et al.* (2013) [34]

**Table S2:** Oligonucleotides used in this study.

| **Name** | **ID** | **Sequence (5’-3’)^a^** | **Comment** |
| --- | --- | --- | --- |
| fumC-P1B | 161 | TCCCCGCCGTAAAAGCCCTG | qRT-PCR forward primer for NMB1458 |
| fumC-P2B | 162 | GCCCGTCAGCAAGCCCAAC | qRT-PCR reverse primer for NMB1458 |
| adh-P1B | 163 | CTGCCCCCGGGGTTTTCATCT | qRT-PCR forward primer for NMB1392 |
| adh-P2B | 164 | TGTTGCGCGTTATTTCAAAGAAGG | qRT-PCR reverse primer forNMB1392 |
| KB 9 | 242 | AATACGACTCACTATAGGGC | Amplification of an insert within pBS |
| 329 | 243 | ACCATGATTACGCCAAGC | Amplification of an insert within pBS |
| Kana1 | 244 | CACGAGGCAGACCTCAG | Amplification of down region of kanamycin resistance cassette |
| Kana2 | 245 | GATTTTGAGACACAACGTGG | Amplification of up region of the kanamycin resistance cassette |
| Nona | 837 | NNNNNNNNN | universal, non-specific primer |
| rpoC_RT(F) | 282 | GGATGATTATGGATGTGCTGCCG | qRT-PCR forward primer for NMB0133 |
| rpoC_RT(R) | 283 | CGCATGCAGTTCCAACAGACGTT | qRT-PCR reverse primer for NMB0133 |
| NMB1592_RT (F) | 238 | CCCCGTGAATCCTATCAATACCG | qRT-PCR forward primer for NMB1592 |
| NMB1592_RT(R) | 239 | CCGCCTGAACCTTGGTCATTTTA | qRT-PCR reverse primer for NMB1592 |
| dksA_RT(F) | 400 | GACGAACTCATCGAAAATGCTTCC | qRT-PCR forward primer for NMB0056 |
| dksA_RT(R) | 401 | GGTCGCCTGTATTTTACTGAGAAG | qRT-PCR reverse primer for NMB0056 |
| relA_RT(F) | 306 | ACCTATTGGTTTCCGAACGCTGC | qRT-PCR forward primer for NMB1735 |
| relA_RT(R) | 307 | GCCAGCAGCATTTTCCGCATAGT | qRT-PCR reverse primer for NMB1735 |
| spoT_RT(F) | 308 | AATATGCGGACGCTCGGTTCGAT | qRT-PCR forward primer for NMB1659 |
| spoT_RT(R) | 309 | TGGGATGCAGGTTTTGGAACGAT | qRT-PCR reverse primer for NMB1659 |
| 2144_RT_F | 362 | ATACAGCGCAGGCGACAGTTTTCA | qRT-PCR forward primer for NMB2144 |
| 2144_RT_R | 363 | GCTTCATCCAGTAGCTCGTCATCCA | qRT-PCR reverse primer for NMB2144 |
| ani_rt1 | 661 | ATGACCATGGAAGACGGTGTGGA | qRT-PCR forward primer for NMB1623 |
| ani_rt2 | 662 | TGGAAGTCGACGTTGTGCGGA | qRT-PCR reverse primer for NMB1623 |
| norB_RT 1 | 653 | TCCCCGATTGCCGTTATGGAA | qRT-PCR forward primer for NMB1622 |
| norB_RT 2 | 654 | CAAAGTGGAGGCAGTGGCGGTA | qRT-PCR reverse primer for NMB1622 |
| relA_Up (F) | 310 | gcgcgcGGATCCGATGCCGTCTGAAGCACCGTCCAT | *relA* deletion with BamHI restriction site |
| relA_Up (R) | 311 | gcgcgcCTGCAGGATGGGGTCTTCCGTTCCGATAAA | *relA* deletion with PstI restriction site |
| relA_Down (F) | 312 | gcgcgcCTGCAGATACAAAAATGCCGTCTGAAAGCC | *relA* deletion with PstI restriction site |
| relA_Down (R) | 313 | gcgcgcGAATTCTGTTTCATCCAAATAAACGCGTTG | *relA* deletion with EcoRI restriction site |
| ATR_Del1 | 324 | gcgcgcGGATCCCGATTCCGCCGTTGATGCCGTCTGA | ATR knock out with BamHI restriction site |
| ATR_Del2 | 325 | CCGATAAAAACGAGTTTATTGTAACAGCTTATTTATTTACGGTGCATAGGCGGG | Mega-prime pcr fragment |
| ATR_Del3 | 326 | CACCGTAAATAAATAAGCTGTTACAATAAACTCGTTTTTATCGGAACGGAAG | Mega-prime pcr fragment |
| ATR_Del4 | 327 | gcgcgcGGAATTCGGTTTCTTTGGCGACGGCGCGTTTTTC | ATR knock out with EcoRI restriction site |
| spoT_up_F | 354 | gcgcgcGGATCCACATTTGAAGCCATTGACCCTGT | *spoT* deletion with BamHI restriction site |
| spoT_up_R | 355 | gcgcgcAAGCTTAGCAGGTAAAACGGGTTGCCTT | *spoT* deletion with HindIII restriction site |
| spoT_Down_F | 356 | gcgcgcAAGCTTTTCAGACGGCTTTCGGTATGT | *spoT* deletion with HindIII restriction site |
| spoT_Down_R | 357 | gcgcgcATCGATAGGCTATGACGTGATTTTGGA | *spoT* deletion with ClaI restriction site |
| relA_GSP_1 | 360 | AGTGGGTGAGTTTCTGCACTTCGT | 5’-RACE primer I for *relA* |
| relA_GSP_2 | 361 | TACTGTTGCAGCGTTCGGAAACCA | 5’-RACE primer II for *relA* |
| atr_ki_1 | 410 | gcgcgcGGATCCATGCCGTCTGAATCAAATTGGCGGAAAAGCTGCCGATGCTT | ATR knock in with BamHI restriction site |
| atr_ki_2 | 411 | TAACAGACTATTTTTGCAAAGGTCTCAGCTTATTTATTTACGGTGCATA | Mega-prime pcr fragment |
| atr_ki_3 | 412 | TATGCACCGTAAATAAATAAGCTGAGACCTTTGCAAAAATAGTCTGTTA | Mega-prime pcr fragment |
| atr_ki_4 | 413 | TCCGATAAAAACGAGTTTATTGTAACAGCTTGAGACCTTTGCAATAACAT | Mega-prime pcr fragment |
| atr_ki_5 | 414 | ATGTTATTGCAAAGGTCTCAAGCTGTTACAATAAACTCGTTTTTATCGGA | Mega-prime pcr fragment |
| atr_ki_6 | 415 | gcgcgcAAGCTTATGCCGTCTGAAATCGCCAGTTTGATTAACACGACGCGGATG | ATR knock in with HindIII restriction site |
| grx_Mega_2 | 419 | GCTGTAAAACGACGGCCAGTAAGCTTAAGTGGCTGTTATTAACTGACAGATG | Mega-prime pcr fragment |
| grx_Mega_3 | 420 | CATCTGTCAGTTAATAACAGCCACTTAAGCTTACTGGCCGTCGTTTTACAGC | Mega-prime pcr fragment |
| grx_Mega_4 | 421 | CGACAGGTTGACGAGGAATATTTTGAAGCTTCTAGAGATCCGGTTTTTGT | Mega-prime pcr fragment |
| grx_Mega_5 | 422 | ACAAAAACCGGATCTCTAGAAGCTTCAAAATATTCCTCGTCAACCTGTCG | Mega-prime pcr fragment |
| grx_Mega_1_neu | 424 | gcgcgcCTCGAGATTCAAGAATCTAGCCAAGATA | *grxB* deletion with XhoI restriction site |
| grx_Mega_6_neu | 425 | gcgcgcCTCGAGAGTTTGATTAACACGACGCGGATGT | *grxB* deletion with XhoI restriction site |
| grx_GSP1 | 453 | AACTCTCGCCCATAAACGAACCGT | 5’-RACE primer I for *grxB* |
| grx_GSP2 | 454 | TTTCGCACCGATCATACCGATCG | 5’-RACE primer II for *grxB* |

^a^Restriction sites are underlined, small letters indicate nonsense-nucleotides

### **Table S3:** Plasmids used in this study.

| **Name** | **ID** | **Description** | **Resistance** | **Reference** |
| --- | --- | --- | --- | --- |
| pUC4K | 58 | Delivery plasimd harbouring kanamycin resistance cassette | Km^r^ |  |
| pBluescript-SK | 315 | Cloning vector | Amp^r^ | Stratagene |
| pBS-CM | 2304 | Delivery plasimd harbouring chloramphenicol resistance cassette | Cm^r^ | IHM, unpublished |
| pKE1 | 4713 | Construct for generating deletion mutant of the NMB1735 *(relA)* gene in *Nm* MC58 | Amp^r^Km^r^ | This study |
| pKE2 | 4714 | Construct for generating deletion mutant of the *relA* gene in *Nm* α522 | Amp^r^Km^r^ | This study |
| pKE3 | 4734 | Construct for generating deletion mutant of ATR*_relA_* in *Nm* α522 | Amp^r^ | This study |
| pKE4 | 4736 | Construct for generating deletion mutant of the NMB1659 *(spoT)* gene in *Nm* MC58∆*relA::Km^r^*  and α522∆*relA::Km^r^* | Amp^r^ Cm^r^ | This study |
| pKB1 | 4754 | Construct for generating insertion mutant of ATR*_relA_* in *Nm* MC58 | Amp^r^ | This study |
| pKB2 | 4756 | Construct for generating deletion mutant of the *grxB* gene in *Nm* MC58 | Amp^r^ Cm^r^ | This study |
| pKB3 | 4757 | Construct for generating deletion mutant of the *grxB* gene in *Nm* α522 | Amp^r^ Cm^r^ | This study |

**Table S4:** Strains used in this study.

| **Parental strain** | **Strain ID** | **Genotype/relevant characteristics** | **Source/Reference** |
| --- | --- | --- | --- |
| *Escherischia coli* | |  |  |
| TOP10 | 4342 | F– mcrA Δ(mrr-hsdRMS-mcrBC) Φ80lacZΔM15 ΔlacX74 recA1 araD139 Δ(ara leu) 7697 galU galK rpsL (StrR) endA1 nupG | Invitrogen |
| *Neisseria meningitidis* | |  |  |
| α522 | 522 | wild-type | IHM |
| MC58 | WUE2135 | wild-type | McGuiness et al., 1991 |
| α522 | WUE4715 | ∆*relA::Km^r^* | This study |
| MC58 | WUE4716 | ∆*relA::Km^r^* | This study |
| α522 | WUE4733 | ΔATR*_relA_* | This study |
| MC58 | WUE4737 | *relA1* ∆*spoT::Cm^r^* | This study |
| MC58 | WUE4739 | ∆*relA::Km^r^*∆*spoT::Cm^r^* | This study |
| α522 | WUE4740 | ∆*relA::Km^r^*∆*spoT::Cm^r^* | This study |
| MC58 | WUE4755 | *relAp*::ATR*_relA_* | This study |
| α522 | WUE4758 | ∆*grxB::Cm^r^* | This study |
| MC58 | WUE4759 | ∆*grxB::Cm^r^* | This study |
