# Supplementary figures and images for: Transcriptomic buffering of cryptic genetic variation contributes to meningococcal virulence
Source: BMC Genomics. 2017 Apr 7;18:282. doi: 10.1186/s12864-017-3616-7 (PMC5383966; doi:10.1186/s12864-017-3616-7)

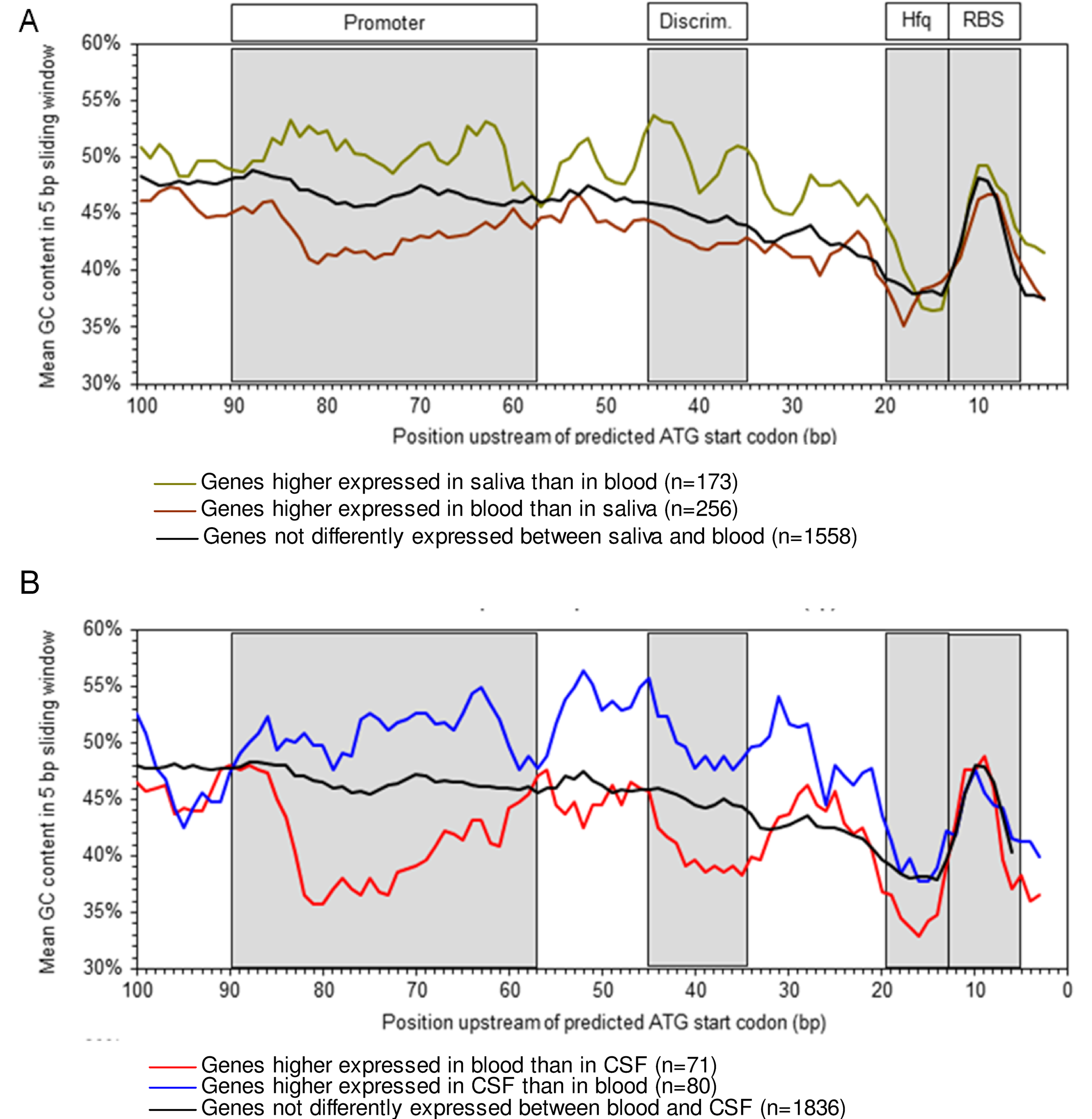

Supplement: Supplementary file 1 — Contains supplemental results and discussion describing the results of ex vivo cross-condition gene expression comparisons in strain MC58 along with the corresponding supplemental references and the figure legends to the supplemental Figures S1 to S8 as well as the supplemental Tables S1 to S4. Figure S1. Experimental setup of the study. Figure S2. Comparison of the N. meningitidis α522 and MC58 genomes. Figure S3. qRT-PCR validation of ex vivo cross-strain expression differences in selected putative virulence-associated and regulatory genes. Figure S4. Growth of strain α522 in minimal medium supplemented with different combinations of amino acids. Figure S5. Comparison of the stringent response in N. meningitidis strain MC58 and α522. Figure S6. Genetic map of the relA and spoT loci in the mutant strains. Figure S7. Quality assessment of total RNA and microarray data. Figure S8. Discriminator regions in genes differently expressed in different ex vivo conditions in MC58. Table S1. Strain α522 specific genes. Table S2. Oligonucleotides used in this study. Table S3. Plasmids used in this study. Table S4. Strains used in this study. (ZIP 19627 kb) [file 12864_2017_3616_MOESM1_ESM.zip › Figure S8 new.tif]

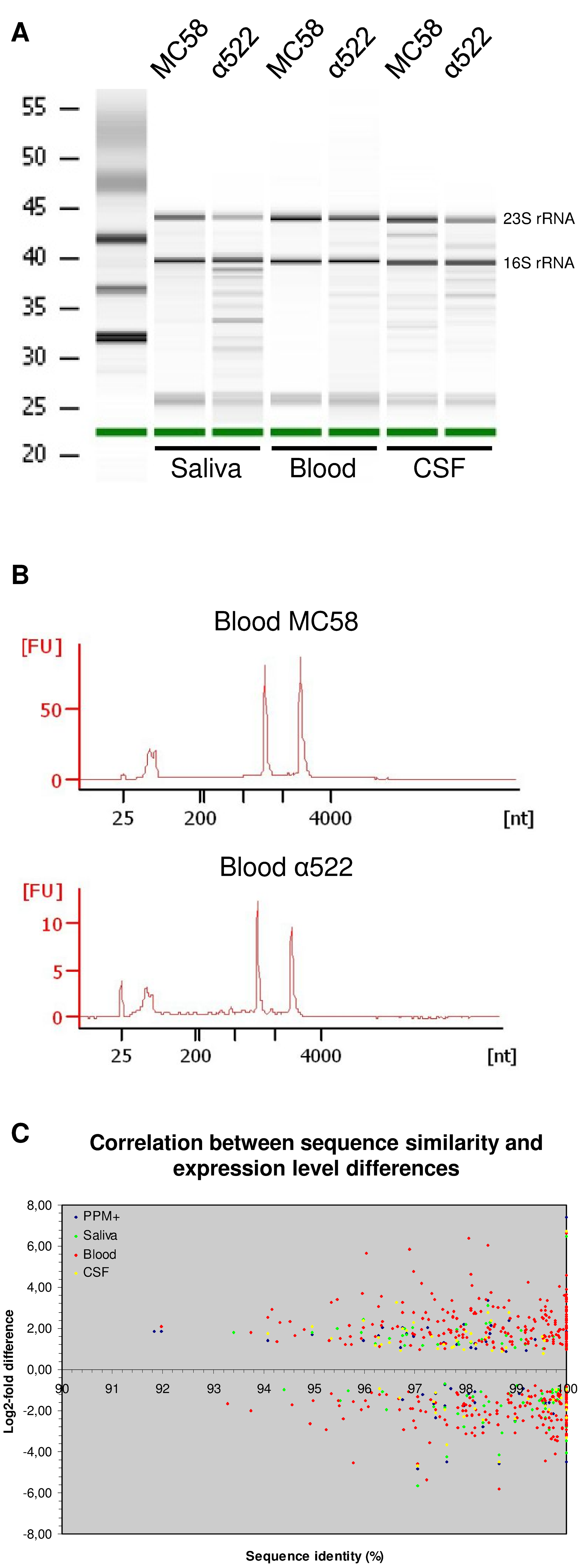

Supplement: Supplementary file 1 — Contains supplemental results and discussion describing the results of ex vivo cross-condition gene expression comparisons in strain MC58 along with the corresponding supplemental references and the figure legends to the supplemental Figures S1 to S8 as well as the supplemental Tables S1 to S4. Figure S1. Experimental setup of the study. Figure S2. Comparison of the N. meningitidis α522 and MC58 genomes. Figure S3. qRT-PCR validation of ex vivo cross-strain expression differences in selected putative virulence-associated and regulatory genes. Figure S4. Growth of strain α522 in minimal medium supplemented with different combinations of amino acids. Figure S5. Comparison of the stringent response in N. meningitidis strain MC58 and α522. Figure S6. Genetic map of the relA and spoT loci in the mutant strains. Figure S7. Quality assessment of total RNA and microarray data. Figure S8. Discriminator regions in genes differently expressed in different ex vivo conditions in MC58. Table S1. Strain α522 specific genes. Table S2. Oligonucleotides used in this study. Table S3. Plasmids used in this study. Table S4. Strains used in this study. (ZIP 19627 kb) [file 12864_2017_3616_MOESM1_ESM.zip › Figure S7 new.tif]

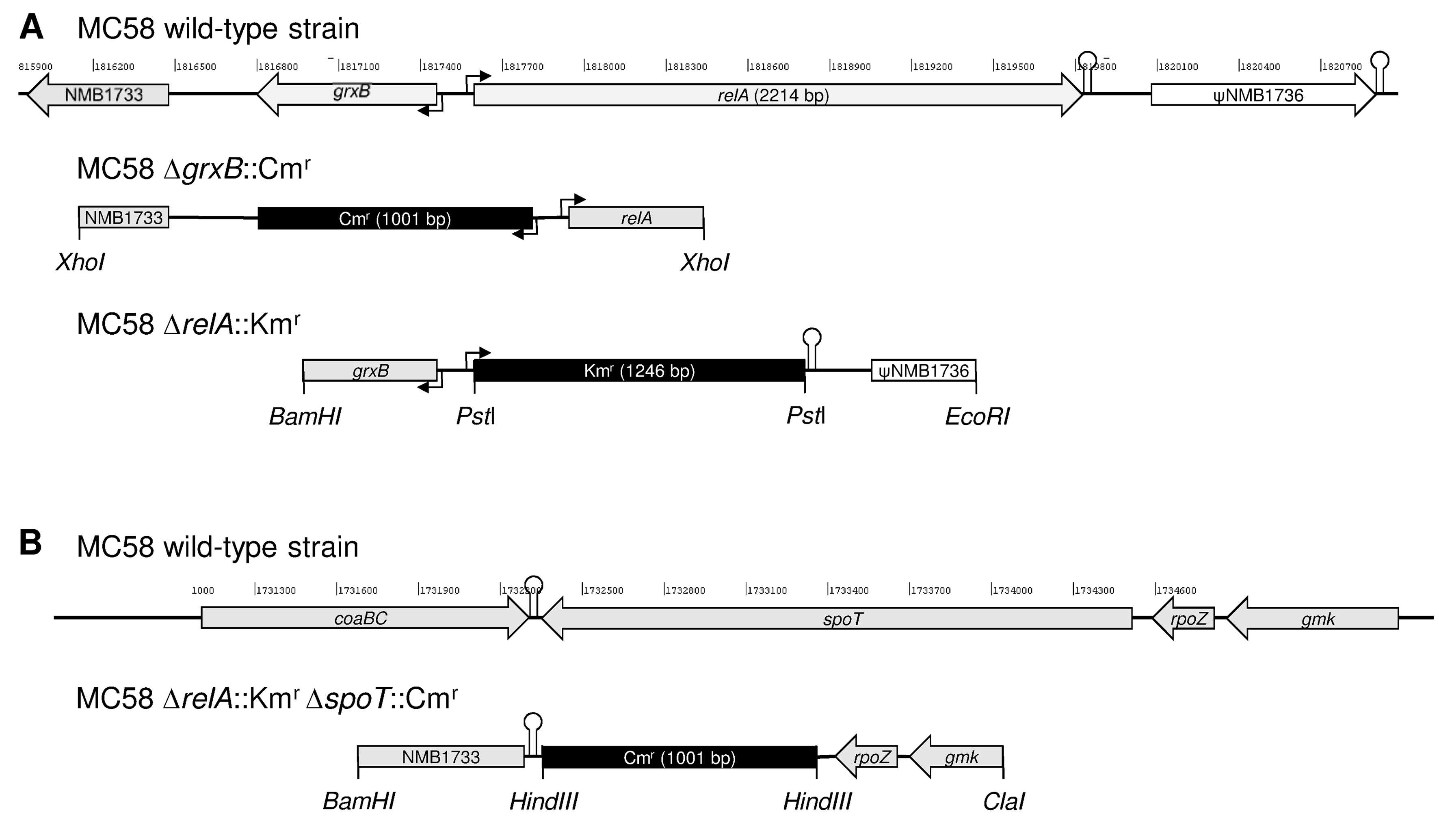

Supplement: Supplementary file 1 — Contains supplemental results and discussion describing the results of ex vivo cross-condition gene expression comparisons in strain MC58 along with the corresponding supplemental references and the figure legends to the supplemental Figures S1 to S8 as well as the supplemental Tables S1 to S4. Figure S1. Experimental setup of the study. Figure S2. Comparison of the N. meningitidis α522 and MC58 genomes. Figure S3. qRT-PCR validation of ex vivo cross-strain expression differences in selected putative virulence-associated and regulatory genes. Figure S4. Growth of strain α522 in minimal medium supplemented with different combinations of amino acids. Figure S5. Comparison of the stringent response in N. meningitidis strain MC58 and α522. Figure S6. Genetic map of the relA and spoT loci in the mutant strains. Figure S7. Quality assessment of total RNA and microarray data. Figure S8. Discriminator regions in genes differently expressed in different ex vivo conditions in MC58. Table S1. Strain α522 specific genes. Table S2. Oligonucleotides used in this study. Table S3. Plasmids used in this study. Table S4. Strains used in this study. (ZIP 19627 kb) [file 12864_2017_3616_MOESM1_ESM.zip › Figure S6 new.tif]

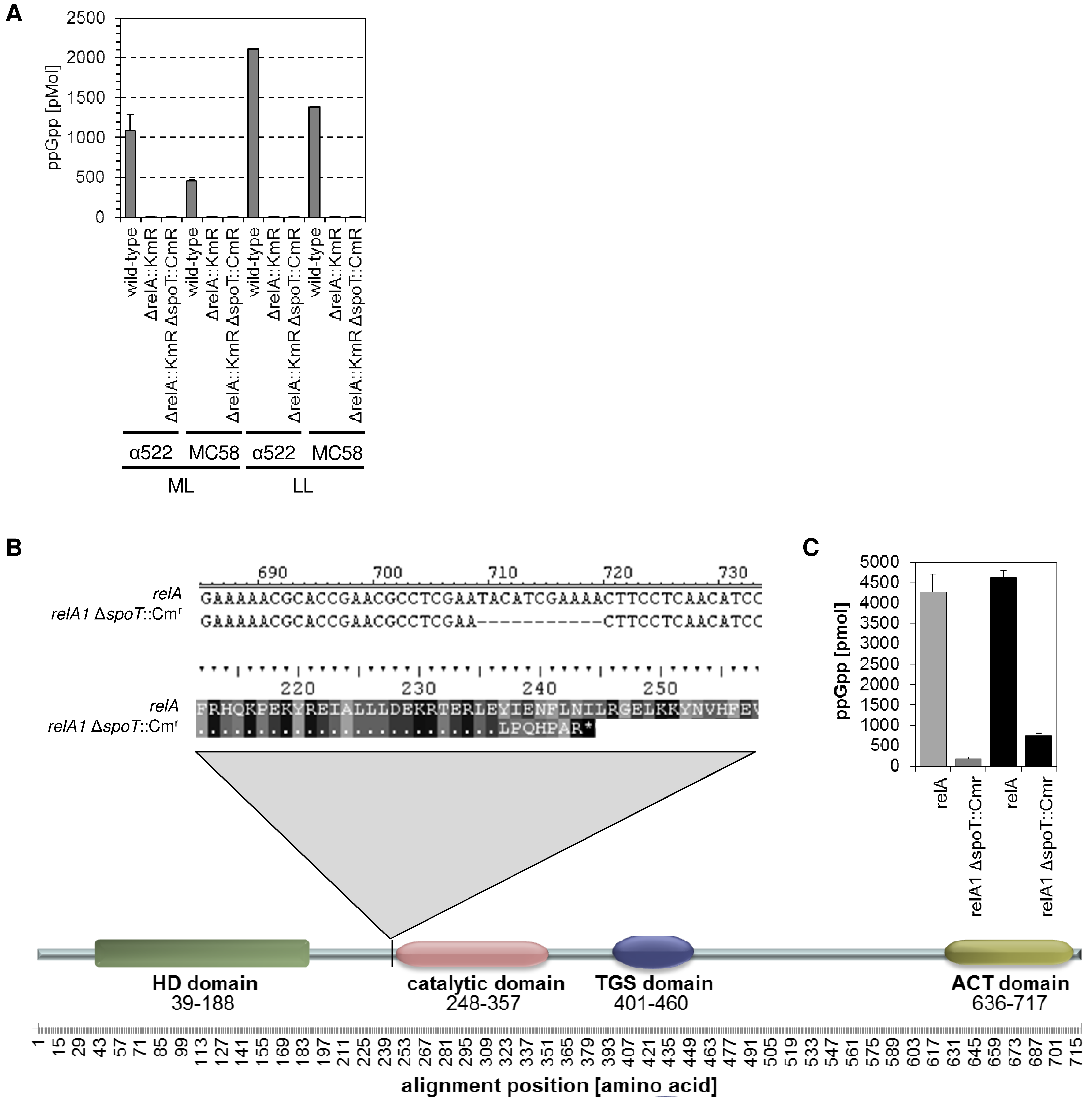

Supplement: Supplementary file 1 — Contains supplemental results and discussion describing the results of ex vivo cross-condition gene expression comparisons in strain MC58 along with the corresponding supplemental references and the figure legends to the supplemental Figures S1 to S8 as well as the supplemental Tables S1 to S4. Figure S1. Experimental setup of the study. Figure S2. Comparison of the N. meningitidis α522 and MC58 genomes. Figure S3. qRT-PCR validation of ex vivo cross-strain expression differences in selected putative virulence-associated and regulatory genes. Figure S4. Growth of strain α522 in minimal medium supplemented with different combinations of amino acids. Figure S5. Comparison of the stringent response in N. meningitidis strain MC58 and α522. Figure S6. Genetic map of the relA and spoT loci in the mutant strains. Figure S7. Quality assessment of total RNA and microarray data. Figure S8. Discriminator regions in genes differently expressed in different ex vivo conditions in MC58. Table S1. Strain α522 specific genes. Table S2. Oligonucleotides used in this study. Table S3. Plasmids used in this study. Table S4. Strains used in this study. (ZIP 19627 kb) [file 12864_2017_3616_MOESM1_ESM.zip › Figure S5.tif]

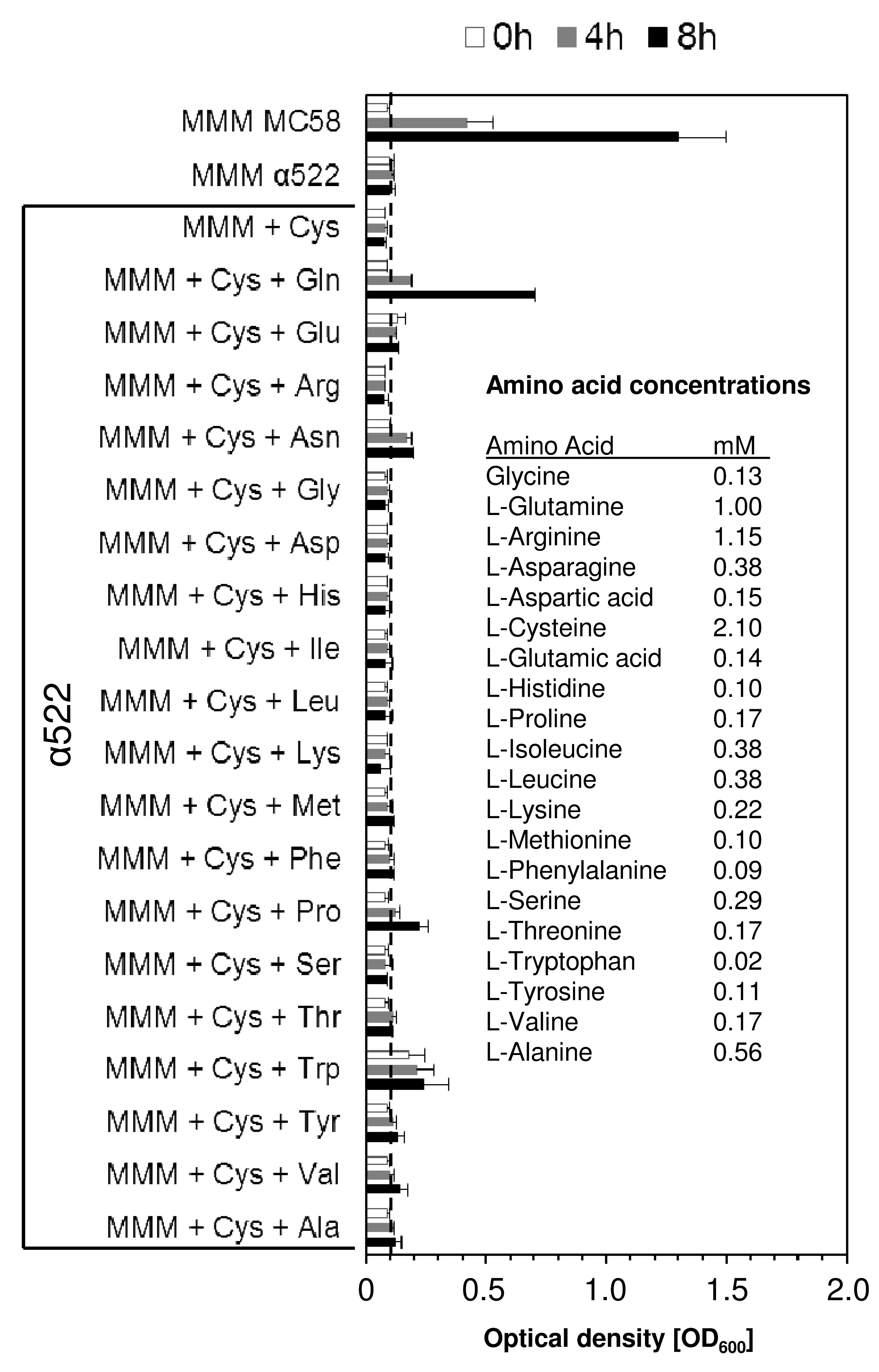

Supplement: Supplementary file 1 — Contains supplemental results and discussion describing the results of ex vivo cross-condition gene expression comparisons in strain MC58 along with the corresponding supplemental references and the figure legends to the supplemental Figures S1 to S8 as well as the supplemental Tables S1 to S4. Figure S1. Experimental setup of the study. Figure S2. Comparison of the N. meningitidis α522 and MC58 genomes. Figure S3. qRT-PCR validation of ex vivo cross-strain expression differences in selected putative virulence-associated and regulatory genes. Figure S4. Growth of strain α522 in minimal medium supplemented with different combinations of amino acids. Figure S5. Comparison of the stringent response in N. meningitidis strain MC58 and α522. Figure S6. Genetic map of the relA and spoT loci in the mutant strains. Figure S7. Quality assessment of total RNA and microarray data. Figure S8. Discriminator regions in genes differently expressed in different ex vivo conditions in MC58. Table S1. Strain α522 specific genes. Table S2. Oligonucleotides used in this study. Table S3. Plasmids used in this study. Table S4. Strains used in this study. (ZIP 19627 kb) [file 12864_2017_3616_MOESM1_ESM.zip › Figure S4.tif]

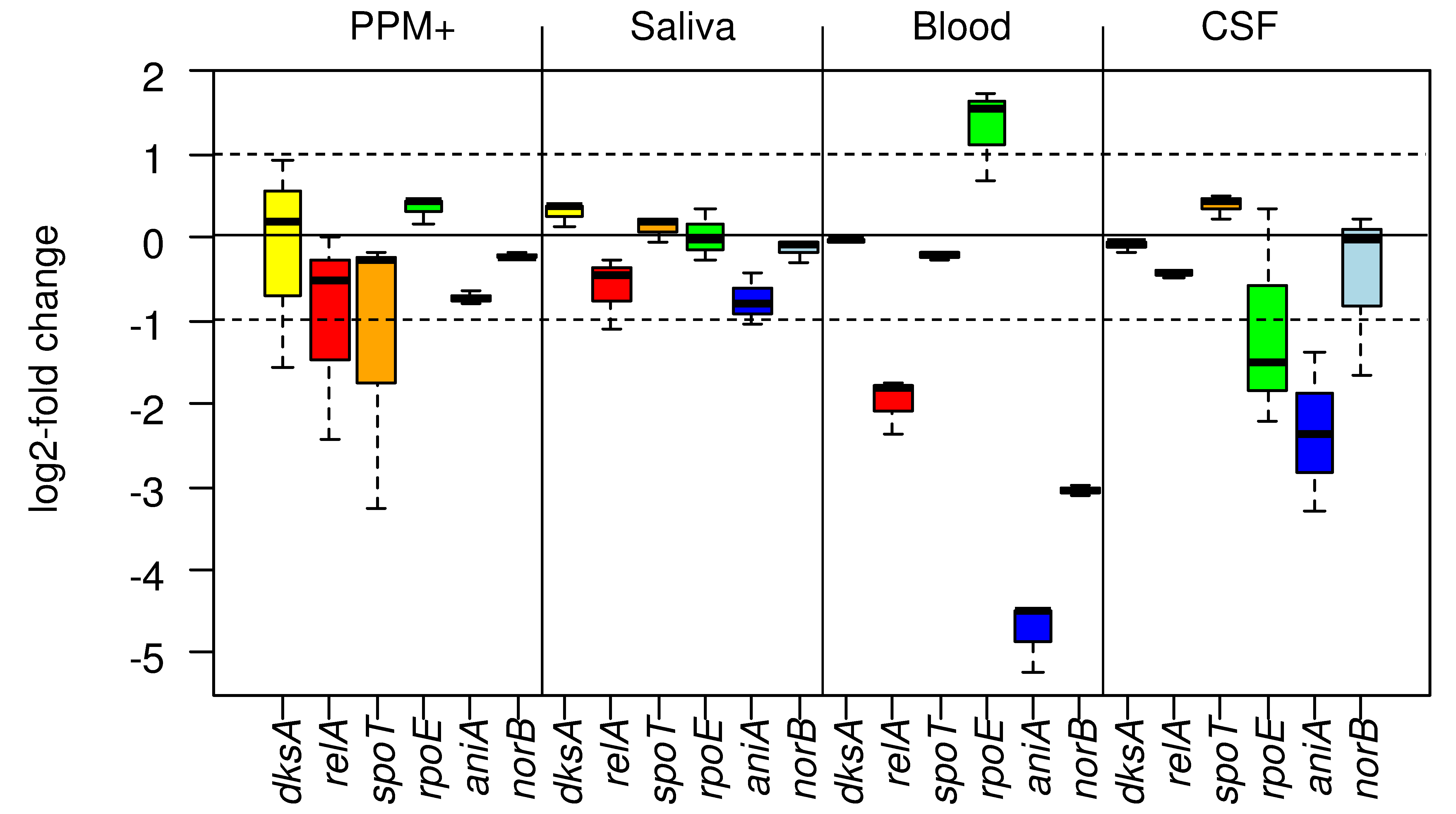

Supplement: Supplementary file 1 — Contains supplemental results and discussion describing the results of ex vivo cross-condition gene expression comparisons in strain MC58 along with the corresponding supplemental references and the figure legends to the supplemental Figures S1 to S8 as well as the supplemental Tables S1 to S4. Figure S1. Experimental setup of the study. Figure S2. Comparison of the N. meningitidis α522 and MC58 genomes. Figure S3. qRT-PCR validation of ex vivo cross-strain expression differences in selected putative virulence-associated and regulatory genes. Figure S4. Growth of strain α522 in minimal medium supplemented with different combinations of amino acids. Figure S5. Comparison of the stringent response in N. meningitidis strain MC58 and α522. Figure S6. Genetic map of the relA and spoT loci in the mutant strains. Figure S7. Quality assessment of total RNA and microarray data. Figure S8. Discriminator regions in genes differently expressed in different ex vivo conditions in MC58. Table S1. Strain α522 specific genes. Table S2. Oligonucleotides used in this study. Table S3. Plasmids used in this study. Table S4. Strains used in this study. (ZIP 19627 kb) [file 12864_2017_3616_MOESM1_ESM.zip › Figure S3.tif]

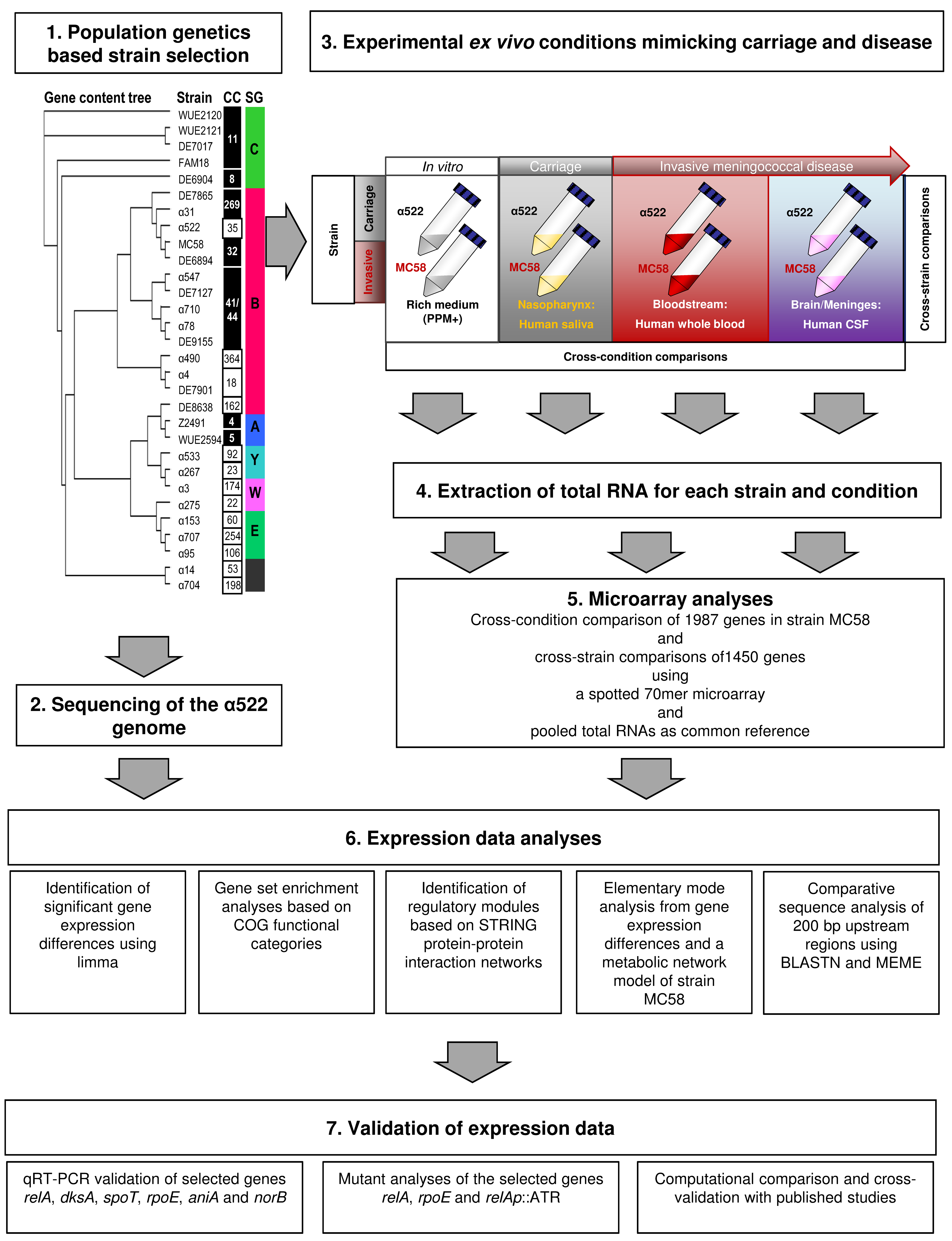

Supplement: Supplementary file 1 — Contains supplemental results and discussion describing the results of ex vivo cross-condition gene expression comparisons in strain MC58 along with the corresponding supplemental references and the figure legends to the supplemental Figures S1 to S8 as well as the supplemental Tables S1 to S4. Figure S1. Experimental setup of the study. Figure S2. Comparison of the N. meningitidis α522 and MC58 genomes. Figure S3. qRT-PCR validation of ex vivo cross-strain expression differences in selected putative virulence-associated and regulatory genes. Figure S4. Growth of strain α522 in minimal medium supplemented with different combinations of amino acids. Figure S5. Comparison of the stringent response in N. meningitidis strain MC58 and α522. Figure S6. Genetic map of the relA and spoT loci in the mutant strains. Figure S7. Quality assessment of total RNA and microarray data. Figure S8. Discriminator regions in genes differently expressed in different ex vivo conditions in MC58. Table S1. Strain α522 specific genes. Table S2. Oligonucleotides used in this study. Table S3. Plasmids used in this study. Table S4. Strains used in this study. (ZIP 19627 kb) [file 12864_2017_3616_MOESM1_ESM.zip › Figure S1.tif]
